# Comparative analysis of human, rodent and snake deltavirus replication

**DOI:** 10.1371/journal.ppat.1012060

**Published:** 2024-03-05

**Authors:** Pierre Khalfi, Zoé Denis, Joe McKellar, Giovanni Merolla, Carine Chavey, José Ursic-Bedoya, Lena Soppa, Leonora Szirovicza, Udo Hetzel, Jeremy Dufourt, Cedric Leyrat, Nora Goldmann, Kaku Goto, Eloi Verrier, Thomas F. Baumert, Dieter Glebe, Valérie Courgnaud, Damien Gregoire, Jussi Hepojoki, Karim Majzoub

**Affiliations:** 1 Institut de Génétique Moléculaire de Montpellier, University of Montpellier, CNRS, Montpellier, France; 2 Department of hepato-gastroenterology, Hepatology and Liver Transplantation Unit, Saint Eloi University Hospital, Montpellier, France; 3 Institute of Medical Virology, National Reference Centre for Hepatitis B Viruses and Hepatitis D Viruses, German Center for Infection Research (DZIF, Partner Site Giessen-Marburg-Langen), Justus Liebig University Giessen, Giessen, Germany; 4 Medicum, Department of Virology, University of Helsinki, Helsinki, Finland; 5 Institute of Veterinary Pathology, Vetsuisse Faculty, University of Zürich, Zürich, Switzerland; 6 Institut de Recherche en Infectiologie de Montpellier (IRIM), Université de Montpellier, CNRS UMR9004, Montpellier, France; 7 Institut de Génomique Fonctionnelle, Université de Montpellier, CNRS, INSERM, Montpellier, France; 8 Université de Strasbourg, Inserm, Institut de Recherche sur les Maladies Virales et Hépatiques UMR_S1110, Strasbourg, France; 9 Institut Hospitalo-Universitaire, Pôle Hépato-digestif, Nouvel Hôpital Civil, Strasbourg, France; University of California, San Diego, UNITED STATES

## Abstract

The recent discovery of Hepatitis D (HDV)*-like* viruses across a wide range of taxa led to the establishment of the *Kolmioviridae* family. Recent studies suggest that kolmiovirids can be satellites of viruses other than Hepatitis B virus (HBV), challenging the strict HBV/HDV-association dogma. Studying whether kolmiovirids are able to replicate in any animal cell they enter is essential to assess their zoonotic potential. Here, we compared replication of three kolmiovirids: HDV, rodent (RDeV) and snake (SDeV) deltavirus *in vitro* and *in vivo*. We show that SDeV has the narrowest and RDeV the broadest host cell range. High resolution imaging of cells persistently replicating these viruses revealed nuclear viral hubs with a peculiar RNA-protein organization. Finally, *in vivo* hydrodynamic delivery of viral replicons showed that both HDV and RDeV, but not SDeV, efficiently replicate in mouse liver, forming massive nuclear viral hubs. Our comparative analysis lays the foundation for the discovery of specific host factors controlling *Kolmioviridae* host-shifting.

## Author summary

Hepatitis D viruses (HDV) is a human pathogen responsible for severe liver disease. Although it was the only known member of its family for over 40 years, recent studies have discovered related viruses in a variety of animal hosts. To assess the potential of these newly discovered viruses to infect humans and animals distinct from their known hosts, we compare the replication of HDV, rodent (RDeV) and snake (SDeV) deltaviruses through various methods in cell culture and in a mouse model. We describe common and divergent features of these three viruses, showing that RDeV has a broad host range whereas SDeV does not. Finally, we describe RDeV replication *in vivo* for the first time. Our study paves the way for understanding the lifecycle of these newly discovered enigmatic satellite viruses.

## Introduction

For over 40 years, Hepatitis D virus (HDV) was the only known member of the unassigned genus *Deltavirus* [1–3]. Originally discovered in a Hepatitis B virus (HBV) infected patient, HDV was later shown to be a satellite of HBV [4,5], a major cause of liver disease and cancer. Recently, the coincidental identification of HDV*-like* elements, in bird cloaca [6] and snake brains [7], provided the first evidence that HDV is not the sole representative of deltaviruses. Since then, several independent meta-transcriptomic studies have identified HDV-*like* sequences in a variety of samples originating from bats, rats, deer, marmots, birds, frogs, fishes and insects[8–12]. These discoveries indicated that this new viral family is far more diverse and widespread amongst the animal kingdom than originally thought. Evidence that HDV*-like* viruses found in snakes [13,14], rodents[9,11] and birds [11] are able to replicate and the recent identification of thousands of sequences similar to deltaviruses in metatranscriptomes[12,15] led to the establishment of a novel realm, *Ribozyviria*, with a single family, *Kolmioviridae*, that includes the genus *Deltavirus* as well as seven other novel genera of kolmiovirids [16]. Because other kolmiovirids were only recently discovered, most of our knowledge of the biology of these agents stems from research on HDV [17].

HDV possesses a negative-stranded, circular and highly self-complementary RNA genome of ~1700 nucleotides, making it the smallest known virus able to infect animal cells [17,18]. An estimated 15 to 20 million individuals worldwide are HBV-HDV co-infected, and chronic co-infection is considered the most severe form of viral hepatitis, often leading to advanced liver disease and cancer[19–22]. HDV hijacks HBV surface antigens (HBsAg) for infectious particle formation and enters human hepatocytes via the sodium-taurocholate co-transporting polypeptide (NTCP) receptor, which dictates its liver tropism [23,24]. Once HDV gains access to host cells, viral transcription and replication are mediated by cellular RNA polymerases, independently of HBV [25,26]. The HDV genome encodes a single protein, the hepatitis delta antigen (HDAg) which exists in two forms, the small (S-HDAg) and the large (L-HDAg) that differs from S-HDAg by 19 additional amino acids (AAs) at its C-terminal end [27]. The S-HDAg is essential for viral RNA replication [28], while a farnesylated form of L-HDAg is involved in HDV assembly [29,30].

Although newly discovered kolmiovirids share similar genome size and organization with HDV, they appear to differ from HDV in many aspects. For instance, they are not restricted to the liver of infected animals. Swiss snake colony virus 1 (SwSCV-1) (hereafter referred to as Snake deltavirus or SDeV) was detected in the spleen, kidney, lung and brain of infected boa constrictors [7]. Likewise, Tome’s spiny-rat virus 1 (TSRV-1) (hereafter referred to as Rodent deltavirus or RDeV) was detected in the kidney, lung, heart and small intestine of infected spiny rats [9]. Importantly, none of the novel kolmiovirids have been linked to a *Hepadnaviridae* (HBV family) co-infection so far. In fact, Reptarena- and Hartmaniviruses, commonly found in captive constrictor snakes [31], were shown to act as helper viruses of SDeV [13]. Furthermore, HDV was recently shown to form infectious particles with envelope glycoproteins different from HBsAg (e.g. *Flaviviridae*, *Rhabdoviridae*) [32]. These observations lend support to the idea that kolmiovirids could potentially invade many cell types, when packaged with the appropriate viral envelope. Interestingly, a recent study proposed that these viruses are capable of host-shifting between highly divergent species, suggesting that the contemporary association between HDV and HBV likely arose following zoonotic transmission from a yet undiscovered animal reservoir [10].

The potential of these satellite viruses to enter many cell types coupled to their exclusive reliance on host factors for replication begs the question: Can kolmiovirids replicate in any cell type they access? Here, we try to address this question using HDV, RDeV and SDeV replicons and pseudotyped viral particles, to characterize their replication in a variety of animal cell lines and in an *in vivo* mouse model.

## Results

### Comparison of human, rodent and snake delta antigens (DAgs)

**Fig 1:**
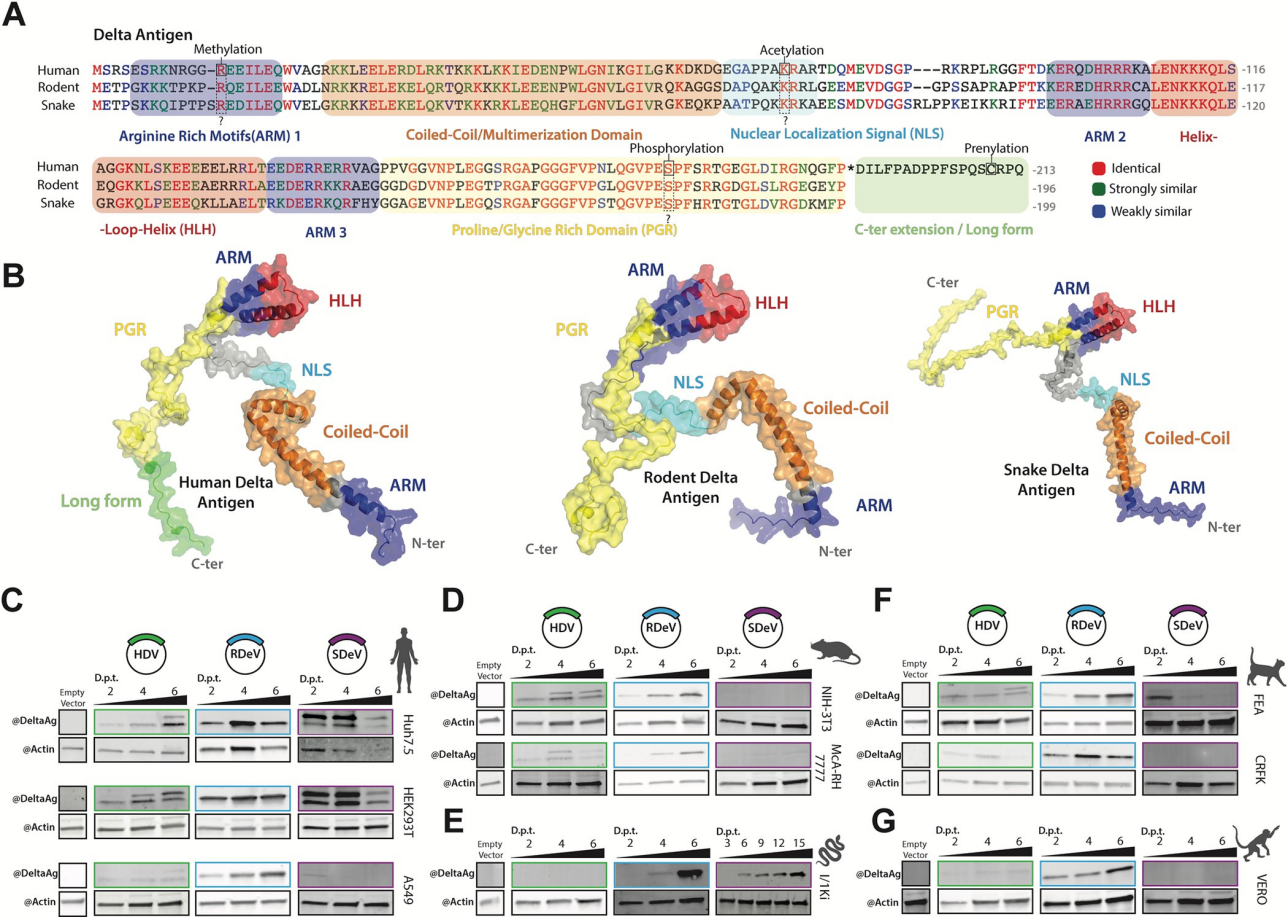
Comparison of HDV, RDeV and SDeV antigen amino acid sequences, predicted 3D structures and antigen accumulation in immortalized cell lines. A) Clustal Omega alignment of HDV, RDeV and SDeV amino acid sequences. Amino-acids are color coded by similarity, the characterized HDAg domains are indicated by the colored boxes and known post-translational modifications of HDAg are indicated by the dashed boxes. B) AlphaFold2 prediction of the 3D structure of human, rodent and snake delta antigens. The characterized HDV domains are indicated on all three antigens by color. (C-G) Deltavirus antigen accumulation in different immortalized cell lines. pcDNA3.1 plasmids encoding dimers of the human, rodent and snake deltavirus genomes, or an empty backbone (E.V.), were transfected in human (C), rodent (D), snake (E), cat (F) and monkey (G) cell lines. Cells were collected at either 2-, 4- and 6- or 3-, 6-, 9-, 12- and 15-days (SDeV/ I/1Ki) post-transfection (d.p.t.) and delta antigen expression was analyzed by western blot. β-actin serves as a loading control.

HDAg harbors seven previously mapped regions: three Arginine Rich Motifs (ARMs), a nuclear localization signal (NLS), a coiled-coil domain, a helix-loop-helix motif (HLH) and a proline- and glycine-rich (PGR) region at the C-terminus [33–37] (Figs 1A and S1B). Furthermore, post-translational modification of three AA residues in the S-HDAg have been shown to be important for HDV replication: Arg-13 methylation [38], Lys-72 acetylation [39] and Ser-177 phosphorylation [40].

We first sought to map known domains and modified residues present in HDAg, to conserved regions in rodent and snake DAgs, both on the primary AA sequence and on putative 3D structural models. To do so we performed: 1- a multiple AA alignment of HDAg (genotype 1 –HDV isolate Taylor), RDAg (RDeV isolate 183) and SDAg (SDeV isolate F18-5) (Fig 1A) from which we calculated a sequence conservation score (S1A–S1B Fig), 2- an analysis of intrinsic disorder along the sequence of each DAg (S1B Fig) and 3- 3D structure predictions of each of the three antigens using AlphaFold2 (AF2), mapping different functional and structural motifs onto the structural models of HDAg, RDAg and SDAg monomers (Figs 1B and S1B). Overall, the analysis of sequence conservation showed that while HDAg and RDAg are more closely related, sharing 57% AA identity (with 69% similarity), SDAg is slightly more divergent with 49 and 56% AA identity to HDAg and RDAg respectively, but equivalent in similarity (66 and 70% respectively) (S1A Fig). The comparison of sequence conservation, structural disorder and AF2 confidence score profiles shows a conserved modular architecture of the DAgs (Figs 1B and S1B). The coiled-coil domain and HLH motif display strong sequence conservation and form stable structures that are predicted with high confidence by AF2 (70 to 90 pLDDT score) (Figs 1B and S1B). Based on the disorder score, the HLH motif appears less stable than the coiled-coil domain, and is flanked by two intrinsically disordered regions encompassing the NLS at its N-terminus and the PGR at its C-terminus. Interestingly, the NLS is the least conserved region between each DAg, while the most conserved stretch of residues is in the PGR (>70% AA identity for AA 150–190 of HDAg) (S1B Fig). Furthermore, the combination of high conservation, low disorder score and low AF2 confidence score in the PGR (S1B Fig) suggests that this region may contain a short linear interaction motif that folds upon interaction with protein and/or RNA partners. Importantly, residues Arg-13[38], Lys-72[39] and Ser-177[40] known to be post-translationally modified in HDAg, are conserved in both RDAg and SDAg (Fig 1A). Taken together, our *in silico* analysis reveals a common modular architecture of DAgs, and the conservation of important structural and functional motifs, as well as post-translationally modified AA residues previously identified in HDAg. These results suggest that similar functional motifs and post-translational modifications might govern kolmiovirid replication.

### Antibody cross-reactivity for detection of kolmiovirid DAgs

Kolmiovirid DAgs share sequence homology and therefore potential epitopes for antibody-based detection [9]. To allow reliable detection of DAgs, we tested different antisera for their cross-reactivity: six obtained from HDV-positive patients [41,42] and one from a rabbit immunized with recombinant SDAg [7]. We cloned in mammalian expression vectors HDV, RDeV, SDeV, Chusan Island toad virus 1 (CITV-1, referred to as Toad deltavirus), and dabbling duck virus 1 (DabDV-1, referred to as Avian deltavirus) DAgs with a C-terminal FLAG-tag. Ectopic expression of these constructs in Huh7 cells, followed by immunoblotting, revealed that all tested antisera were able to cross-react and detect HDV, RDeV, SDeV and DabDV-1 DAgs (S1C Fig). However, CITV-1 (toad) DAg was only detected by 2 out of the 7 tested antisera (S1C Fig), in agreement with the phylogenetic divergence of this protein (S1D Fig). Therefore, we confirm the use of available antisera to reliably detect kolmiovirid DAgs and thus to compare and characterize the replication of RDeV and SDeV.

### HDV, RDeV and SDeV replication in human and animal cell lines

Although most studies have reported HDV replication in human hepatocytes, other kolmiovirids are not restricted to the liver of their animal hosts [7,9]. Moreover, HDV’s and SDeV’s capacity to form infectious particles using envelope proteins of various viruses [13,32] allows kolmiovirid entry into many different cell types. However, it remains unclear if all cell types are permissive to kolmiovirid replication. To focus on replication, we first bypassed the viral entry step and transfected established HDV, RDeV and SDeV replicons [9,13] into a battery of immortalized animal cell lines and followed viral replication over time by immunoblotting (Fig 1C–1G). We included cell lines derived from “natural hosts” including human, rodent (mouse and rat) and snake cell lines, but also from other mammalian species such as feline and simian.

All tested human cell lines (Huh7.5 –liver derived, HEK293T –kidney derived and A549 –lung derived) supported HDV and RDeV replication as shown by the accumulation of DAg over time (Fig 1C), with HDV replicating very poorly in the A549 cell line. Interestingly, SDAg accumulated to detectable levels in HEK293T and Huh7.5 cells, peaking at 4 days post-transfection (d.p.t) and decreasing at 6 d.p.t. (Fig 1C). Intriguingly, we were able to detect two forms of the SDAg, with distinct sizes, in the HEK293T and Huh7.5 cells transfected with the SDeV clone (Fig 1C). Rodent cell lines (NIH-3T3 –mouse fibroblasts and MCA-RH 7777 –rat liver derived) supported HDAg and RDAg but not SDAg accumulation (Fig 1D). The snake cell line I/1Ki (derived from *boa constrictor* kidney) supported RDAg and SDAg accumulation but was refractory to HDAg (Fig 1E). Interestingly, unlike in human HEK293T and Huh7.5 cells (Fig 1C), but in agreement with Hetzel *et al*. [7], only one form of the SDAg was detected in the I/1Ki cell line (Fig 1E). Two feline cell lines (FEA–cat embryonic fibroblasts and CRFK–kidney cortex derived) were able to support RDAg, but supported very poorly HDAg and SDAg accumulation (Fig 1F). Vero cells (African green monkey kidney derived) only supported RDAg accumulation (Fig 1G). In conclusion, our data show varying abilities of different cell lines to support HDAg, RDAg and SDAg expression. While all tested cell lines were permissive to RDAg expression, HDAg accumulated efficiently only in certain cell types (Fig 1C–1G). SDAg expression appeared to be the most restricted, as only the snake I/1Ki cell line supported efficient accumulation over time (Fig 1E).

**Fig 2:**
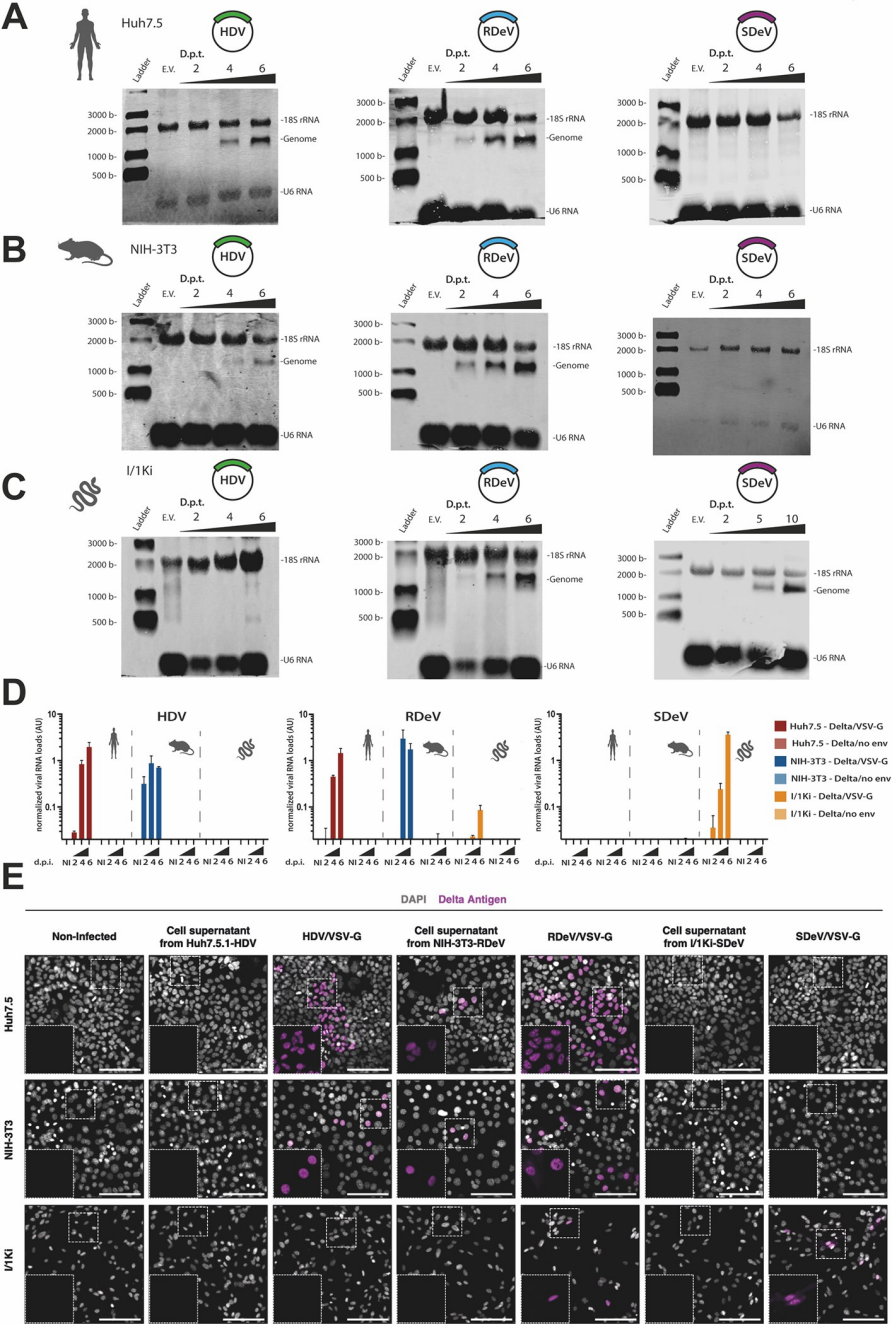
HDV, RDeV and SDeV RNA accumulation in immortalized cell lines following transfections and infections. (A-C) Deltavirus RNA accumulation in different immortalized cell lines. pcDNA3.1 plasmids encoding dimers of the human, rodent and snake deltavirus genome, or an empty backbone, were transfected in Huh7.5 (A), NIH-3T3 (B) and I/1Ki (C) cells. Cells were collected at either 2-, 4- and 6- or 2-, 5- and 10-days post-transfection (d.p.t.) and genomic RNAs were detected by Northern Blot. U6 and 18S ribosomal RNAs serve as loading controls. E.V.: Empty vector. D) RT-qPCR analysis of viral RNA in infected cell lines. Cells were collected and total extracted RNAs were used to synthesize cDNAs for qPCR analysis of the viral genomes. RNA levels were normalized using the level of the 18S ribosomal RNAs for each sample. Mean values and SD were calculated from 3 independent infections. dpi: days post-infection, AU: Arbitrary Units. NI: Non-infected E) Detection of the DAgs by immunofluorescence using the Ig-Patient1 serum in infected cell lines. Nuclei were stained using DAPI and representative images are shown, non-infected (N.I.) cells serve as negative controls. Delta Antigen is shown in magenta and nuclei in grey. Scale bar: 100 μm.

However, the accumulation of the delta antigens at short time points following transfection DNA plasmids is not a definitive measure of viral replication. Although sustained production of antigen (e.g., 6 days post-transfection) is most likely indicative of replication, expression at 2- or 4-days post-transfection (as detected in SDeV transfected HEK293T and Huh7.5) could be the result of the accumulation of delta antigen synthesis from mRNAs transcribed from the plasmid due to a putative cryptic promoter. To confirm that DAg accumulation reflects viral RNA replication, we extracted total RNA from Huh7.5, NIH-3T3 and I/1Ki cells, transfected with HDV, RDeV and SDeV replicons and performed Northern Blot analysis to detect viral genome increase over time (Fig 2A–2C). Our results show that while HDV genome was detected in Huh7.5 and NIH-3T3 cells, increasing over time (Fig 2A and 2B), the snake I/1Ki cells were unable to support HDV RNA accumulation (Fig 2C). The RDeV genome, on the other hand, was detected and increased over time in all three cell lines (Fig 2A–2C). Interestingly, the SDeV genome could only be detected accumulating in the I/1Ki snake cell line (Fig 2C), suggesting that the SDAg signal observed by Western Blot in human cell lines at two- and four-days post-transfection (Fig 1C) does not reflect viral genome replication but more likely SDAg expression from a cryptic promoter in the transfected SDeV plasmid replicon. Importantly, these results suggest that while RDeV RNA replication can occur in human, rodent and snake cells, SDeV RNA replication seems mostly restricted to snake cells.

We finally wanted to corroborate these results by initiating viral RNA replication through authentic viral infections. Indeed, HDV has previously been shown to produce infectious particles when packaged with Vesicular Stomatitis Virus (VSV)-G protein [32], suggesting that VSV-G may also be a suitable envelope glycoprotein for RDeV or SDeV. To produce VSV-G pseudotyped HDV, RDeV or SDeV infectious particles, we transfected either the previously described I/1Ki cell line persistently replicating SDeV [13], or in-house generated Huh7.5.1 and NIH-3T3 cell lines persistently replicating HDV and RDeV respectively (S3 Fig, see below), with a VSV-G encoding plasmid [43]. Supernatants from deltavirus replicating cells transfected or not with VSV-G were harvested and filtered to collect viral particles. HDV/VSV-G, RDeV/VSV-G and SDeV/VSV-G viral supernatants and non-transfected cell supernatant controls were used to infect Huh7.5, NIH-3T3 and I/1Ki cells. Cells were collected at 2-, 4- and 6-days post-infection (d.p.i.) for RT-qPCR and at 6 d.p.i. for immunofluorescence analysis. Both RT-qPCR (Fig 2D) and immunofluorescence (Fig 2E) experiments revealed that HDV, RDeV and SDeV could be packaged with VSV-G and form infectious viral particles, able to initiate viral replication in recipient cells. Indeed, RDeV/VSV-G could initiate replication in Huh7.5, NIH-3T3 and, to a lower extent in I/1Ki cells, with viral RNA genome levels increasing exponentially over time (Fig 2D and 2E). HDV/VSV-G was only able to initiate replication in the mammalian Huh7.5 and NIH-3T3 cells but not in the snake I/1Ki cells (Fig 2D and 2E), while SDeV/VSV-G could only efficiently replicate in the snake I/1Ki cell line (Fig 2D and 2E). Strikingly, we unexpectedly observed very low levels of infection in Huh7.5 and NIH-3T3 cells incubated with supernatants of RDeV replicating cells non transfected with VSV-G (Fig 2E). Although clearly inefficient, this points to the possibility that the RDeV RNP is constantly secreted in the media of replicating cells and able to enter recipient cells in a VSV-G-independent fashion. In conclusion, our experiments suggest that while RDeV can infect and replicate in a variety of cell lines, SDeV infection and replication appear restricted to snake cells while HDV infection is more adapted to mammalian cells.

### Effect of human ADAR-1 editing on RDAg production

During HDV replication, the synthesis of the S- and L-HDAgs is due to the translation of two distinct viral mRNAs [44]. An ADAR-1-dependent editing event of the HDV antigenomic RNA, transforms the amber stop codon (UAG) of the S-HDAg into a tryptophan (W) codon (UGG), extending the reading frame by 19 codons/AAs, thus allowing the transcription of a distinct mRNA coding for the L-HDAg [45–48]. The RDeV genome also possesses an amber stop codon that could be potentially edited into a W (UGG) in the end of the small RDAg (S-RDAg), giving rise to the putative larger RDAg (L-RDAg) form. We sought to investigate whether ADAR-1-knock-out (KO) or overexpression could affect RDAg accumulation.

To test this hypothesis, we knocked-out both ADAR-1 forms (p110 and p150) by CRISPR-Cas9 [49] (S2A Fig) or overexpressed both forms in two different human cell lines (S2A Fig) and verified L-DAg production. As expected, ADAR-1 KO cell lines showed compromised L-HDAg production, while ADAR-1 overexpression enhanced L-HDAg production (S2B Fig). Neither KO nor overexpression of ADAR-1 affected RDAg accumulation (S2B Fig), suggesting that ADAR-1-dependent editing of RDeV genome does not affect short-term RDAg accumulation in human cells.

### HDV, RDeV and SDeV RNA and DAg accumulation patterns in human, rodent and snake cells

More than a decade ago, several studies have shown that both HDAg and HDV RNAs localize to the nuclei of infected cells where viral replication takes place [28,50–55]. To characterize and compare HDV, RDeV and SDeV RNA and DAg localization patterns, we generated cells persistently replicating kolmiovirids: Huh7.5.1-HDV and NIH-3T3-RDeV (S3A Fig), and utilized a previously generated I/1Ki-SDeV cell line [13]. Briefly, Huh7.5.1 and NIH-3T3 cell lines were transfected with either HDV or RDeV replicons, alongside an mCitrine encoding plasmid. Fluorescence-activated cell sorting (FACS) served to select and sort transfected cells. Single clones were subsequently amplified and screened for deltavirus and Delta antigen presence by RT-qPCR and Western Blot respectively (S3A Fig). Huh7.5.1-HDV and NIH-3T3-RDeV clonal cell lines, along with a previously generated I/1Ki-SDeV cell line [13] were selected for this study and grown in the same conditions as their respective parental cell lines. All three cell lines were tested for the presence of HDV, RDeV and SDeV genomes and antigenomes by Northern Blot analysis (S3B Fig). Both genomic and antigenomic RNAs were detected in all three cell lines (S3B Fig), with genomes being more abundant than antigenomes (S3B Fig) as expected. These results confirm that viral RNA replication is indeed occurring in these cell lines (S3B Fig). The presence of plasmid DNA in these cell lines was assessed by PCR on genomic DNA (S3C Fig). While NIH-3T3-RDeV and I/1Ki-SDeV cell lines were free of plasmid DNA, residual DNA plasmid was detected in the Huh7.5.1-HDV cell line.

**Fig 3:**
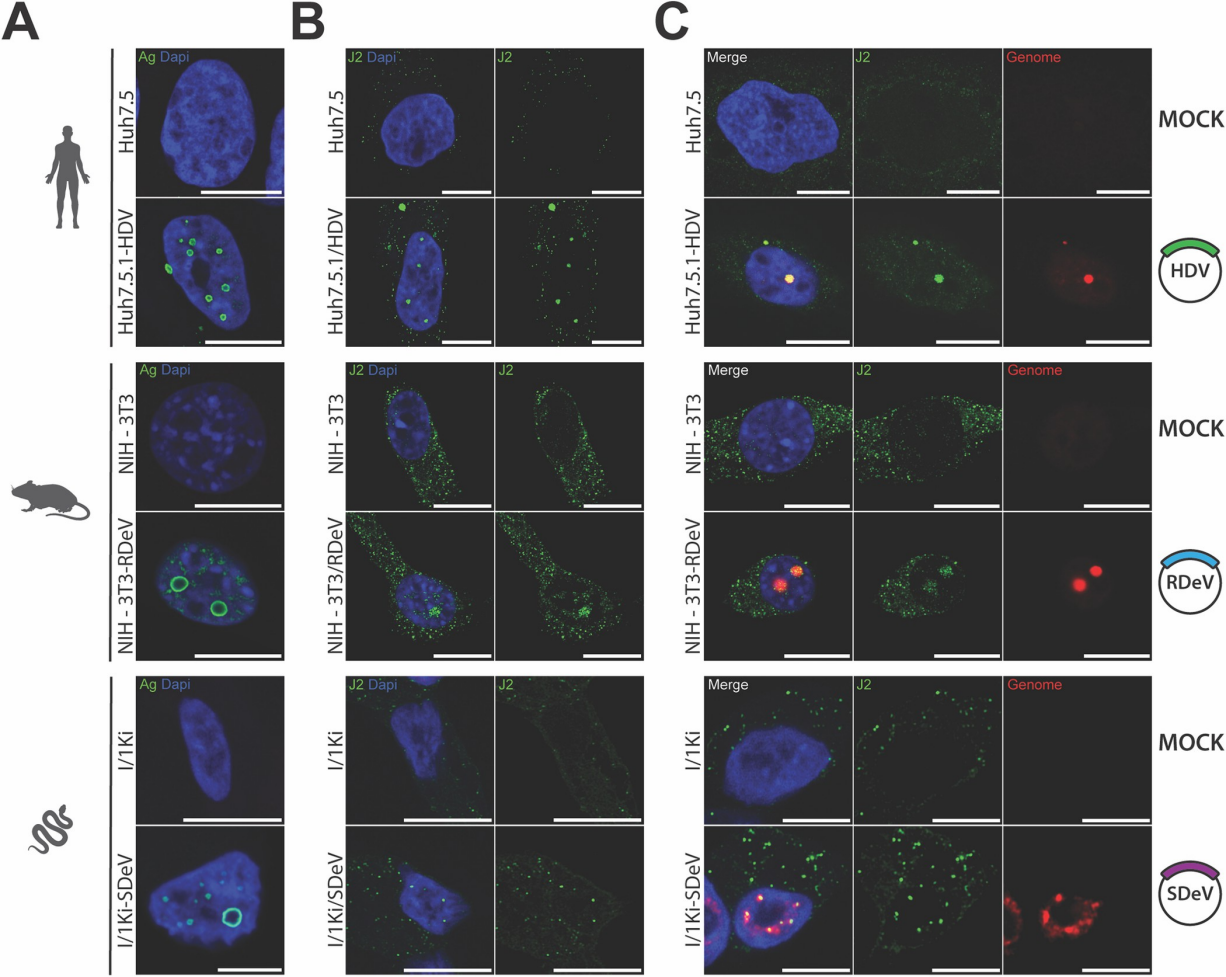
Kolmiovirid DAg and genome subcellular localizations. Cell lines persistently replicating HDV, RDeV and SDeV were plated on microscopy slides and fixed to visualize DAg and genome subcellular localization in Huh7.5.1-HDV, NIH-3T3-RDeV and I/1Ki-SDeV. The corresponding non-replicating cell lines served as negative controls. Nuclei (in blue) were stained using DAPI and representative confocal images are shown for A) Detection of the DAgs by immunofluorescence using the Ig-Patient1 serum (in green). B) Detection of double stranded RNA by immunofluorescence using the J2 antibody (in green). C) Co-detection of dsRNA by immunofluorescence using the J2 antibody (in green) and delta genomes by sm or smiFISH (in red). *Scale bars* 10 μm for Huh7.5, Huh7.5.1-HDV, NIH-3T3 and NIH-3T3-RDeV and 5 μm for I/1Ki and I/1Ki-SDeV. Cells were imaged on a LSM980 confocal microscope (Zeiss) and analyzed using ImageJ (version 2.9.0).

Immunofluorescence imaging of Huh7.5.1-HDV, NIH-3T3-RDeV and I/1Ki-SDeV cells, using IgGs purified from HDV-positive patient sera revealed a nuclear localization of HDAg, RDAg and SDAg in human, rodent and snake cells respectively (Fig 3A). We then investigated if the presence of HDV, RDeV and SDeV induces the formation of double-stranded RNA (dsRNA), a replication intermediate hallmark of viral infections [56]. J2 antibody staining, that specifically detects dsRNA, revealed that while some staining was present in the cytoplasm of control cells, a distinct nuclear signal was exclusively present in all cells persistently replicating kolmiovirids (Fig 3B). In order to ascertain if the detected J2 staining is a result of dsRNA originating from viral genomes, we implemented single molecule Fluorescence *In Situ* Hybridization (smFISH) and single molecule inexpensive FISH (smiFISH) [57] to specifically detect each viral genome. J2 and FISH co-staining revealed a clear nuclear co-localization of both signals in all cells persistently replicating kolmiovirids (Fig 3C). These data show that we can specifically stain viral genomes in cells persistently replicating kolmiovirids and that the dsRNA J2 staining is most likely originating from viral genomes, presumably either from replication intermediates or from the rod-like pseudo-double stranded structures formed by these viral RNAs.

**Fig 4:**
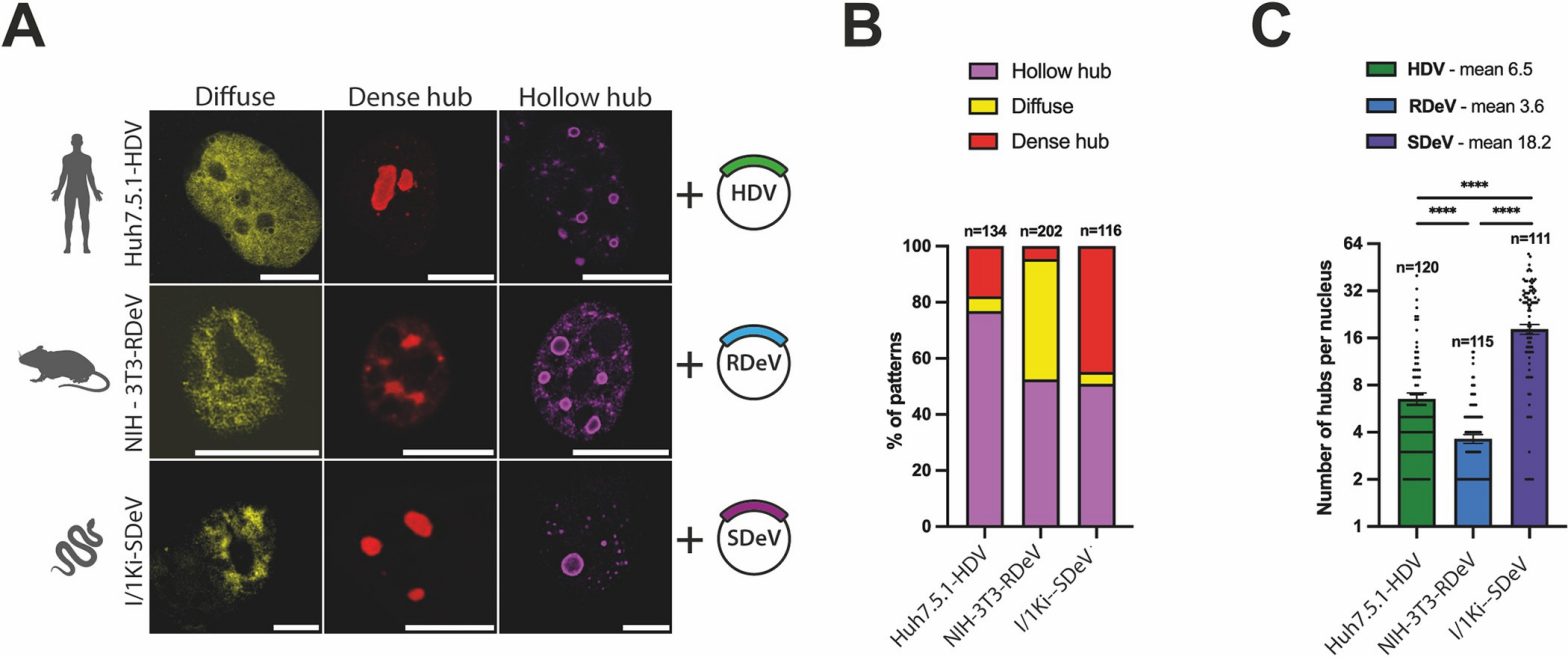
DAgs form different patterns in nuclei of cells persistently replicating kolmiovirids. A) Persistently replicating cells were plated on microscopy slides and fixed to visualize DAg structures in the nucleus. Representative confocal images are shown. DAgs (in yellow, red or magenta, depending on the pattern) were detected by immunofluorescence, *scale bars* 10 μm for Huh7.5.1-HDV and NIH-3T3-RDeV and 5 μm for I/1Ki-SDeV. Cells were imaged on a LSM980 confocal microscope (Zeiss) and analyzed using ImageJ (version 2.9.0). B) Quantification of the ratio between the three patterns described in panel A in Huh7.5.1-HDV, NIH-3T3-RDeV and I/1Ki-SDeV. Randomly selected fields of each cell line from at least 3 independent plating events were manually counted. C) Number of hubs (dense and hollow combined) in Huh7.5.1-HDV, NIH-3T3-RDeV and I/1Ki-SDeV. On the same randomly selected fields used for quantification in panel B, the number of hubs per cell was manually counted and plotted. Mean values and SEM (unpaired 2-side Student *t* test, with Welch’s correction, **** *P* < .0001).

Interestingly, although DAg staining was almost exclusively nuclear for all three viruses, we noticed a heterogeneity in the distribution pattern of viral proteins in nuclei of persistently replicating cells. We could classify the observed DAg distributions into three distinct patterns (Fig 4A), resembling what has previously been observed with HDV[53,54]: 1- a “diffuse” localization throughout the nucleus, 2- a concentrated signal in foci distributed equivalently throughout all Z-stacks that we termed “dense hubs” and 3- a concentrated signal in particular foci that form ring-like structures, devoid of staining in focal planes positioned in the middle of the foci, that we termed “hollow hubs” (Fig 4A). Quantification of these patterns in HDV, RDeV and SDeV stable cells revealed that the “diffuse” pattern is the least abundant in most cell lines (Fig 4B), while viral protein hubs are the most frequent, with the “hollow hub” pattern represented in at least half of all counted cells for all three viruses (Fig 4B). The number of observed hubs per cell varied greatly between the three viruses (Fig 4C), with RDeV stable cells containing the least (mean of ~4 hubs/cell) and SDeV stable cells containing the most (mean of ~18 hubs/cell) (Fig 4C).

**Fig 5:**
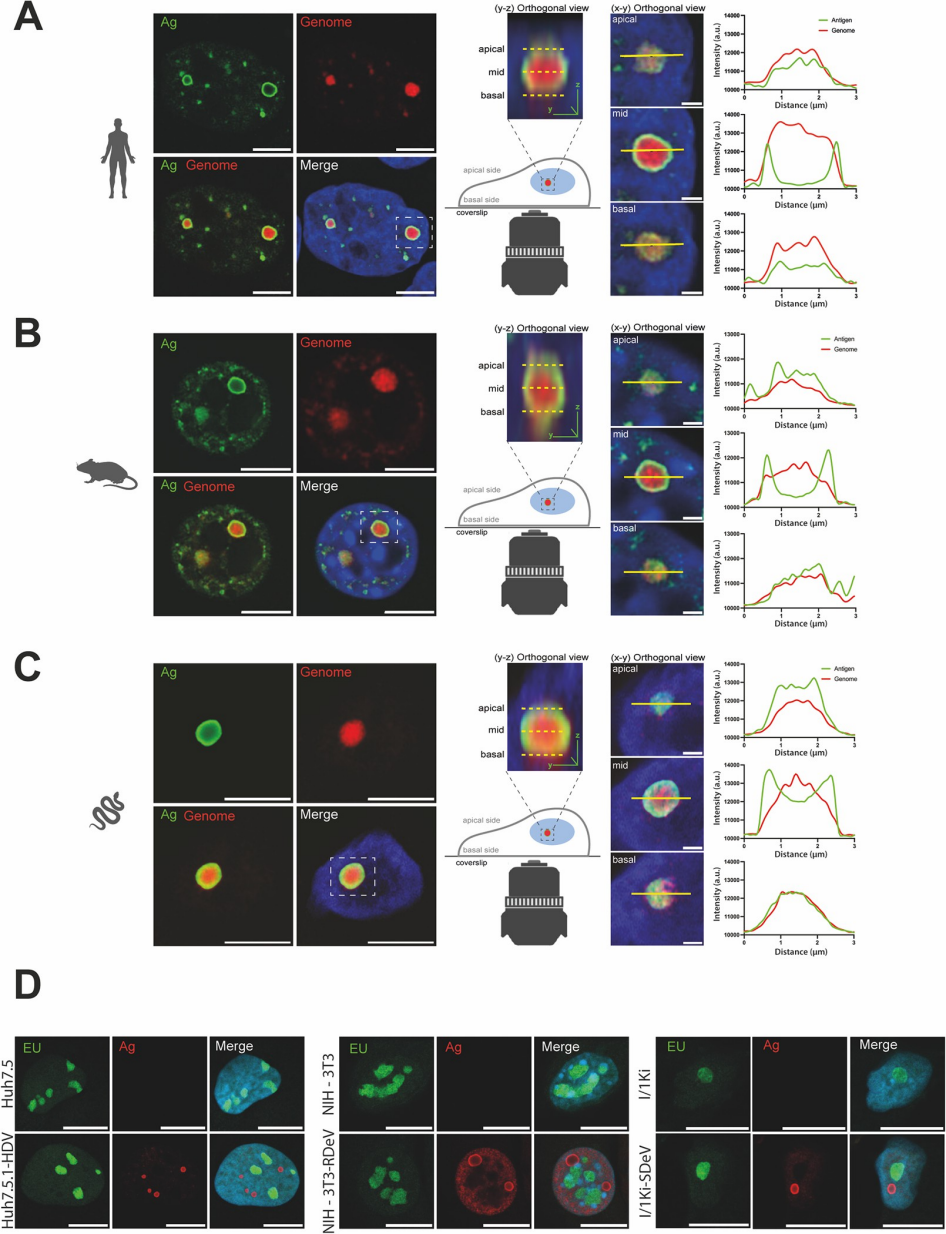
Viral hubs in cells persistently replicating kolmiovirids. Mock or persistently replicating Huh7.5.1-HDV (A), NIH-3T3-RDeV (B) and I/1Ki-SDeV (C) were plated on microscopy slides and fixed to visualize DAgs and RNA localization. Representative confocal images are shown. Orthogonal view (Y, Z) showing the organization of the hubs along the Z-axis are shown and used to define the basal, mid and apical planes of the presented hubs. Fluorescence intensity of the antigen and genome signals were determined along the X-axis at the basal, mid and apical planes of the hubs and plotted adjacent to the corresponding orthogonal views (X, Y). Nuclei (in blue) were stained using DAPI, DAgs (in green) were detected by IF staining and delta genomes (in red) were detected by sm or smiFISH, *scale bars* 10 μm for Huh7.5.1-HDV and NIH-3T3-RDeV confocal panels, 5 μm for I/1Ki-SDeV confocal panels and 1 μm for all X, Y orthogonal views. D) Nascent RNA staining (in green) in Huh7.5.1-HDV, NIH-3T3-RDeV and I/1Ki-SDeV. Nuclei (in blue) were stained using DAPI, nascent RNAs (in green) were detected using ethynyl uridine and DAgs (in red) were detected by immunofluorescence. Representative confocal images are shown. *Scale bars* 10 μm for Huh7.5.1-HDV and NIH-3T3-RDeV and 5 μm for I/1Ki-SDeV. Cells were imaged on a LSM980 confocal microscope (Zeiss) and analyzed using ImageJ (version 2.9.0).

Because the nuclear hollow hub was the most frequently observed pattern, we sought to determine the position of viral genomic RNA relative to DAg proteins in these structures using smFISH coupled to IF (smFISHIF) in these stable cell lines. Results from these experiments showed a very similar 3D structural organization for all three viruses. In fact, the aforementioned “hollow hubs” turned out to be packed with viral RNA (Fig 5). Indeed, imaging of these hubs along the Z-axis shows a peculiar 3D organization, where viral RNA is concentrated in the middle of the hubs, surrounded by viral protein staining (Fig 5). To confirm this organization, we quantified protein and RNA signals along the X-axis from images at different Z-stacks of representative “hollow hubs”. Two overlapping signals were detected at the apical and basal poles (Fig 5) whereas the middle of the hub shows a peak in RNA signal intensity surrounded by two antigen signal peaks (Fig 5). 3D reconstitutions of these hubs show spherical structures full of viral RNA surrounded by a “shell” of viral proteins (S1–S3 Videos).

To evaluate if the observed viral hubs are sites of active RNA transcription, we performed Ethynyl uridine (EU)-staining to label nascent RNA transcripts in cells persistently replicating kolmiovirids (Fig 5D). In all three cell lines we observed that the presence of viral hubs did not affect the general spatial organization of transcription, which remained concentrated in specific loci (presumably nucleolus) along with a diffuse signal across the rest of the nucleus. Importantly, no EU-staining could be detected in the RNA/Ag viral hubs, which were rather clearly depleted of nascent RNA signal in all three conditions. Furthermore, no clear enrichment of RNA Polymerase II (RNAPII), was observed in these viral hubs (S4 Fig), similar to what has been shown earlier with HDV [52], even though RNAPII is thought to be the main polymerase responsible for HDV RNA amplification[25,58]. Both our EU-labeling and RNAPII staining experiments, suggest that the observed viral RNA/Antigen hubs are probably not sites of highly active viral RNA transcription/replication, although live imaging of newly transcribed viral RNA (e.g. with the MS2 phage system), would be needed to completely rule out this hypothesis.

### HDV, RDeV and SDeV tail vein injections and viral replication *in vivo*

**Fig 6:**
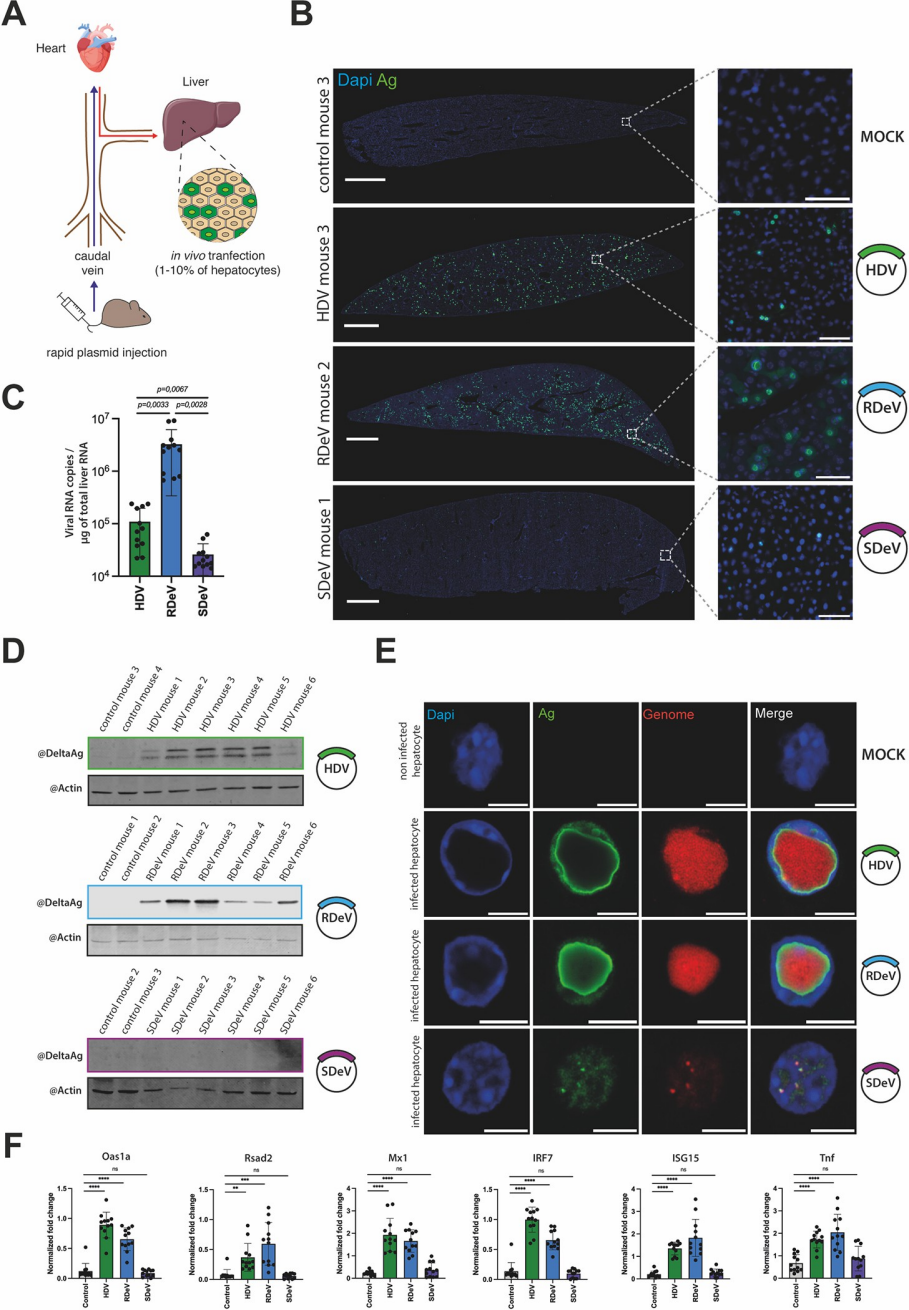
Deltavirus replication *in vivo*. Mice injected with empty pcDNA3.1 or pcDNA3.1 encoding dimers of the HDV, SDeV or RDeV genomes were euthanized and dissected 9 days post-injection. A) Schematic representation of hydrodynamic tail vein injections in mice. B) Immunofluorescence detection of the DAgs in mice liver sections. Liver samples were collected, sliced (10 μm) and fixed to visualize DAg expression in hepatocytes. Representative images are shown. Nuclei (in blue) were stained using DAPI and DAgs (in green) were stained by IF, *scale bar* 1 mm. The boxed areas are shown magnified, *scale bar* 50 μm. Slices were imaged on an Axioscan 7 (Zeiss) and analyzed using Zen Blue (Zeiss, version 3.7) and ImageJ (version 2.9.0). C) RT-qPCR analysis of viral RNA copy numbers in dissected mice liver. Liver samples were collected and total extracted RNAs were used to synthesize cDNAs for RT-qPCR analysis of the viral genomes. RNA levels were normalized using the level of the 18S ribosomal RNAs for each sample and viral RNA copy numbers were calculated using a serial viral plasmid dilution. Mean values and SD were calculated for 6 independent injections (i.e., 6 mice) with technical duplicates (n = 6 with technical duplicates for each, unpaired 2-side Student *t* test, with Welch’s correction, **** *P* < .0001). D) Western blot analysis of the DAgs’ expression in dissected mice livers. Liver samples were collected and protein extracts were analyzed by western blot for DAg and β-actin expression. E) smFISHIF detection of the DAgs and viral genomes in mice liver. Liver samples were collected, sliced (10 μm) and fixed to visualize DAg and genome localization in hepatocytes. Representative images are shown. Nuclei (in blue) were stained using DAPI, DAgs (in green) were detected by IF staining and delta genomes (in red) were detected by sm or smiFISH, *scale bar* 10 μm. Slices were imaged on a LSM980 confocal microscope (Zeiss). F) RT-qPCR analysis of ISG fold change expression in dissected mice liver. Liver samples were collected and total RNAs extracted and used to synthesize cDNAs for qPCR analysis of a selection of ISGs. RNA levels were normalized using the level of the 18S ribosomal RNAs for each sample. Mean values and SD were calculated for 6 independent injections (i.e., 6 mice) with technical duplicates (n = 6 with technical duplicates for each, unpaired 2-side Student *t* test, with Welch’s correction, ** *P* < .01, *** *P* < .001, **** *P* < .0001, ns: not statistically significant.).

The development of a mouse model to study *Kolmioviridae* replication and associated pathogenesis would have obvious benefits. Chang *et al*. [59] were able to initiate HDV replication in mouse hepatocytes *in vivo* by hydrodynamic tail vein injection (HDTV) of plasmid DNA harboring HDV genome [59] (Fig 6A). We applied this method to verify if RDeV and SDeV, similarly to HDV, can replicate in hepatocytes *in vivo*.

We performed HDTV on 24 C57BL/6J female mice in total, with plasmids harboring either HDV (n = 6), RDeV (n = 6) or SDeV (n = 6) genomes, or empty pcDNA3.1 as mock control (n = 6) (Fig 6A). We sacrificed the animals 9 days post-injection and collected liver samples. IF staining of DAgs on liver sections showed no signal in mock injected mice, a very weak signal in SDeV injected animals and a clear nuclear signal in hepatocytes of HDV and RDeV injected mice (Fig 6B). These results are in agreement with viral RNA quantification determined by RT-qPCR (Fig 6C). Indeed, while RDeV RNA was the most abundant, HDV RNA was ~1.5 log lower and SDeV RNA was 2 logs less abundant than RDeV RNA (Fig 6C). Western blot analysis detected both small and large HDAgs as well as RDAg in all injected HDV and RDeV mice, however SDAg was undetectable in animals injected with the SDeV plasmid (Fig 6D). Altogether, these results show that similarly to HDV [59], RDeV can efficiently replicate in mouse hepatocytes, reaching even higher levels than HDV (Fig 6C). SDeV, on the other hand, does not replicate in mouse liver (Fig 6B–6D), consistent with our *in vitro* data (Fig 1D).

Strikingly, our IF results on stained liver slices of RDeV and HDV injected mice showed massive hubs with unusual nuclear DAg staining (Fig 6B). Using smFISHIF and confocal microscopy to take a closer look at these hubs, we observed that both HDV and RDeV positive nuclei accumulated large amounts of viral RNAs, forming massive hubs of RNA surrounded by DAg staining (Fig 6E), reminiscent in their 3D organization, to the hubs observed in culture models (Fig 5) albeit much larger in size (Fig 6E, S4–S6 Videos). Remarkably, in the majority of HDV and RDeV positive cells, viral hubs occupied up to ~75% of the host cell’s nuclear volume, displacing the DNA counterstain by DAPI to nuclear borders (Fig 6E, S4–S6 Videos). Interestingly, in the very few SDeV positive cells that we were able to find, the smFISHIF staining revealed much smaller RNP foci (Fig 6E, S6 Video).

Finally, we wished to verify if *Kolmioviridae* replication via HDTV could recapitulate immune gene induction observed with HDV in several infection models [60–63]. RT-qPCR probing a selection of interferon-stimulated genes (ISGs) and tumor necrosis factor alpha (Tnfα) mRNAs were performed on RNA extracted from livers from different mouse groups (Fig 6F). Mx1, Oas1a, Rsad2, ISG15, IRF7 and Tnfα mRNAs were all upregulated upon HDV and RDeV injection, however, no induction was observed after SDeV injection, suggesting that the immune gene induction is likely the result of kolmiovirid replication.

## Discussion

The discovery of HDV-*like* sequences in duck cloacal samples and in various boa constrictor organs, prompted several laboratories to examine and identify similar sequences across a wide range of taxa [6–12,15]. The findings led to the creation of the novel realm *Ribozyviria* with the family *Kolmioviridae* to host the genus *Deltavirus* and seven other genera [16]. As only a minority of the studies on novel kolmiovirids made efforts towards molecular characterization of their replication [9,11,14], our understanding of the kolmiovirid biology relies on the seminal research performed on HDV. Importantly, the cross-species transmission potential of kolmiovirids [10] coupled to their ability to form infectious particles with envelope proteins of a variety of helper viruses [13,32] raises an important question: can these minimal RNA viruses replicate in any animal cell they access? Here, by comparing molecular replication hallmarks of two recently identified kolmiovirids, RDeV and SDeV, to HDV, we reveal differential and convergent features governing their interaction with their host, and provide estimations of their host range, and thus host-shifting potential [10].

To allow unbiased comparison, we constructed replicons for HDV, RDeV, and SDeV in the same orientation and expression vectors and studied their replication in nine different animal cell lines. We show that while RDeV replicated in all tested cell lines, HDV replicated in the majority of the mammalian cell lines and SDeV replicated only efficiently in snake cells (Figs 1C–1G and 2). This indicates that while RDeV appears to be a generalist, host specific factors may control SDeV and HDV replication. In the future, determining sequences in the RDeV genome (e.g., antigen, promoter sequences, ribozymes) conferring this virus its “super-replicator” status would shed light on important aspects of kolmiovirid replication.

The ability of RDeV to replicate in cell lines from highly divergent species was confirmed in RDeV/VSV-G infection experiments (Fig 2). By generating VSV-G pseudotyped HDV, RDeV and SDeV infectious viral particles we could initiate kolmiovirid replication through ‘authentic’ viral infections, in a DNA-independent manner (Fig 2D and 2E). This revealed the ability of RDeV/VSV-G to infect not only rodent cells but also human and, to a lesser extent, snake cells (Fig 2D and 2E), suggesting that the envelope with which RDeV is packaged could be an important limiting factor dictating RDeV host range and tropism. Strikingly, we observed low levels of RDeV infection when using supernatants from delta replicating cells (NIH-3T3-RDeV) not transfected with VSV-G (Fig 2D and 2E). This indicates that RDeV RNP could be secreted in the media of delta replicating cells and could enter target cells in a VSV-G-independent manner. We speculate that this infection route could either be mediated by endocytosis of naked delta RNP, by the exosome pathway or by the delta RNP association with an endogenous glycoprotein present in producing cells, but this will require further investigation.

The specific packaging step of deltavirus RNPs with glycoproteins from other viruses, is most likely divergent between HDV and animal deltaviruses. Indeed, because neither SDeV nor RDeV appear to produce a large DAg (Figs 1 and S3, 7,9], important for packaging with HBV surface (S) protein [64,65], it is tempting to speculate that co-evolution with HBV, particularly HBsAg, made ADAR-1 editing useful for HDV that co-opted its function. The ADAR-1 editing allows for the prenylation of cysteine 211 on the L-HDAg, that anchors it to cellular membranes, facilitating its interaction with HBsAg and the assembly of HDV infectious particles [30,64–66].

Our study suggests that, in addition to ADAR-1 editing, divergent host factor dependencies and specific viral sequences likely contribute to kolmiovirid’s host-specificity. Our data calls for more systematic studies addressing the host-shifting potential of kolmiovirids and assessing which virus would potentially be more prone to zoonoses.

Importantly, our results indicate that one should be cautious when interpreting results from Western blots at early time points following DNA transfection experiments to initiate viral replication. Indeed, while SDeV was clearly unable to initiate RNA replication in human cells (Fig 2), our Western blot results (Fig 1C) were somehow misleading. In fact, a protein signal was detected in human cells after SDeV replicon plasmid transfection (Fig 1C), probably due to a cryptic DNA promoter, which was also observed by other studies [13].

The analysis of kolmiovirid replication in persistently replicating cells through IF revealed three DAg staining patterns similar for all viruses. Simultaneous viral RNA and DAg detection of the major hollow hub pattern through smFISHIF followed by high-resolution microscopy, revealed that the observed hubs are in fact full of a viral RNA. Cells persistently replicating kolmiovirids showed a peculiar RNA-protein structure, formed by RNA condensates surrounded by a layer of DAg. This organization seems to be a defining feature of kolmiovirid accumulation in nuclei, as it was also observed *in vivo* in transfected mice liver (Fig 6E) as well as in cells infected with VSV-G packaged Deltaviruses (Fig 2E). Interestingly, RNA-protein hubs observed in mouse hepatocytes were often massive, occupying the majority of positive nuclei. One hypothesis explaining the difference between the observed patterns and the size of different hubs relates to cell cycle progression. Specifically, the hubs would be smaller in size and in higher numbers in immortalized cell cultures, because of their dilution after each cell division. In contrast, continuous viral replication in non-dividing mouse hepatocytes would cause these hubs to grow and presumably coalesce, reaching very large sizes. Admittingly, the function of these hubs during the viral lifecycle is still unknown, but we observe that they are transcriptionally inactive, as no nascent RNA could be detected in these structures (Fig 5) and co-staining for viral Ag and RNAPII did not reveal an enrichment of RNAPII in these viral hubs (S4 Fig). Interestingly, it has previously been reported that HDAg/RNA foci are transcriptionally inactive yet are closely located, but do not co-localize, with components of the nuclear speckles, SCR5 and RNAPII [50,76]. Further characterization of these hubs is needed but our initial data suggest that these hubs constitute a conserved feature of kolmiovirid accumulation in host cells. Investigating whether these hubs are viral storage or assembly sites should be addressed in future studies. Importantly, it must be made clear that no envelope glycoproteins were co-expressed in the stable cell lines used to characterize these hubs. Whether the presence of viral glycoproteins (HBsAg, VSV-G or others) could affect the organization, stability and dynamics of these hubs, remains to be determined. Curiously, the organization of these structures is reminiscent of liquid-liquid phase separations (LLPS) observed in membraneless organelles, such as the nucleolus [67] or several viral inclusion bodies (IB) [68,69]. Indeed, many viruses, including negative-strand RNA viruses [70], such as Rabies virus (RABV) [71], VSV [72], Measles virus (MeV)[73] and influenza A virus (IAV) [74] are known to form LLPS in the cytoplasm of infected cells to promote viral replication or genome assembly [75]. Interestingly, a common feature of these virus-induced LLPS is that their formation requires, apart from an RNA component, proteins containing RNA binding motifs, oligomerization domains and domains with intrinsically disordered regions (IDRs) [75], which are all present in DAgs (Fig 1A). In the future, tracking the morphology of the observed kolmiovirid hubs by live microscopy approaches (e.g. ability to fuse, fluorescence recovery after photobleaching), would determine if the observed hubs have liquid- or gel-like properties [76].

Finally, our HDTV experiments show that unlike RDeV and HDV, SDeV is not able to efficiently replicate in mouse hepatocytes *in vivo*, in agreement to what is observed in cell culture. This suggests that the observed differences likely reflect the ability of RDeV and HDV, but not SDeV, to initiate replication in mice, which would subsequently dictate the induction of ISGs [60,63,77] (Fig 6). In conclusion, our results suggest differences in the cross-species transmission potential between kolmiovirids. Importantly, amongst viruses evaluated in our study, RDeV appears to be omnipotent in initiating replication in cells of various animal species. Given this ability, the modalities of RDeV transmission *in vivo* are of major interest. Because the virus was initially identified by RNA-seq [9], we have no information on how the virus is transmitted *in vivo*. Future HDTVI of RDeV replicon in mice, allowing it to replicate in mouse hepatocytes for longer periods of time, would determine if the virus is able to spread *in vivo* in the absence of any helper virus.

## Methods

### Sequence analyses and computational protein structure prediction

The multiple protein sequence alignment shown in Fig 1A was computed by Clustal Omega on the EMBL-EBI server. Computational meta-disorder predictions and consensus secondary structure prediction were obtained from the Dismeta webserver [78]. Conservation scores based on alignment of sequences from HDAg (genotype 1 –HDV isolate Taylor), Tome’s spiny rat virus 1 (TSRV-1) delta antigen from isolate 0180 (rodent DAg) and Swiss snake colony virus 1 (SwSCV-1) delta antigen from isolate F18-5 (snake DAg) were calculated using AL2CO [79] with a 10-residue sliding average, as implemented in Chimera [80]. Structure predictions of HDAg, RDAg and SDAg were performed using a SBGrid consortium installation of AlphaFold multimer version 2.3 running on a local server equipped with a NVIDIA Tesla A100 GPU [81,82]. The full databases were used, with max_template_date = 2022-12-22. All other parameters were left to their default values.

### Plasmids and cloning

pcDNA3.1 plasmids encoding dimers of the human (Genbank accession number M21012.1) and rodent (Genbank accession number: MK598004) deltavirus genomes where previously described [9]. pcDNA3.1 plasmid encoding a dimer of snake deltavirus (Genbank accession number MH988742) was generated by PCR amplification of the snake deltavirus dimer insert from pCAGGS-2xSDeV-fwd [13] and cloned into a EcoRV/XbaI linearized pcDNA3.1. Clones were sequenced and plasmid in the forward orientation was selected and amplified for further use. pLentiCMVPuroDEST plasmids (Addgene #17452) encoding the FLAG tagged human, rodent, avian, toad and snake delta (SDAg-S and SDAg-L) virus antigens were generated by PCR amplification of the antigen coding sequences with primers listed in S1 Table, from pCAGGS 1.2x plasmids of HDV, RDeV, SDeV, avian and toad deltaviruses described in Szirovicza *et al*.[14]. The amplified fragments were then cloned into pLentiCMVPuroDEST (Addgene #17452) plasmids by Gibson assembly (New England Biolabs #E2611L). The ADAR-1 p110 and ADAR-1 p150 coding sequences were amplified by PCR from pcDNA3.1 cDNA plasmids kindly provided by Dr. Jean-Pierre Vartanian and cloned into the pLentiCMVPuroDEST vector (Addgene #17452) using Gibson assembly. All primers used for cloning are listed in S1 Table.

### Cell culture

Huh7.5 (cancerous Human hepatocytes, RRID:CVCL_7927), HEK293T (transformed human embryonic kidney cells RRID:CVCL_0063), A549, NIH-3T3 (spontaneously immortalized NIH Swiss mouse embryonic fibroblasts, RRID:CVCL_0594), MCA-RH 7777 (cancerous buffalo rat hepatocytes, RRID:CVCL_0444), FEA (spontaneously immortalized cat embryonic fibroblasts, RRID:CVCL_UG17), CRFK (spontaneously immortalized cat kidney epithelial cells, RRID:CVCL_2426), and Vero (spontaneously immortalized green monkey kidney epithelium, RRID:CVCL_0059) cells were maintained in Dulbecco’s modified Eagle’s medium (DMEM; Gibco #41965–039) supplemented with 10% heat-inactivated fetal calf serum (Sigma-Aldrich #F75424), and 100 μg/mL of normocin (InvivoGen #ant-nr-2) and grown in cell culture flasks at 37°C in a humidified incubator containing 5% CO2. I/1Ki (*boa constrictor* kidney cells)[83] and I/1Ki-Δ (referred to as I/1Ki-SDeV in this paper) [13] were maintained in Minimal Essential Medium Eagle (MEM; Gibco #31095–029) supplemented with 20% heat-inactivated fetal calf serum (Sigma-Aldrich #F75424), and 100 μg/mL of normocin (InvivoGen #ant-nr-2), and grown in cell culture flasks at 30°C in a humidified incubator containing 5% CO2. All cell lines used in the study were mycoplasma-free and were checked regularly for mycoplasma contamination.

### Transfections

Huh7.5, HEK293T, A549, NIH-3T3, MCA-RH 7777, FEA, CRFK, and Vero cells were transfected with Jet Pei reagent (Polyplus #101000020) using 1 μg of plasmid DNA and according to the manufacturer’s instructions. I/1Ki cells were transfected with Lipofectamine 3000 (Invitrogen #L3000008) using the reverse transfection method. 1.5 μL Lipofectamine 3000 was diluted in 25 μL of Opti-MEM Medium (Gibco #31985–062), and 1 μg of plasmid DNA was diluted with 2 μL P3000 reagent in 25 μL of Opti-MEM. Both solutions were mixed, incubated for 15min at room temperature (RT) and added to 1.5x10^5^ cells suspended in 1 mL of culture medium. The cells were kept in suspension with the transfection mix for 15 to 30 minutes RT before being seeded in 24-well plates. Transfection medium was replaced for culture medium 6h post-seeding.

### Establishment of HDV and RDeV expressing cell lines

To establish an Huh7.5.1 cell line persistently replicating HDV and a NIH-3T3 cell line persistently replicating RDeV both cell lines were co-transfected with an mCitrine encoding plasmid kindly provided by Dr. E Kremer and a pcDNA3.1 plasmid encoding a dimer kolmiovirid coding sequence in a 1:4 ratio, HDV genotype 1 (Taylor isolate—GenBank accession number M21012.1) and RDeV isolate 0183 (GenBank accession number MK598004) respectively. Two days post-transfection single clones were sorted into 96-well plates containing conditioned medium by Fluorescence-Activated Cell Sorting (FACS). Cell clones were amplified, and screened for the presence of HDV or RDeV genomes and antigens by RT-qPCR and western blot. Clones were selected for this study and grown in the same conditions as their respective parental cell lines.

### Establishment of ADAR-1 KO cell lines

To generate ADAR-1 p110 and p150 KO cell lines, the following primers corresponding to guide RNAs targeting early exons of ADAR-1 were cloned into the *Sp*Cas9-expressing lentiviral vector lentiCRISPRv2[49]: ADAR-1 p110 and p150 Fwd: 5’- CACCGAATTGACATGGAAAGGCAGG-3’; and Rev: 5’- AAACCCTGCCTTTCCATGTCAATTC-3’; and ADAR-1 p150 Fwd: 5’ CACCGAATTGACATGGAAAGGCAGG- 3’; and Rev: 5’- AAACCCTGCCTTTCCATGTCAATTC- 3’. Lentiviral particles pseudotyped with the VSV-G protein were produced by co-transfecting HEK293T cells in 6-well plates with 3 μg lentiCRISPRv2 vector [49], 1.5 μg VSV-G Env expression vector pMD2.G (Addgene #12259) and 1.5 μg Gag-Pol expression vector psPAX2 (Addgene #12260) vector using a standard calcium chloride transfection protocol. Viral supernatants were harvested 48 h after transfection, filtered (0.45 μm), and stored at −80°C or used directly for transduction. Huh7.5 and HEK293T transduced cell lines were selected in 1 μg/mL or 3.5 μg/mL of puromycin respectively for at least 5 days prior to use in assays. Thereafter, protein lysates were collected from the transduced cells and protein levels of the ADAR-1 p110 and p150 were assessed by immunoblotting.

### Establishment of ADAR-1 overexpressing cell lines

To generate ADAR-1 p110 and p150 overexpressing cell lines, lentiviral particles pseudotyped with the VSV-G protein were produced by co-transfecting HEK293T cells in 10cm dishes with 5 μg pLentiCMVPuroDEST vector (Addgene #17452), 2 μg VSV-G Env expression vector pMD2.G (Addgene #12259) and 2 μg Gag-Pol expression vector psPAX2 (Addgene #12260) using Jet PEI reagent (Polyplus #101000020) according to the manufacturer’s instructions. Viral supernatants were harvested 48 h after transfection, filtered (0.45 μm), and stored at −80°C or used directly for transduction. Huh7.5 and HEK293T transduced cell lines were selected in 1 μg/mL or 3.5 μg/mL of puromycin respectively for at least 5 days prior to use in assays. Thereafter, protein lysates were collected from the transduced cells and protein levels of the ADAR-1 p110 and p150 were assessed by immunoblotting.

### Animal models

All reported animal procedures were carried out in accordance with the rules of the French Institutional Animal Care and Use Committee and European Community Council (2010/63/EU). Animal studies were approved by institutional ethical committee (Comité d’éthique en expérimentation animale Languedoc-Roussillon (#36)) and by the Ministère de l’Enseignement Supérieur, de la Recherche et de l’Innovation (Apafis #40209–2023010417589371 v3). Hydrodynamic tail vein injections were performed in 6 to 8 week-old C57Bl/6J female mice (Janvier), as previously described [84]. Briefly, 0.1 mL/g body weight of a solution of sterile saline containing plasmids of interest were injected into the lateral tail vein over 8-10s. HDV / SDeV / RDeV, or empty pcDNA3.1 as control (12.5 μg) were injected together with pSBbi-RN (Addgene #60519) integrating reporter plasmid encoding dTomato (12.5 μg) and sleeping beauty transposase SB100X (Addgene #34879) (2.5 μg). pSBbi-RN (Addgene #60519) and pCMV (CAT)T7-SB100 (Addgene #34879) were gifts from Drs. Eric Kowartz and Zsuzsanna Izsvak, respectively. At 8–9 days post-injection, mice were fasted for 4–6 hours then sacrificed by anesthetic overdose with isoflurane. Livers were collected and cryopreserved in tissue freezing medium (Microm microtech #F/TFM-C) in liquid Nitrogen cooled isopentane following classical procedure. RNA and proteins were extracted from snap frozen samples of the liver caudate lobe.

### Antibody isolation from patient serum

Antibody targeting Hepatitis delta antigen (HDAg) were purified from serum of a cohort of HBV/HDV coinfected patients (n = 6) [85] using the MabTrap Kit (Cytvia #17112801) according to the manufacturer’s instructions. The antibody used for all subsequent DAgs stainings and western blot experiments, Ig-Patient1, was described earlier [41,42]. Human serum from patients with chronic HBV/HDV infection followed at the Strasbourg University Hospitals, Strasbourg, France was obtained with informed verbal consent. Protocols were approved by the local Ethics Committee of the Strasbourg University Hospitals (CPP) and the Ministry of Higher Education and Research of France (DC 2016 2616).

### Western blot

Cells were washed with PBS and lysed with 1X RIPA buffer (Merck #20–188) supplemented with a protease inhibitor cocktail (Thermo Scientific #87785). Total protein samples were denatured in 1X Laemmli buffer (Bio-Rad #1610747) supplemented with 10% β-mercaptoethanol (Bio-Rad #1610710) for 5 min at 95°C, and loaded on 4–20% Mini-PROTEAN TGX gels (Bio-Rad #4561093). Electrophoresis was performed at 120V for 1 hour. Proteins were subsequently transferred onto PVDF membranes (Bio-Rad #10026933) for 7 min at 2.5A and 12V using a Trans-Blot Turbo Transfer System (Bio-Rad #1704150EDU). Membranes were saturated in PBS (137 mM NaCl, 2.7 mM KCl, 10 mM Na_2_HPO_4_, 1.8 mM KH_2_PO_4_) 0.1% Tween 20 (Bio-Rad #1610781) (PBST) containing 5% dry milk (Régilait #731142) for 30 min at RT. Specific primary antibodies were incubated overnight at 4°C in PBST containing 2% dehydrated milk. Membranes were washed 3 times in PBST at RT and iRDye labeled specific secondary antibodies were incubated for 1 hour at RT in PBST in the dark. Membranes were washed 3 times in PBST at RT and iRDye labeled specific secondary antibodies were detected using the Odyssey M Infrared Imaging System (LI-COR Biosciences #3350). Proteins from liver tissues were extracted from freshly dissected mouse liver tissue fragments, collected in 2 mL tubes, flash frozen in liquid nitrogen and treated with a lysis buffer (150mM NaCl, 50mM Tris pH7.5, 1% Triton X-100 (Bio-Rad #1610407), 1% SDS) supplemented with a protease and phosphatase inhibitor cocktail (Thermo Scientific #78430). The protein concentration of each lysate was measured using the Pierce BCA Protein Assay Kit (Thermo Scientific #23227) and 20 μg of protein from each sample were denatured and treated as described above.

Mouse anti β-actin monoclonal antibodies (Invitrogen, MA5-11869) diluted 1:2,000 were used to detect β-actin protein as a loading control. Polyclonal antibodies extracted from patient sera (Ig-Patient1 for all blots except HDV and SDeV antigens detected in mice samples which were detected with Ig-Patient5) were used to detect the HDAg and RDAg. Rabbit serum/antiserum immunized with recombinant SDAg[7] diluted 1:2,000 were used to detect SDAg. Rabbit anti-ADAR-1 monoclonal antibodies (Cell Signaling Technology D7E2M #14175) and polyclonal antibodies (ThermoFisher Scientific #A303-883A-T) diluted 1:2,000 were simultaneously used to detect ADAR-1 p110 and p150. iRDye 800CW-conjugated goat anti human IgG secondary antibodies (LI-COR Biosciences #926–32232), iRDye 680RD-conjugated donkey anti-mouse IgG secondary antibodies (LI-COR Biosciences #926–68072) and iRDye 800CW-conjugated donkey anti-rabbit IgG secondary antibodies (LI-COR Biosciences #926–32213) diluted 1:10,000 were used to detect human sera, mouse β-actin antibodies, rabbit ADAR-1 antibodies and rabbit SDAg anti-serum, respectively.

### RNA isolation and Northern Blots

For genome and antigenome detection in cells persistently replicating kolmiovirids, cells were grown in T175 culture flasks (Falcon #353136), washed in PBS, trypsinized, pelleted and collected in 2 mL TRIzol reagent (Invitrogen #15596018). Samples were homogenized (vortex) with 400 μL Chloroform (Carlo Erba Reagents 438601), incubated at RT for 5 min and centrifuged for 15 min at 12.000 g at 4°C. Clear fractions were mixed with equal volumes of ethanol and transferred to Zymo-Spin IICR Columns (Zymo Research #C1078). RNA purifications were carried out as instructed in the Direct-zol RNA miniprep kit (Zymo Research, #R2050).

Genome detection in transfected cells were performed from 2 6-well plate wells per condition, transfected as previously described with 1 μg plasmid DNA. Cells were washed in PBS and collected in 1 mL TRIzol reagent (500 μL per well). Samples were homogenized (vortex) with 200 μL Chloroform, incubated at RT for 5 min and centrifuged for 15 min at 12.000 g at 4°C. Clear fractions were homogenized (vortex) with equal volumes of isopropanol (Honeywell #1219), incubated for 5 min at 4°C and centrifuged for 15 min at 12.000 g at 4°C. RNA pellets were washed with 75% Ethanol, air-dried and resuspended in 25 μL ultrapure water.

Total RNA samples were quantified using a NanoVolume N50 NanoPhotometer (Implen) and the indicated amounts were denatured in an equal volume of Gel Loading Buffer II (95% Formamide, 18 mM EDTA, 0.025% SDS) with a 2x final concentration of SybrGold (Thermo Fisher Scientific #S11494) for 15 min at 55°C. Samples were loaded, along with 1 μL ssRNA ladder (New England Biolabs #N0362S, also denatured) onto 1% agarose, 1X MOPS (Lonza #50876), 6.66% formaldehyde (Thermo Scientific #A16163) denaturing gels. Electrophoresis was performed at 100V for 1 hour. Total RNA was then visualized in the gel using a UV transilluminator, transferred onto Hybond-N^+^ nylon membrane (Amersham #RPN203B) and crosslinked using an ultraviolet cross-linker (120 mJ/cm^2^ at 254 nm). Membranes were pre-hybridized at 37°C for 30 min in 5 mL of PerfectHyb Plus Hybridization Buffer (Sigma-Aldrich #7033). Labeled DNA probes were then added to the PerfectHyb Plus buffer for overnight hybridization at 37°C. When smiFISH probes were used, 10 μL FLAP-X or -Y containing smiFISH probes previously duplexed (as described in the smFISH section) with Cy3 conjugated FLAP-X or -Y probes were used. Membranes were washed once in 0.1% SDS, 2x Saline-sodium citrate (SSC) at RT and once in 0.1% SDS, 1x SSC at RT. Membranes were imaged using the Odyssey M Infrared Imaging System (LI-COR Biosciences #3350).

### DNA isolation and polymerase chain reactions (PCR)

Total DNA from Huh7.5, Huh7.5.1-HDV, NIH-3T3, NIH-3T3-RDeV, I/1Ki and I/1Ki-SDeV cells were extracted using the InstaGene Matrix kit (Bio-Rad #7326030) according to the manufacturer’s instructions. PCR was performed using GoTaq G2 Flexi DNA Polymerase (Promega #M7805). For all amplifications, 50 μL of PCR mixtures were prepared as follows: 1x GoTaq Green Flexi Buffer, 2.5 mM of MgCl_2_ solution, 0.2 mM of each dNTP, 0.5 μM of each primer, 1.25 U of GoTaq G2 Flexi DNA Polymerase, 10 μl of DNA template or 5 ng of viral vector and ddH_2_O. Touchdown PCR amplifications were performed as follows: 98°C for 2 min, 10x (10 s at 98°C, 15 s at 65°C and 30 s at 72°C), decreasing the annealing temperature by 1°C per cycle and samples were amplified 25x (10 s at 98°C, 10s at 58°C and 30s at 72°C), 2 min at 72°C. PCR products were subjected to gel electrophoresis using 1% agarose gel. The amplicons were compared to the GeneRuler 100 bp DNA Ladder (Thermo Scientific #SM0241). All primers used are listed in S1 Table.

### VSV-G pseudotyped Deltavirus particle production and infection

For pseudotyped Deltavirus particle production, 42 cm^2^ of Huh7.5.1-HDV, NIH-3T3-RDeV or I/1Ki-SDeV cells were reverse transfected with Lipofectamine 3000 (Thermo Scientific #L3000008) in 10 cm dishes with 10 μg of VSV-G (pMD.G) [43]. Two days later the supernatants were harvested, passed through 0.45 μm filters (Clearline #146561) and used directly for infection experiments. VSV-G-pseudotyped viral productions were titered using the TCID50 technique and titers were calculated as FFU/mL (S3 Table).

For infections, 1.10^4^ NIH-3T3, Huh7.5 or 2.10^4^ I/1Ki cells were plated in 96-well plates and mixed with either 100 μL of viral supernatants, filtered cell supernatants from Huh7.5.1-HDV, NIH-3T3-RDeV or I/1Ki-SDeV or fresh DMEM (NIH-3T3 and Huh7.5) or MEM 20% FBS (I/1Ki) for non-infected controls. 24 h later, the media was replaced with DMEM 10% FBS (NIH-3T3 and Huh7.5) or MEM 20% FBS (I/1Ki). Cells were collected at the indicated time points for IF and RT-qPCR experiments.

### RNA isolation and quantitative Reverse Transcription PCR (qRT-PCR) applied to cells

At indicated times post-infection, cells were washed once with PBS 1X and lysed using the Power SYBR Green Cells-to-CT Kit (Invitrogen). Reverse Transcription (RT) and RT-qPCR were performed according to the Cell-to-Ct kit instructions on the CFX Opus 384 Real-Time PCR System (Bio-Rad, #12011452). All of the primers used are listed in S4 Table. Data were analyzed using the CFX manager suite from Bio-Rad.

### RNA isolation and qRT-PCR applied to tissue samples

Freshly dissected mouse liver tissue fragments were collected in 2mL tubes and flash frozen in liquid nitrogen. Total RNAs were extracted using the QIAshredder (Qiagen, #79654) and Rneasy mini kit (Qiagen #74004) according to the manufacturer’s instructions. cDNAs were synthetized from 1 μg of total RNA using the Maxima Minus cDNA Synthesis Master Mix kit (ThermoFisher #M1662) according to the manufacturer’s instructions. RT-qPCRs were performed using Power SYBR Green PCR Master Mix (Applied Biosystems #4367659) on the CFX Opus 384 Real-Time PCR System (Bio-Rad #12011452). All primers used are provided in S4 Table. Data were analyzed using the CFX manager suite from Bio-Rad.

### Immunofluorescence staining applied to cells

For immunofluorescence experiments on infected cells in 96-well plates, cells were washed with PBS (Eurobio #CS1PBS01-01) then fixed for 20 min at RT with 4% paraformaldehyde (Electron Microscopy Sciences #15714), permeabilized for 10 minutes in a 0.2% Triton X100 (Bio-Rad #1610407) PBS solution and blocked in 0.5% BSA (Sigma-Aldrich #A3059) for 1 h prior to an overnight incubation in primary antibodies at 4°C, followed by incubation in the dark with secondary antibodies and DAPI for 1 h. Cells were washed and immediately imaged using an ImageXpress Pico Automated Cell Imaging System (Molecular Devices) with a 4X lens to detect viral infection. Higher quality images of infected cells were acquired on an LSM980 8Y confocal microscope with a 10X lens.

For all other IF experiments cells were grown on microscope cover glasses (Marienfeld #0102052) in 6-well plates, washed 3 times with PBS then fixed and permeabilized using a 4% paraformaldehyde, 0.2% Triton X-100 PBS solution for 20 min at RT. Cover glasses were washed 3 times with a 0.1 M Tris-HCl, 0.15M NaCl solution and saturated in saturation buffer: PBS, 0.1% Triton X-100, 2% BSA solution for 30 min at RT before overnight incubation with primary antibodies in the same buffer at 4°C. Cover glasses were subsequently washed 3 times with a 0.1M Tris-HCl, 0.15M NaCl solution and incubated in the dark with secondary antibodies for 2 hours in the saturation buffer at RT and in the dark. Slides were incubated with 300nM DAPI (Invitrogen #D21490) in a 0.1M Tris-HCl, 0.15M NaCl solution for 15 min at RT and in the dark and washed 3 times 5 min in a 0.1M Tris-HCl, 0.15M NaCl solution. Cover glasses where then mounted in ProLong Gold antifade reagent (Invitrogen #P36930), left to polymerize overnight at RT and in the dark then sealed with nail polish.

Polyclonal antibodies extracted from patient sera (Ig-Patient1) diluted 1:500 were used to detect the various delta antigens, monoclonal mouse anti-dsRNA J2 IgG (Jenna Bioscience #RNT-SCI-10010200) diluted 1:500 was used to detect dsRNA and monoclonal mouse anti-polR2A IgG (8WG16, Invitrogen #MA1-26249) diluted 1:50 was used to detect RNAPII. Alexa Fluor 488-conjugated goat anti-human IgG secondary antibody (Invitrogen #A11013) diluted 1:500 was used to detect human primary antibodies, Alexa Fluor 488-conjugated goat anti-mouse IgG secondary antibody (Invitrogen #A11001) and Alexa Fluor 555-conjugated donkey anti-mouse IgG secondary antibody (Invitrogen #A31570) diluted 1:500 were used to detect mouse primary antibodies.

### Immunofluorescence staining applied to tissue samples

Freshly dissected mouse liver tissue fragments were frozen in OCT (Thermo #12678646) in liquid nitrogen cooled isopentane and stored at −80°C. 10-μM-thick tissue sections were obtained after cryosection, mounted on Superfrost Plus Gold slides (Thermo Scientific #K5800AMNZ72) and stored at −80°C. Slides were thawed at RT, and rehydrated for 5 min in PBS then fixed in a 4% paraformaldehyde, PBS solution for 30 min at RT, washed 3 times in PBS, permeabilized for 30 min in a 1% a Triton X-100, PBS solution at RT, washed 3 times with PBS and saturated in a 0.5% BSA, PBS solution for 30 min at RT.

Incubation with primary antibodies was performed overnight in a 0.01% BSA, PBS solution at 4°C. Slices were washed 3 times with PBS and incubated with secondary antibodies for 2h in a 0.01% BSA, PBS solution at RT. Slices were incubated with 300nM DAPI (Invitrogen #D21490) in a PBS solution for 15 min at RT and in the dark and washed 3 times 5 min with PBS. Samples were mounted between the Superfrost Plus Gold slides and microscope cover glasses in ProLong Gold antifade reagent, left to polymerize overnight at RT and in the dark then sealed with nail polish. Antibody dilutions were the same as described for cells.

### Single molecule in situ hybridization immuno-fluorescence (smFISHIF) staining applied to cells

Cells were grown on microscope cover glasses (Marienfeld #0102052) in 6-well plates, washed 3 times with PBS (Eurobio #CS1PBS01-01) and fixed and permeabilized using a 4% paraformaldehyde (Electron Microscopy Sciences #15714), 0.2% Triton X-100 (Bio-Rad #1610407) PBS solution for 20 min at RT. Cover glasses were washed 3 times in PBS, saturated in a PBS 0.5% ultrapure BSA (ThermoFisher Scientific #AM2616) solution for 30 min at RT and washed for 20min in a 10% formamide (Merck #F9037), 2X SSC (Invitrogen #AM9770) solution before overnight incubation in a 10% formamide, 2X SSC, 8% dextran sulfate (Sigma-Aldrich #D8906), 0.34mg/mL *E*. *coli* tRNA (Roche #10109541001), 1mM vanadyl ribonucleoside complex (VRC, Merck #R3380), 0.01% ultrapure BSA buffer containing 125nM Cy5 coupled smFISH probe mix or 2% smiFISH Cy5 duplexed probe mix (probes used are listed in S2 Table) and the required antibodies at 37°C and in the dark. FLAP-Y containing smiFISH probes were previously duplexed with Cy5 conjugated FLAP-Y probes in the following conditions: 68ng/ μL probe mix, 7 μM Cy5 conjugated FLAP-Y probes, 10% NEBuffer 3 (New England Biolabs #B7003S) in a thermocycler at 85°C for 3 min, 65°C for 3 min and 25°C for 5 min. Cover glasses were subsequently washed twice for 15 min at 37°C in a 10% formamide, 2X SSC solution, incubated for 1h in a 0.01% ultrapure BSA, 10% formamide, 2X SSC containing the required secondary antibodies and then in a 300nM DAPI (Invitrogen #D21490), 10% formamide, 2X SSC solution, for 15 min at 37°C in the dark and washed 3 times 5 min in a 2X SSC, 0.1% Tween 20 (Thermo Scientific #J20605-AP), PBS solution. Cover glasses were then mounted in ProLong Gold antifade reagent (Invitrogen #P36930), left to polymerize overnight at RT and in the dark then sealed with nail polish.

Polyclonal antibodies extracted from patient sera (Ig-Patient1) diluted 1:500 were used to detect the various delta antigens, monoclonal mouse anti-dsRNA J2 IgG (Jenna Bioscience #RNT-SCI-10010200) diluted 1:500 was used to detect dsRNA. Alexa Fluor 488-conjugated goat anti-human IgG secondary antibody (Invitrogen #A11001) diluted 1:500 was used to detect human primary antibodies, Alexa Fluor 488-conjugated goat anti-mouse IgG secondary antibody (Invitrogen #A11001) was used to detect mouse primary antibodies.

### Single molecule in situ hybridization immuno-fluorescence (smFISHIF) staining applied to tissue samples

Freshly dissected mouse liver tissue fragments were frozen in OCT in liquid nitrogen-cooled isopentane and stored at −80°C. 10-μM-thick tissue sections were mounted on Superfrost Plus Gold slides (Thermo Scientific #K5800AMNZ72) and stored at −80°C. Slides were thawed at RT, and rehydrated for 5 min in PBS then fixed in a 4% paraformaldehyde, PBS solution for 30 min at RT, washed 3 times in PBS and permeabilized for 30 min in a 1% Triton X-100, PBS solution at RT, washed 3 times in a 0.1% Tween 20 PBS solution, saturated in a 0.5% ultrapure BSA, 0.1% Tween 20, PBS solution for 30 min at RT and washed in a 10% formamide, 2X SSC solution for 20 min at RT. Overnight incubation and all following steps were performed as described for cells. Samples were mounted between the Superfrost Plus Gold slides and microscope cover glasses in ProLong Gold antifade reagent, left to polymerize overnight at RT in the dark then sealed with nail polish. Antibody dilutions were the same as described for cells.

### Ethynyl-Uridine incorporation and detection

Cells were grown on microscope cover glasses (Marienfeld #0102052) in 6-well plates and incubated for 1 h in medium containing 1 mM EU, washed 3 times with PBS (Eurobio #CS1PBS01-01) and fixed and permeabilized using a 4% paraformaldehyde (Electron Microscopy Sciences #15714), 0.2% Triton X-100 (Bio-Rad #1610407) PBS solution for 20 min at RT. Cover glasses were washed 3 times in PBS and incorporated EU were detected using the Click-iT RNA Alexa Fluor 488 Imaging Kit (Invitrogen #C10329) according to the manufacturer’s instructions. Following EU staining cover glasses were washed 3 times with a 0.1 M Tris-HCl, 0.15M NaCl and the following saturation and antibody stainings were performed as described above but in the dark.

### Microscopy and imaging

Immunofluorescence on mice liver slices was detected using an Axioscan 7 (Zeiss) equipped with a Set Orca hamamatsu Flash 4.0 V2 Axio Scan, using a dry 20x objective and controlled using Zen blue (Zeiss, version 3.7). Unless otherwise specified, IF and smFISHIF on cells and smFISHIF on liver slices were acquired using a Zeiss LSM980 confocal microscope (controlled with Zen blue 3.7) on an Airyscan 2 detector in Super Resolution mode with a 40X oil objective 1.3NA. GFP/Alexa-488 was excited using a 488 nm laser, Cy3/Alexa-555 were excited using a 561 nm laser, Alexa-670 was excited using a 633 nm laser. Image post-processing was performed using the FIJI software [86], Zen blue (Zeiss, version 3.7) and Illustrator (Adobe Systems).

### Fluorescence signal quantification

Fluorescence signal quantification in hollow hubs was done following these steps: i) confocal images were loaded in FIJI [86] ii) a 3 μm line ROI was positioned to cross the hub in the larger region iii) Fluorescence intensity along the ROI was recovered at three Z planes (apical, mid and basal of the hubs) for the Ag and genome signals iv) intensity for the Ag and genome signals were plotted using Prism (GraphPad).

## Supporting information

S1 Table Primers used for cloning and PCR. (XLSX)

S2 Table Probes used for sm/smiFISH and Northern Blot. (XLSX)

S3 Table VSV-G-pseudotyped viral titers. (XLSX)

S4 Table Primers used for RT-qPCR. (XLSX)

S1 Fig DAgs homology and patient sera reactivity. A-B) Tables recapitulating homologies between the full-length amino acid sequences of DAgs (A) and between regions corresponding to characterized HDV domains (B) 1. Structural and functional regions previously identified in HDAg. 2. Sequence conservation profile calculated from a sequence alignment of HDAg, RDAg and SDAg using AL2CO[79] by applying a sliding average on a 10-residue window. 3. Predicted disorder propensity along the amino acid sequence for HDAg, RDAg and SDAg (shown as black, red and green lines, respectively). 4. Alphafold2 per-residue pLDDT (predicted local distance difference test) score versus residue number for the best scoring model of each DAg. The color code is the same as in 3. C) DAg detection using purified patient sera. Huh7.5 cells were transfected with an empty pcDNA3.1 plasmid or with pcDNA3.1 plasmids encoding FLAG tagged DAgs from human (small), rodent, avian, toad, or snake deltaviruses. Cells were collected 3 days post-transfection and protein extracts were analyzed by western blot to detect DAgs expression and the presence of FLAG tags. Purified sera from 6 patients and from a rabbit immunized with SDAg were used. β-actin served as a loading control. D) Phylogenetic tree showing the relationships of HDAg (genotype 1), RDAg, SDAg, duck-associated DAg and toad DAg. Phylogenetic relationships were assessed by the maximum likelihood method available within PhyML (version 3.0, 87]. The significance of the branching order was estimated by the bootstrap method (1000 resampling). Only values >70% are shown.
(TIF)

S2 Fig ADAR-1 editing and DAg accumulation. A) Schematic representation of the ADAR-1 locus targeted by the different sgRNAs for KO generation (*Upper*) and the ADAR-1 isoforms, p110 and p150, including the functional domains for each isoform and the localization of the sgRNAs targeted regions (*Lower*). Western blot analysis of WT, ADAR-1 KO or overexpressing cells lines. Protein extracts from ADAR-1 overexpressing (p110 or p150), ADAR-1 KO (p110 and p150 or p150 only) and control Huh7.5 and HEK293T cell lines were analyzed by western blot to detect ADAR-1 expression and β-actin B) Western blot analysis of the human (*Upper*), rodent (*Lower*) DAg forms in ADAR-1 overexpressing (p110 or p150), ADAR-1 KO (p110 and p150 or p150 only) and control Huh7.5 (*Left panels*) and HEK293T (*Right panels*) cell lines. Cells were transfected with an empty pcDNA3.1 plasmid or with pcDNA3.1 plasmids encoding dimers of the HDV or RDeV genomes and collected 9 days post-transfection (d.p.t.) for HDV transfected Huh7.5 cells, 6 d.p.t for HDV transfected HEK293T cells, 6 d.p.t. for RDeV transfected Huh7.5 and HEK293T cells. Protein extracts were analyzed by western blot for DAg and β-actin expression.
(TIF)

S3 Fig Generation and characterization of cell lines persistently replicating kolmiovirids. A) Schematic representation of the process to generate persistently replicating cell lines. B) Northern Blot detection of HDV, RDeV and SDeV genomes and anti-genomes in cells persistently replicating kolmiovirids. U6 and 18S ribosomal RNAs serve as loading controls. C) PCR detection of the transfected HDV, RDeV and SDeV plasmids in cells persistently replicating kolmiovirids. Universal primers serve as positive controls and the corresponding mock cell lines as negative controls.
(TIF)

S4 Fig RNA polymerase II localization in the nucleus of cells persistently replicating kolmiovirids. Huh7.5.1-HDV (A), NIH-3T3-RDeV (B) and I/1Ki-SDeV (C) cells were plated on microscopy slides and fixed to visualize DAgs and RNAPII localization in the nucleus. Corresponding non-replicating cell lines served as negative controls. Representative confocal images are shown. Nuclei (in blue) were stained using DAPI and DAgs (in green) and RNAP II (in orange) were detected by IF, *scale bars* 10 μm. Cells were imaged on a LSM980 confocal microscope (Zeiss) and analyzed using ImageJ (version 2.9.0).
(TIF)

S1 Video 3D reconstitution of a persistently replicating Huh7.5.1-HDV nucleus. Confocal Z-stack images from the persistently replicating nucleus presented in Fig 5A were used to create and animate a 3D model using Imaris (version 9.9.1).
(MP4)

S2 Video 3D reconstitution of a persistently replicating NIH-3T3-RDeV nucleus. Confocal Z-stacks images from the persistently replicating nucleus presented in Fig 5B were used to create and animate a 3D model using Imaris (version 9.9.1).
(MP4)

S3 Video 3D reconstitution of a persistently replicating I/1Ki-SDeV nucleus. Confocal Z-stacks images from the persistently replicating nucleus presented in Fig 5C were used to create and animate a 3D model using Imaris (version 9.9.1).
(MP4)

S4 Video 3D reconstitution of an HDV transfected mouse hepatocyte nucleus. Confocal Z-stacks images from the transfected nucleus presented in Fig 6E were used to create and animate a 3D model using Imaris (version 9.9.1).
(MP4)

S5 Video 3D reconstitution of an RDeV transfected mouse hepatocyte nucleus. Confocal Z-stacks images from the transfected nucleus presented in Fig 6E were used to create and animate a 3D model using Imaris (version 9.9.1).
(MP4)

S6 Video 3D reconstitution of an SDeV transfected mouse hepatocyte nucleus. Confocal Z-stacks images from the transfected nucleus presented in Fig 6E were used to create and animate a 3D model using Imaris (version 9.9.1).
(MP4)

S1 Raw Data Raw data. (ZIP)
