# Supplementary figures and images for: Comparative analysis of human, rodent and snake deltavirus replication
Source: PLoS Pathog. 2024 Mar 5;20(3):e1012060. doi: 10.1371/journal.ppat.1012060 (PMC10942263; doi:10.1371/journal.ppat.1012060)

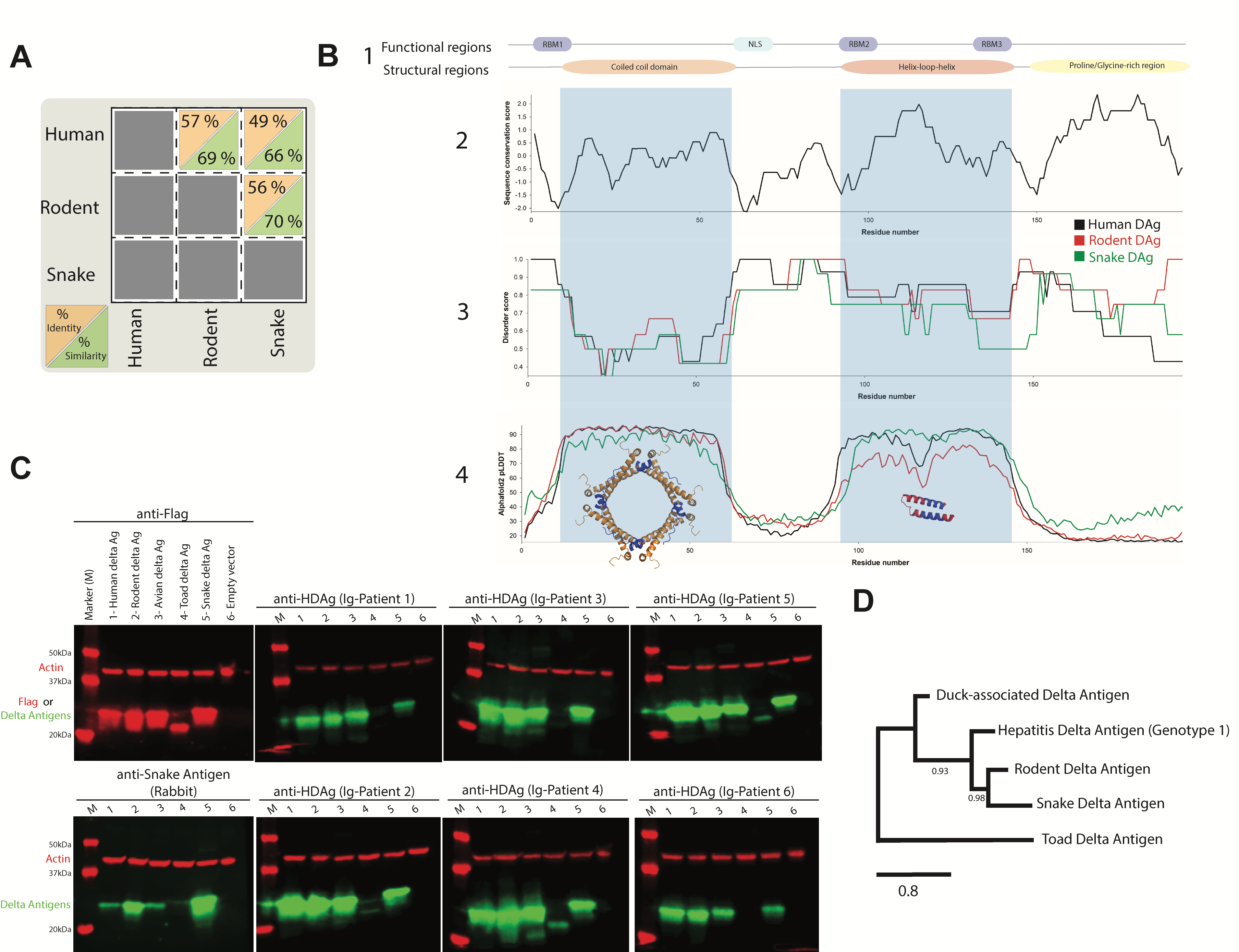

Supplement: S1 Fig — A-B) Tables recapitulating homologies between the full-length amino acid sequences of DAgs (A) and between regions corresponding to characterized HDV domains (B) 1. Structural and functional regions previously identified in HDAg. 2. Sequence conservation profile calculated from a sequence alignment of HDAg, RDAg and SDAg using AL2CO[79] by applying a sliding average on a 10-residue window. 3. Predicted disorder propensity along the amino acid sequence for HDAg, RDAg and SDAg (shown as black, red and green lines, respectively). 4. Alphafold2 per-residue pLDDT (predicted local distance difference test) score versus residue number for the best scoring model of each DAg. The color code is the same as in 3. C) DAg detection using purified patient sera. Huh7.5 cells were transfected with an empty pcDNA3.1 plasmid or with pcDNA3.1 plasmids encoding FLAG tagged DAgs from human (small), rodent, avian, toad, or snake deltaviruses. Cells were collected 3 days post-transfection and protein extracts were analyzed by western blot to detect DAgs expression and the presence of FLAG tags. Purified sera from 6 patients and from a rabbit immunized with SDAg were used. β-actin served as a loading control. D) Phylogenetic tree showing the relationships of HDAg (genotype 1), RDAg, SDAg, duck-associated DAg and toad DAg. Phylogenetic relationships were assessed by the maximum likelihood method available within PhyML (version 3.0, 87]. The significance of the branching order was estimated by the bootstrap method (1000 resampling). Only values >70% are shown. (TIF) [file ppat.1012060.s005.tif]

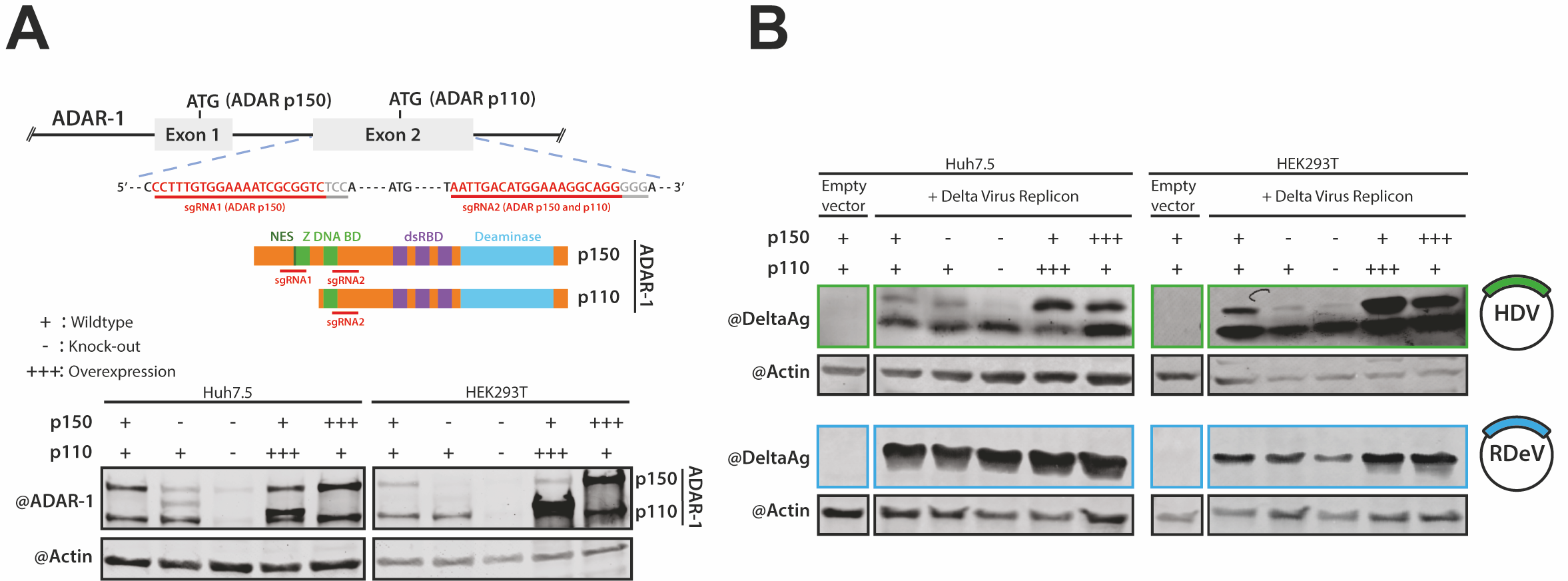

Supplement: S2 Fig — A) Schematic representation of the ADAR-1 locus targeted by the different sgRNAs for KO generation (Upper) and the ADAR-1 isoforms, p110 and p150, including the functional domains for each isoform and the localization of the sgRNAs targeted regions (Lower). Western blot analysis of WT, ADAR-1 KO or overexpressing cells lines. Protein extracts from ADAR-1 overexpressing (p110 or p150), ADAR-1 KO (p110 and p150 or p150 only) and control Huh7.5 and HEK293T cell lines were analyzed by western blot to detect ADAR-1 expression and β-actin B) Western blot analysis of the human (Upper), rodent (Lower) DAg forms in ADAR-1 overexpressing (p110 or p150), ADAR-1 KO (p110 and p150 or p150 only) and control Huh7.5 (Left panels) and HEK293T (Right panels) cell lines. Cells were transfected with an empty pcDNA3.1 plasmid or with pcDNA3.1 plasmids encoding dimers of the HDV or RDeV genomes and collected 9 days post-transfection (d.p.t.) for HDV transfected Huh7.5 cells, 6 d.p.t for HDV transfected HEK293T cells, 6 d.p.t. for RDeV transfected Huh7.5 and HEK293T cells. Protein extracts were analyzed by western blot for DAg and β-actin expression. (TIF) [file ppat.1012060.s006.tif]

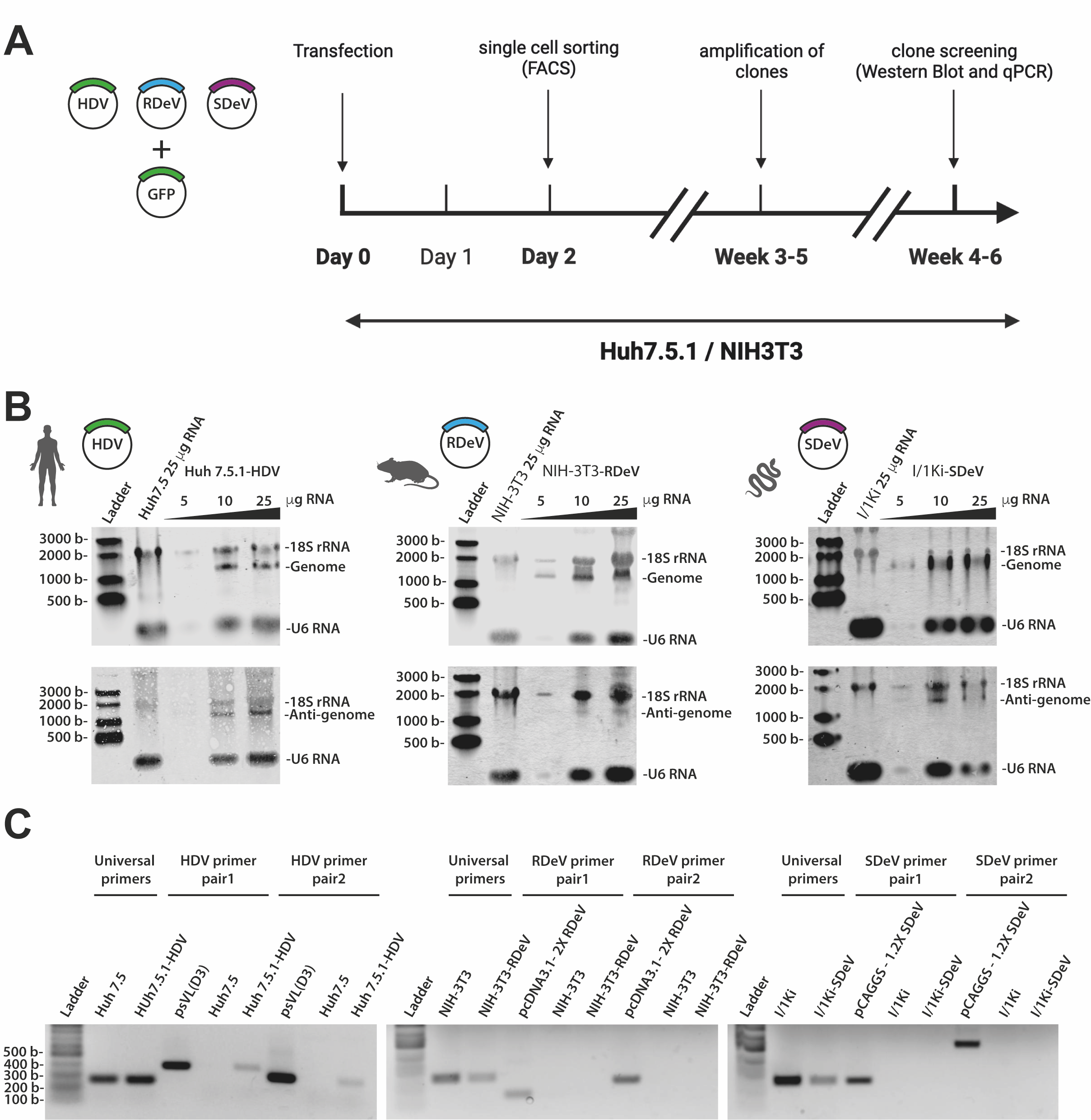

Supplement: S3 Fig — A) Schematic representation of the process to generate persistently replicating cell lines. B) Northern Blot detection of HDV, RDeV and SDeV genomes and anti-genomes in cells persistently replicating kolmiovirids. U6 and 18S ribosomal RNAs serve as loading controls. C) PCR detection of the transfected HDV, RDeV and SDeV plasmids in cells persistently replicating kolmiovirids. Universal primers serve as positive controls and the corresponding mock cell lines as negative controls. (TIF) [file ppat.1012060.s007.tif]

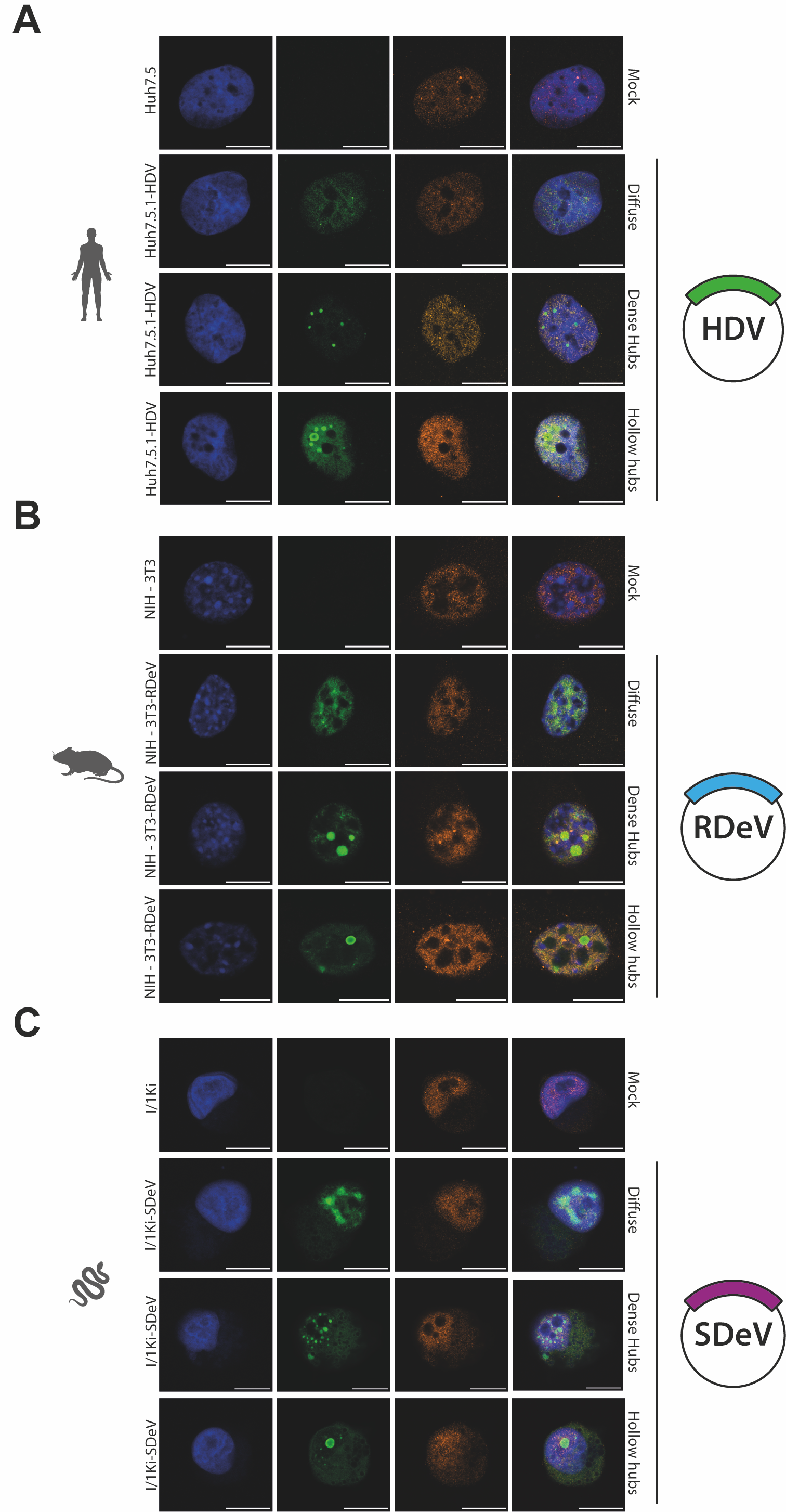

Supplement: S4 Fig — Huh7.5.1-HDV (A), NIH-3T3-RDeV (B) and I/1Ki-SDeV (C) cells were plated on microscopy slides and fixed to visualize DAgs and RNAPII localization in the nucleus. Corresponding non-replicating cell lines served as negative controls. Representative confocal images are shown. Nuclei (in blue) were stained using DAPI and DAgs (in green) and RNAP II (in orange) were detected by IF, scale bars 10 μm. Cells were imaged on a LSM980 confocal microscope (Zeiss) and analyzed using ImageJ (version 2.9.0). (TIF) [file ppat.1012060.s008.tif]
