# Supplementary material for: Comparative analysis of human, rodent and snake deltavirus replication
Source: PLoS Pathog. 2024 Mar 5;20(3):e1012060. doi: 10.1371/journal.ppat.1012060 (PMC10942263; doi:10.1371/journal.ppat.1012060)
Supplement: S1 Raw Data — (ZIP) [file ppat.1012060.s015.zip › Figure 1 raw western membranes.pptx]

## Slide 1
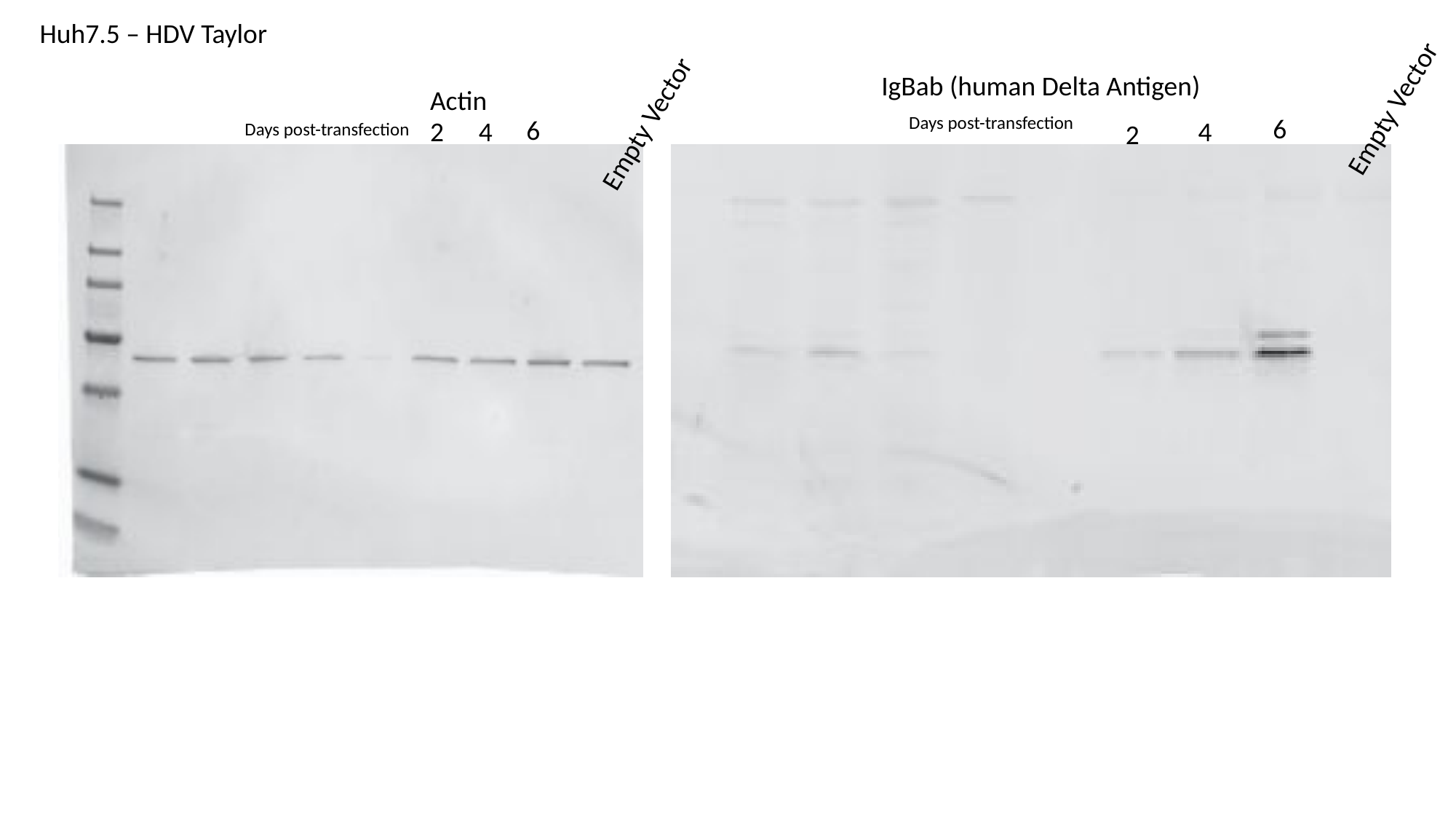

Huh7.5 – HDV Taylor
Empty Vector
Empty Vector
IgBab (human Delta Antigen)
Actin
Days post-transfection
6
6
2
4
4
Days post-transfection
2

## Slide 2
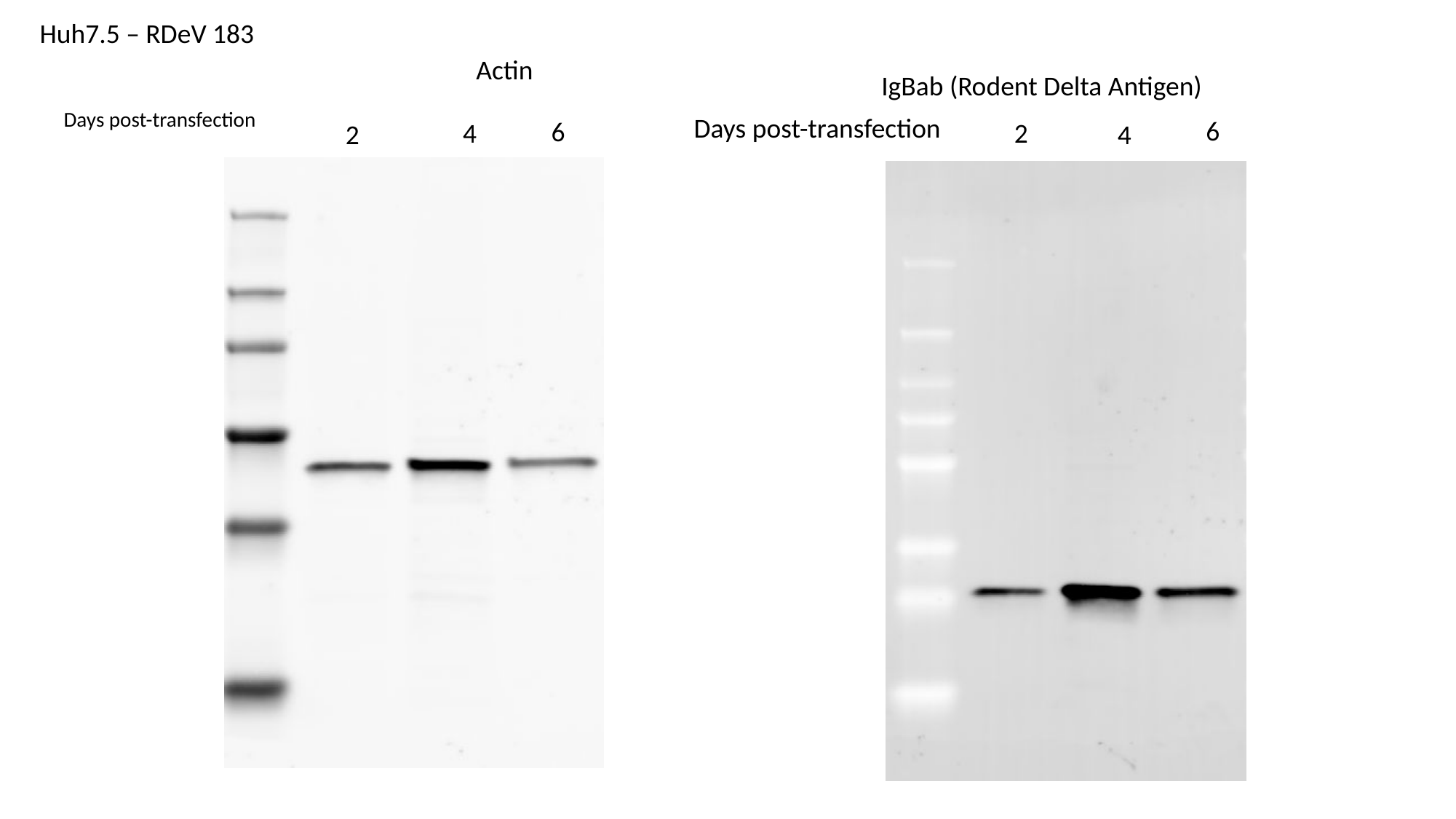

Huh7.5 – RDeV 183
Actin
IgBab (Rodent Delta Antigen)
Days post-transfection
Days post-transfection
6
6
4
2
2
4

## Slide 3
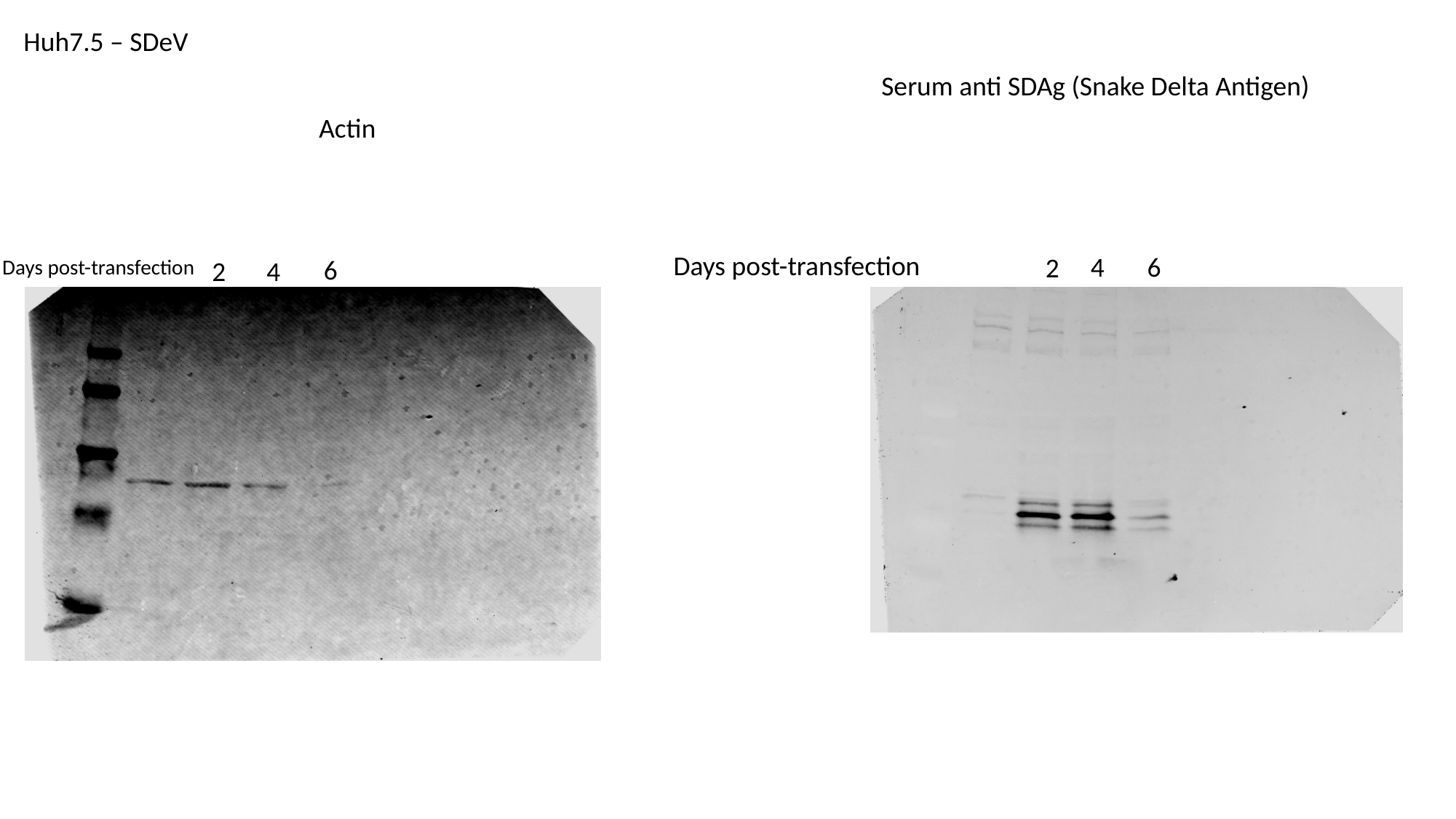

Huh7.5 – SDeV
Serum anti SDAg (Snake Delta Antigen)
Actin
Days post-transfection
4
6
2
6
Days post-transfection
2
4

## Slide 4
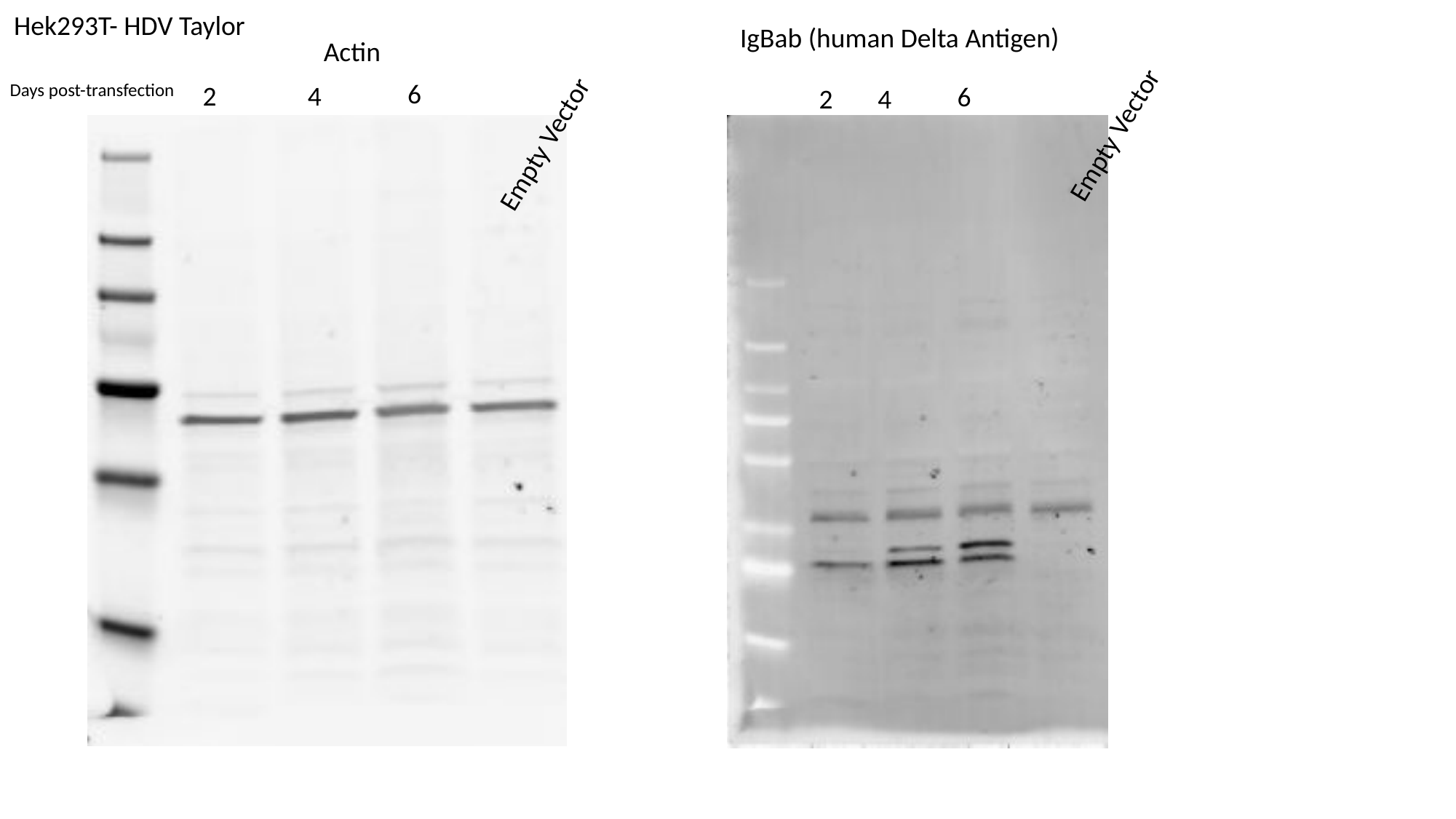

Hek293T- HDV Taylor
IgBab (human Delta Antigen)
Actin
Empty Vector
6
Days post-transfection
2
4
6
Empty Vector
2
4

## Slide 5
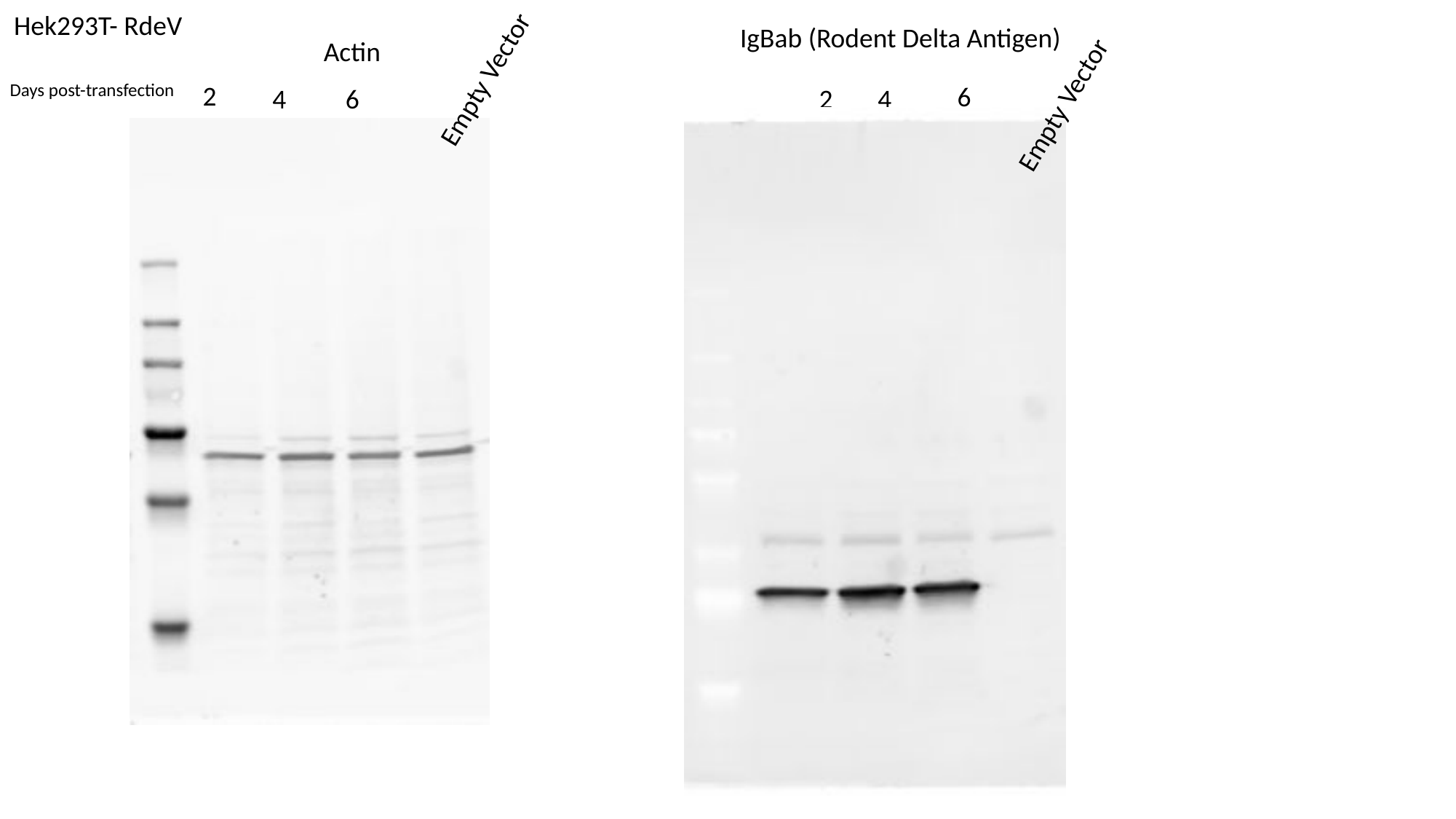

Hek293T- RdeV
Empty Vector
IgBab (Rodent Delta Antigen)
Actin
Empty Vector
Days post-transfection
2
6
4
6
2
4

## Slide 6
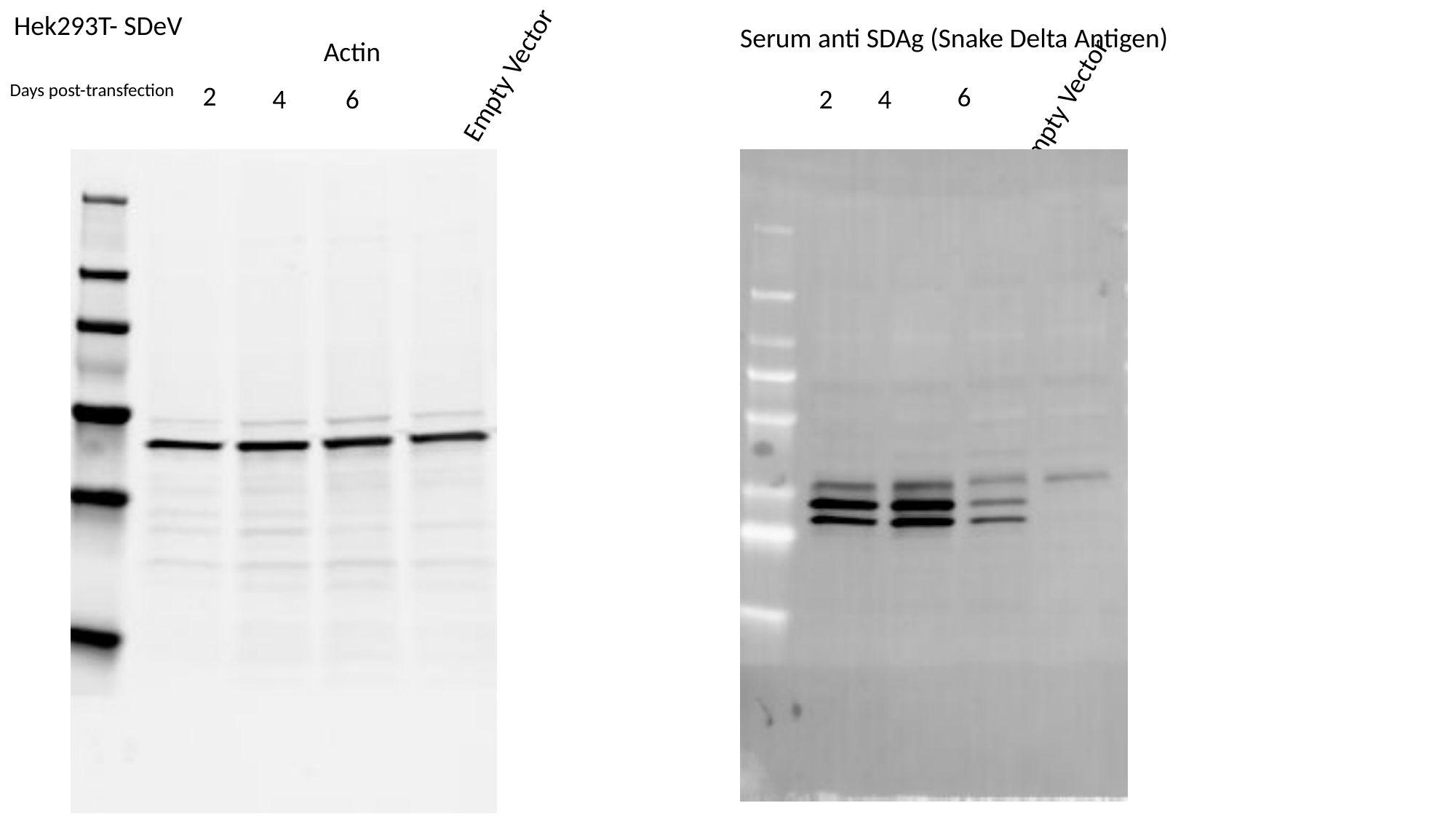

Hek293T- SDeV
Empty Vector
Serum anti SDAg (Snake Delta Antigen)
Actin
Empty Vector
Days post-transfection
2
6
4
6
2
4

## Slide 7
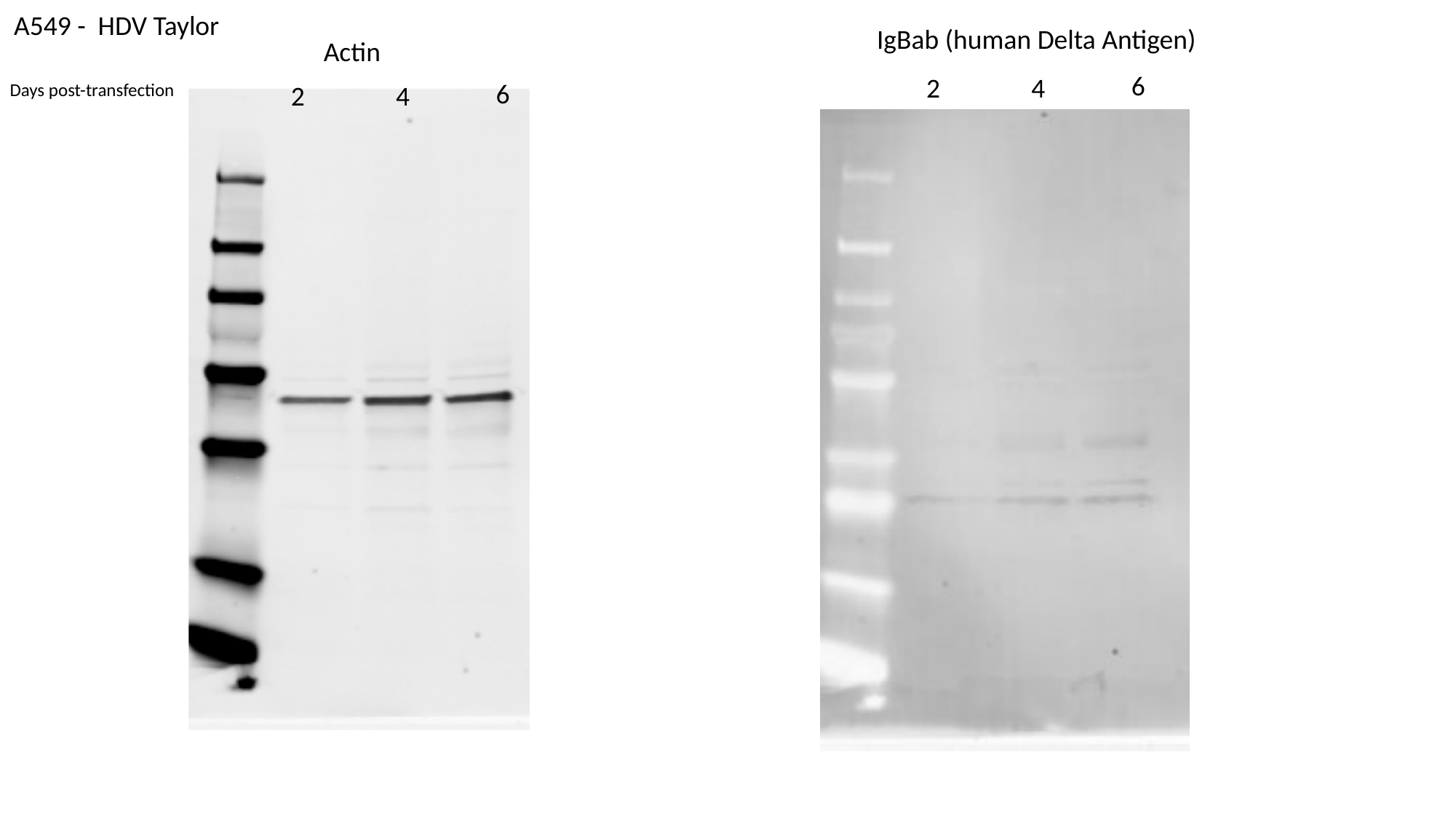

A549 - HDV Taylor
IgBab (human Delta Antigen)
Actin
6
2
4
6
Days post-transfection
2
4

## Slide 8
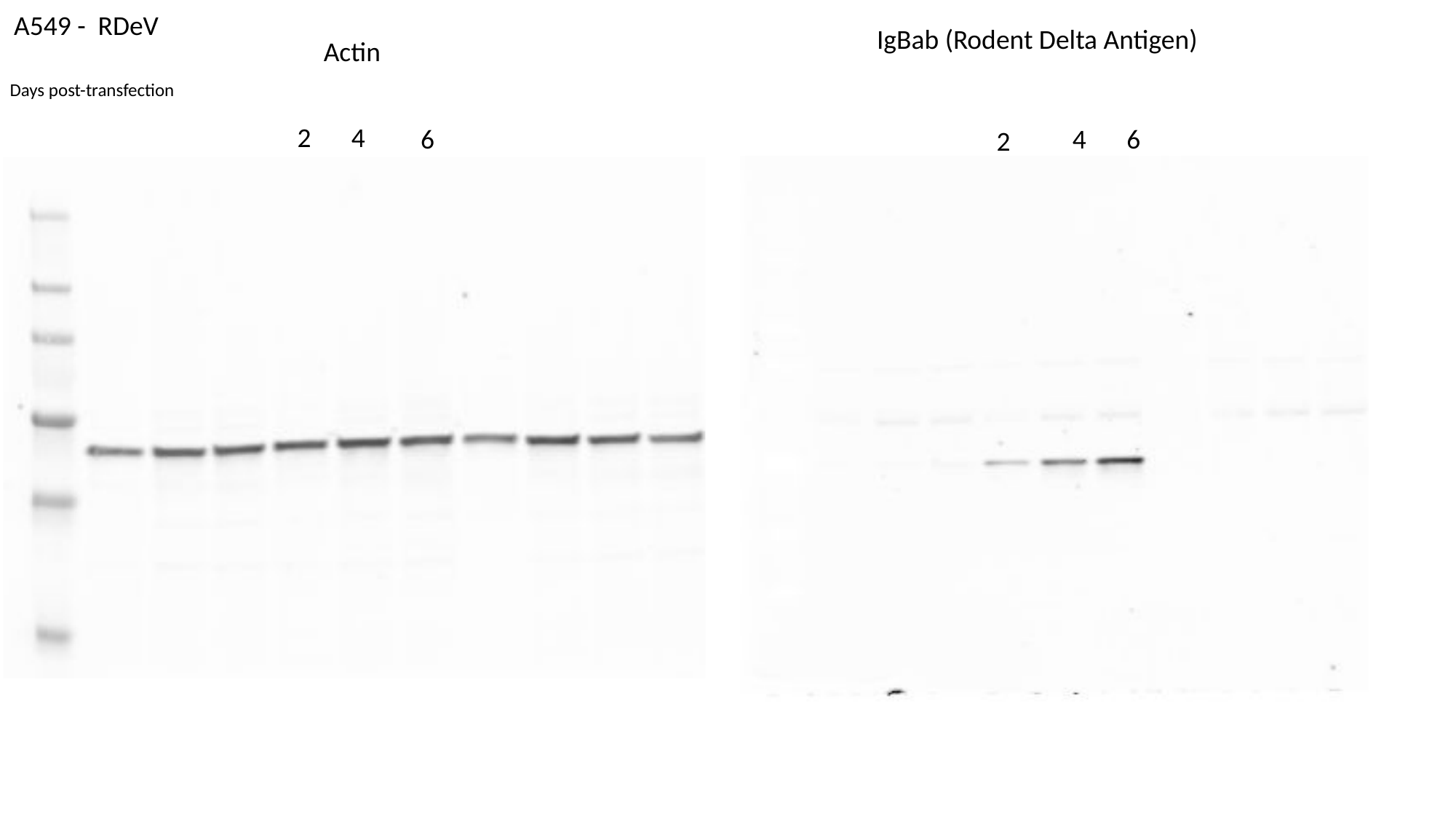

A549 - RDeV
IgBab (Rodent Delta Antigen)
Actin
Days post-transfection
2
4
6
4
6
2

## Slide 9
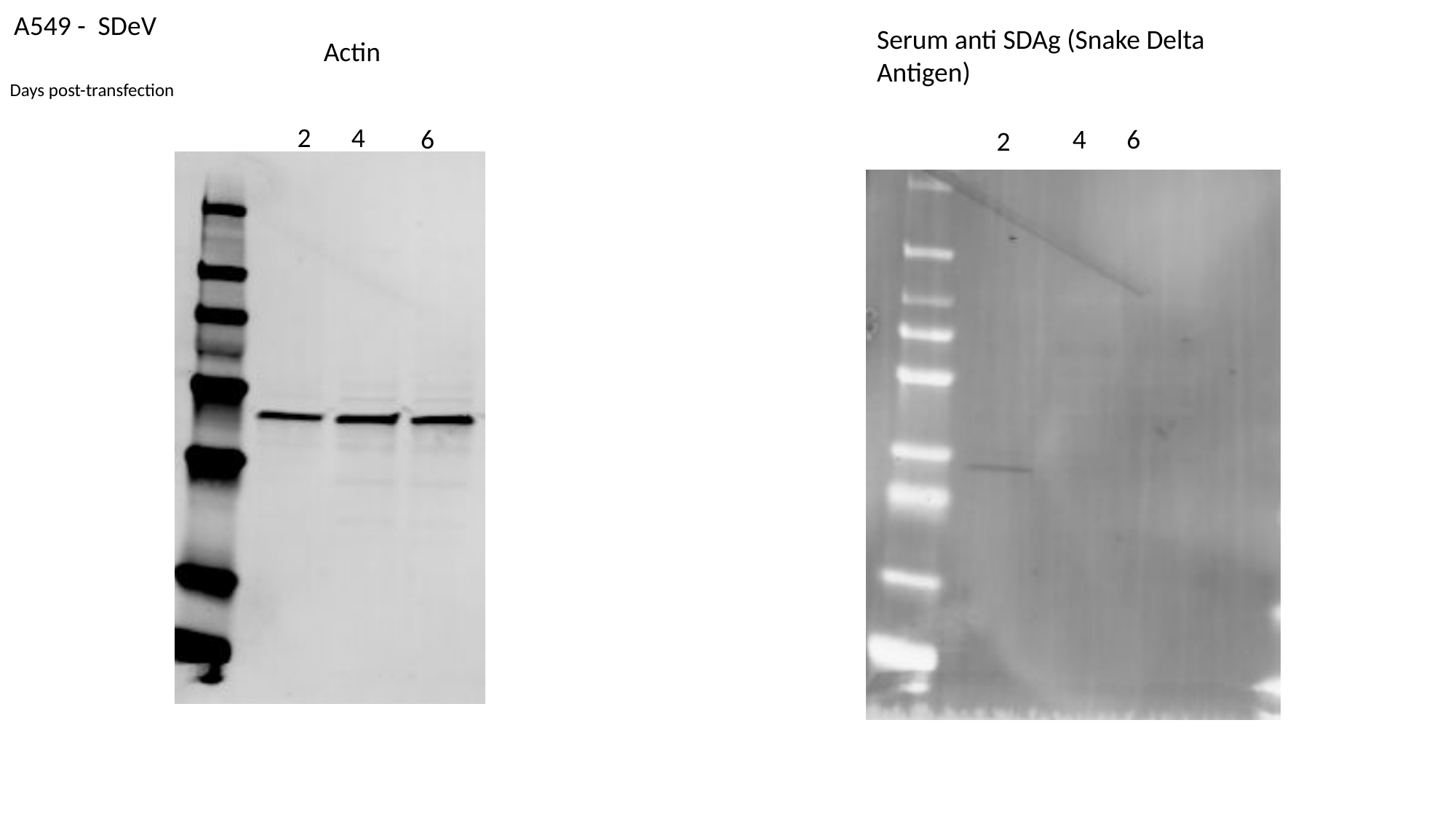

A549 - SDeV
Serum anti SDAg (Snake Delta Antigen)
Actin
Days post-transfection
2
4
6
4
6
2

## Slide 10
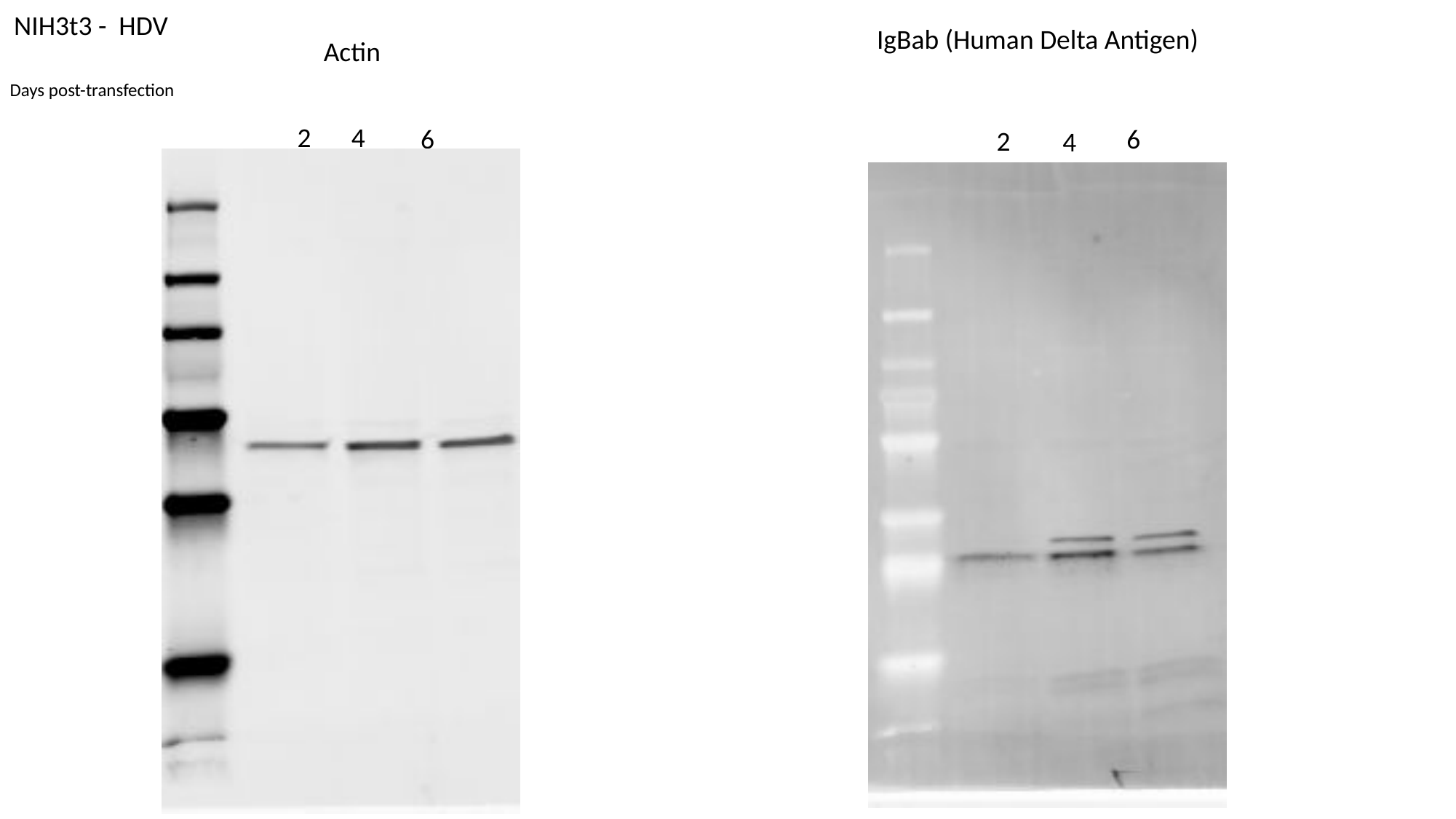

NIH3t3 - HDV
IgBab (Human Delta Antigen)
Actin
Days post-transfection
2
4
6
6
2
4

## Slide 11
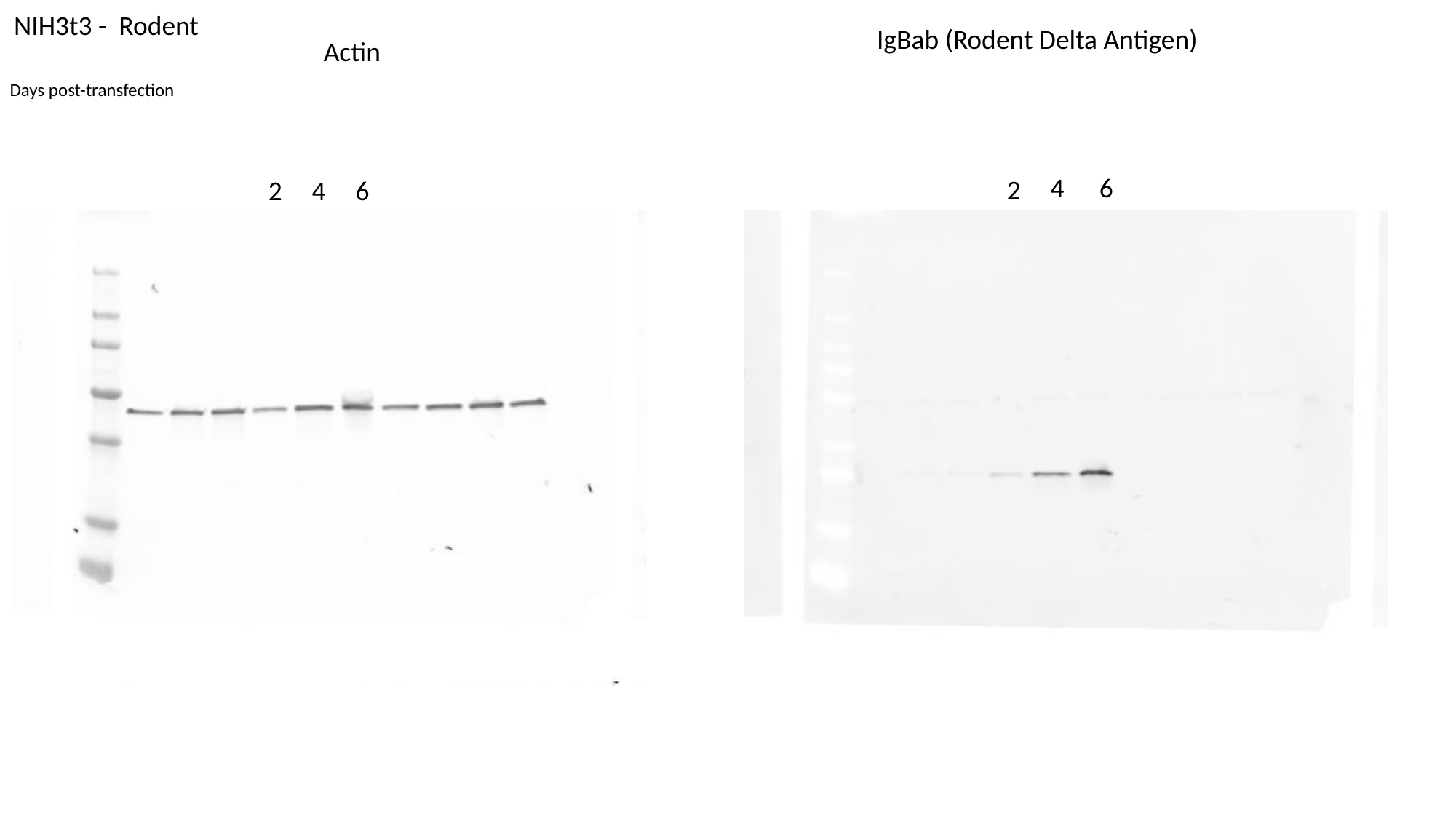

NIH3t3 - Rodent
IgBab (Rodent Delta Antigen)
Actin
Days post-transfection
4
6
2
2
4
6

## Slide 12
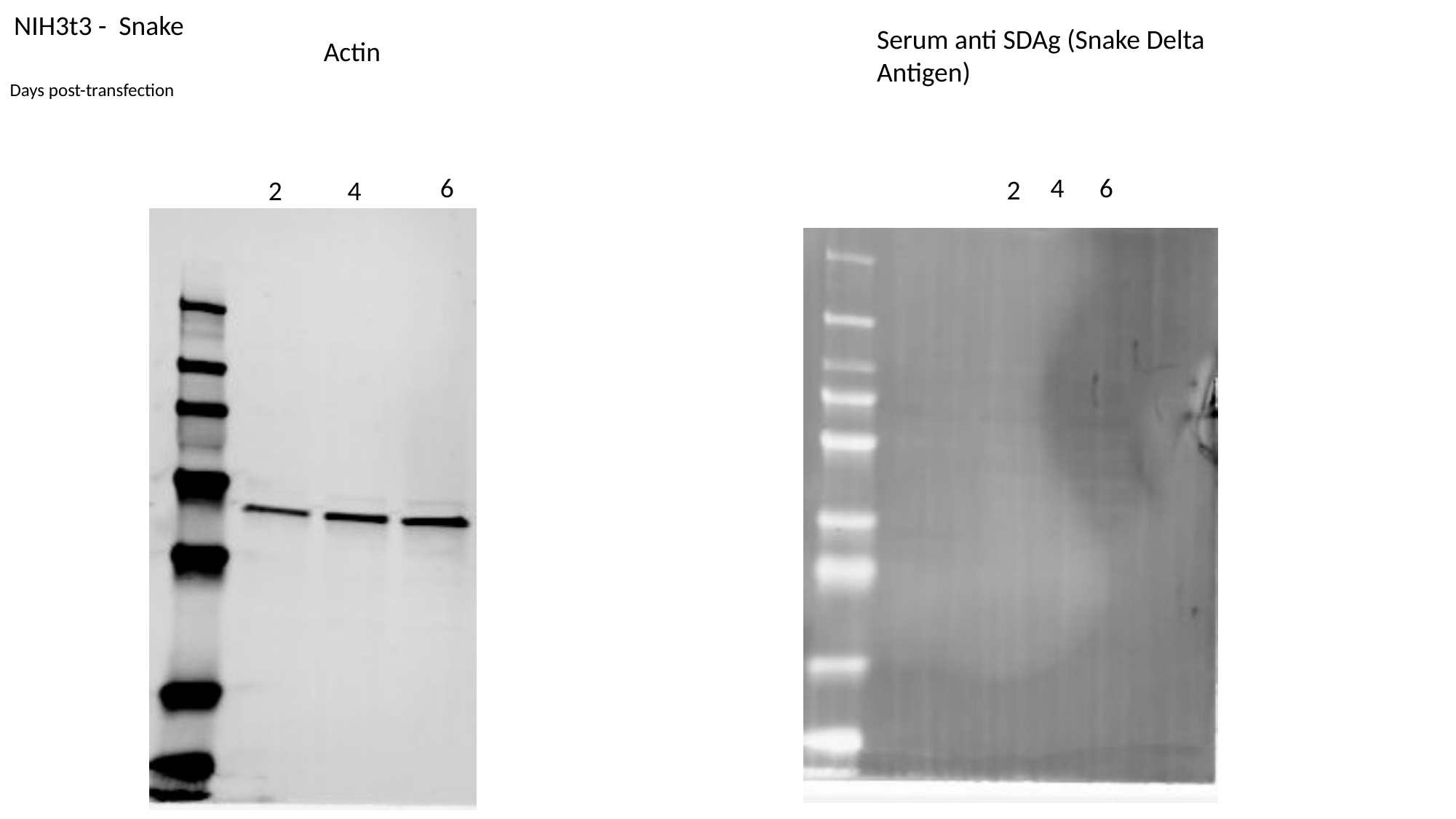

NIH3t3 - Snake
Serum anti SDAg (Snake Delta Antigen)
Actin
Days post-transfection
6
4
6
2
2
4

## Slide 13
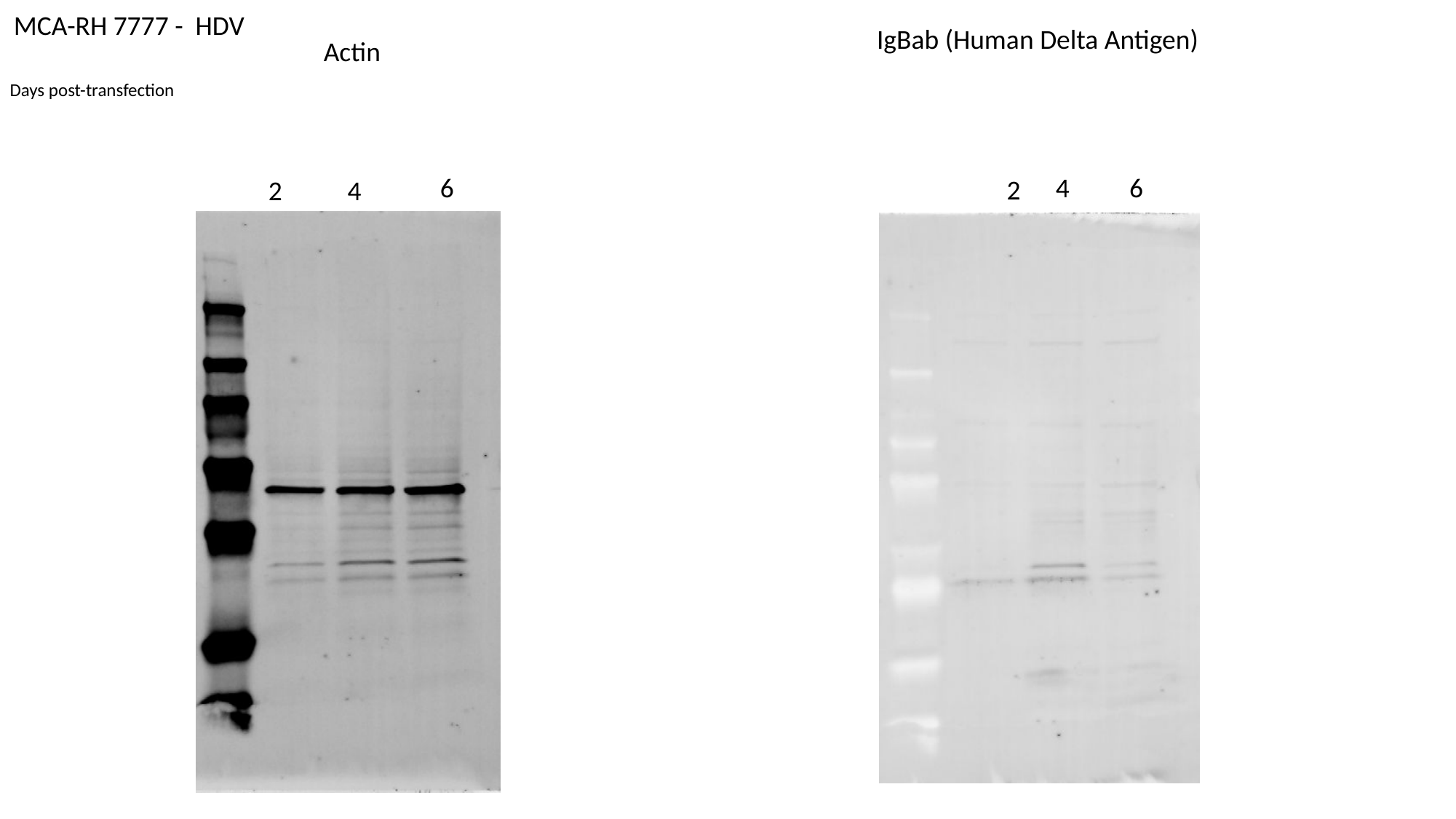

MCA-RH 7777 - HDV
IgBab (Human Delta Antigen)
Actin
Days post-transfection
6
4
6
2
2
4

## Slide 14
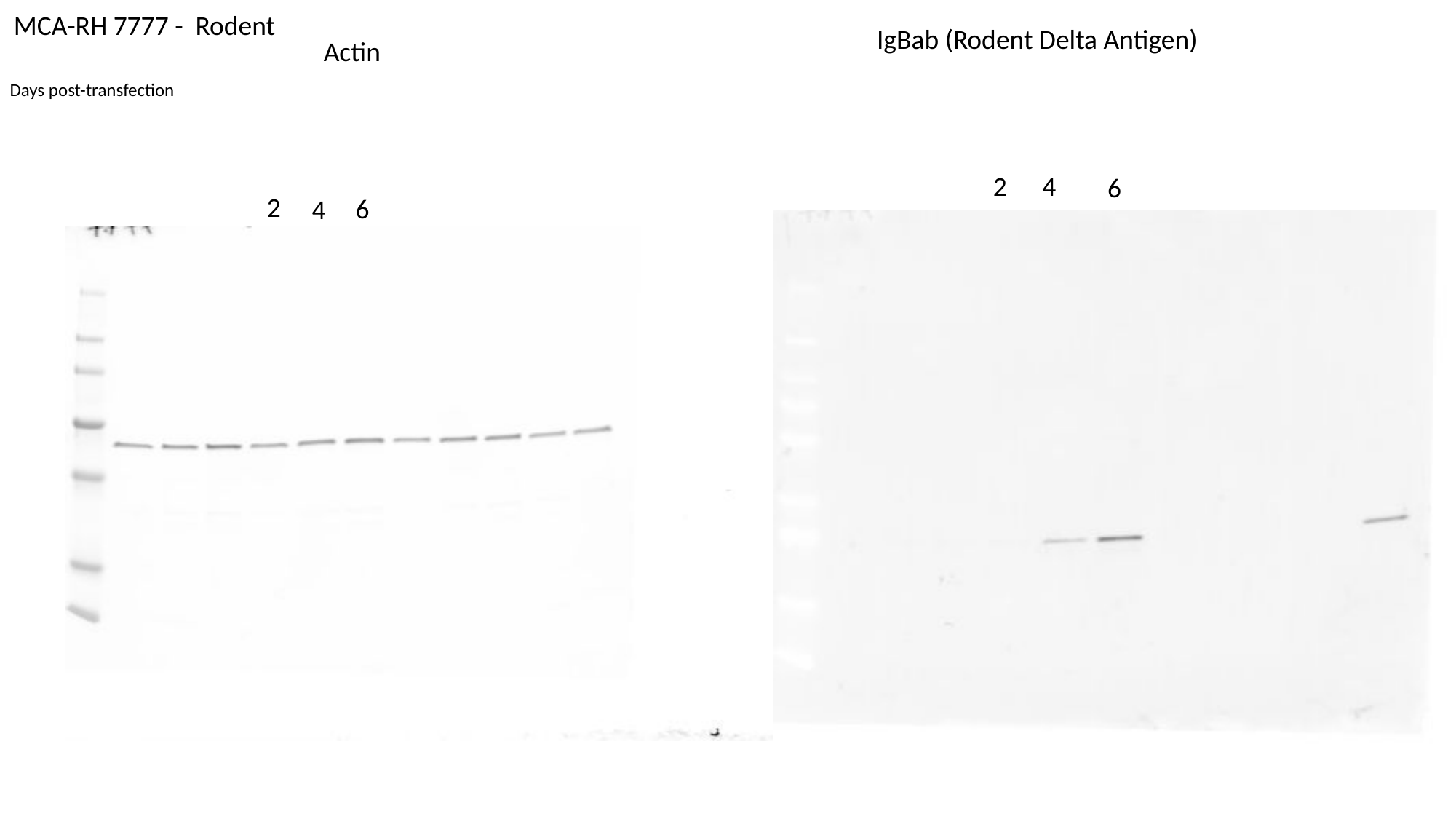

MCA-RH 7777 - Rodent
IgBab (Rodent Delta Antigen)
Actin
Days post-transfection
2
4
6
2
6
4

## Slide 15
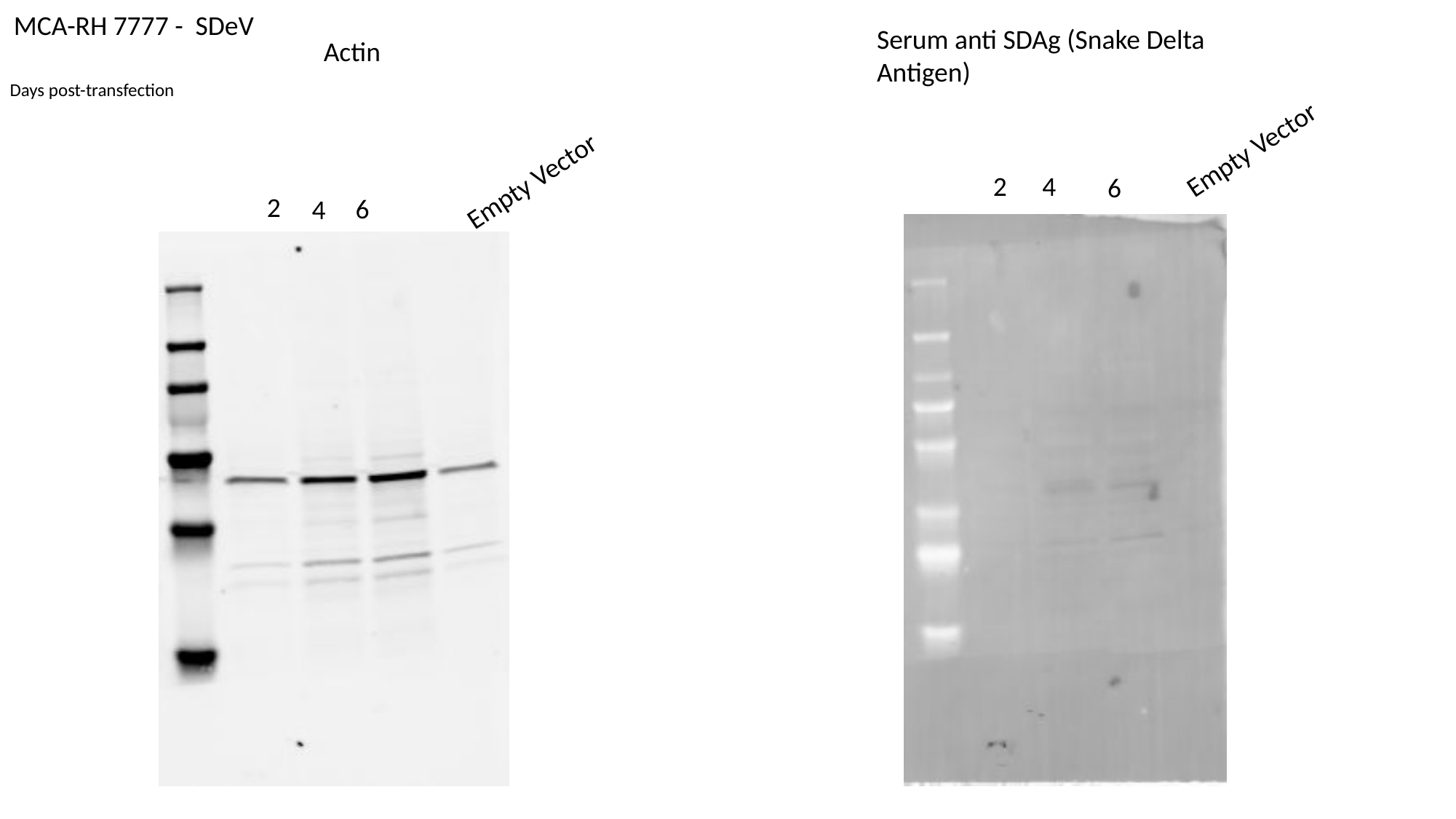

MCA-RH 7777 - SDeV
Serum anti SDAg (Snake Delta Antigen)
Actin
Days post-transfection
Empty Vector
Empty Vector
2
4
6
2
6
4

## Slide 16
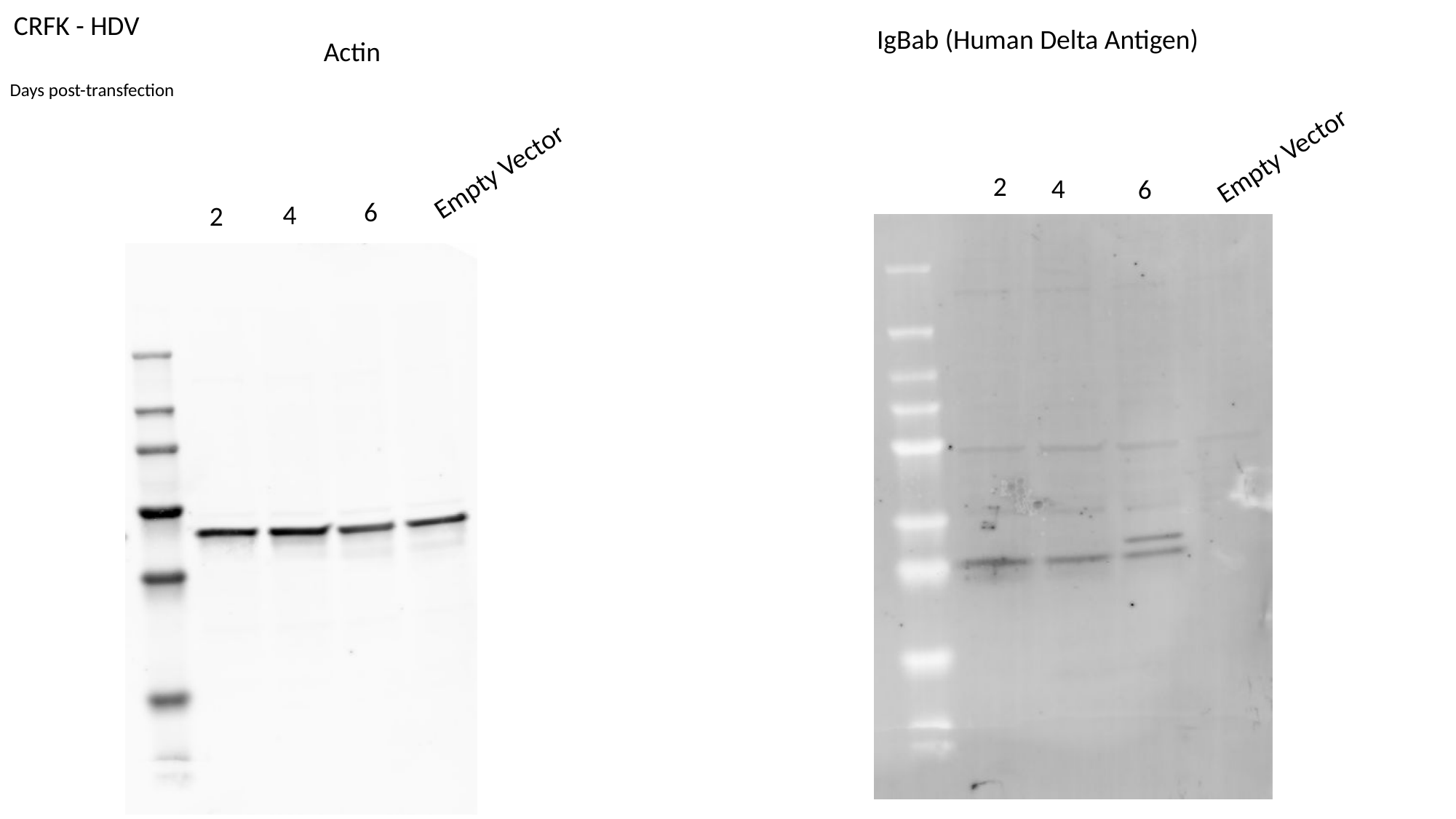

CRFK - HDV
IgBab (Human Delta Antigen)
Actin
Days post-transfection
Empty Vector
Empty Vector
2
4
6
6
4
2

## Slide 17
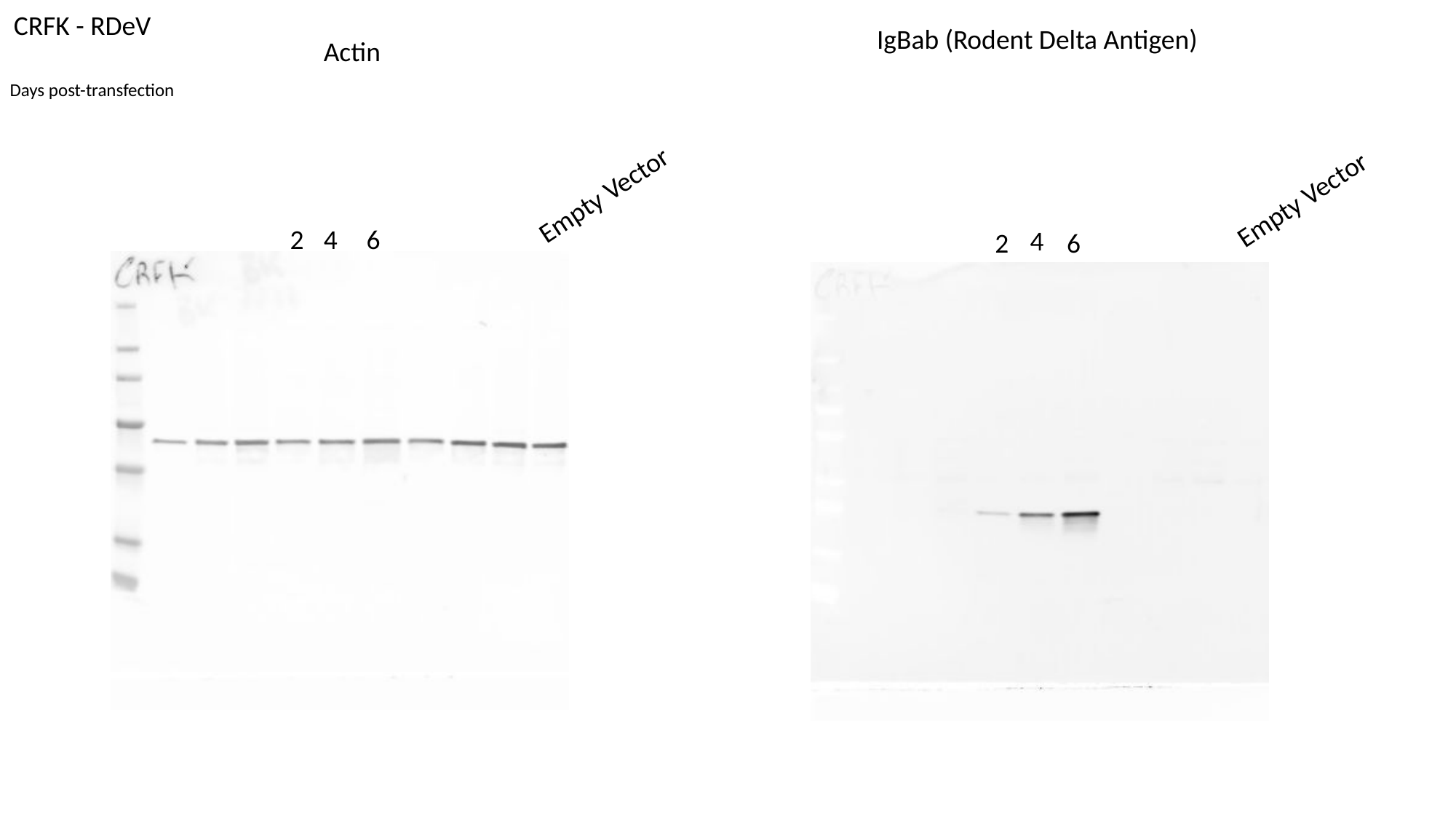

CRFK - RDeV
IgBab (Rodent Delta Antigen)
Actin
Days post-transfection
Empty Vector
Empty Vector
2
4
6
4
2
6

## Slide 18
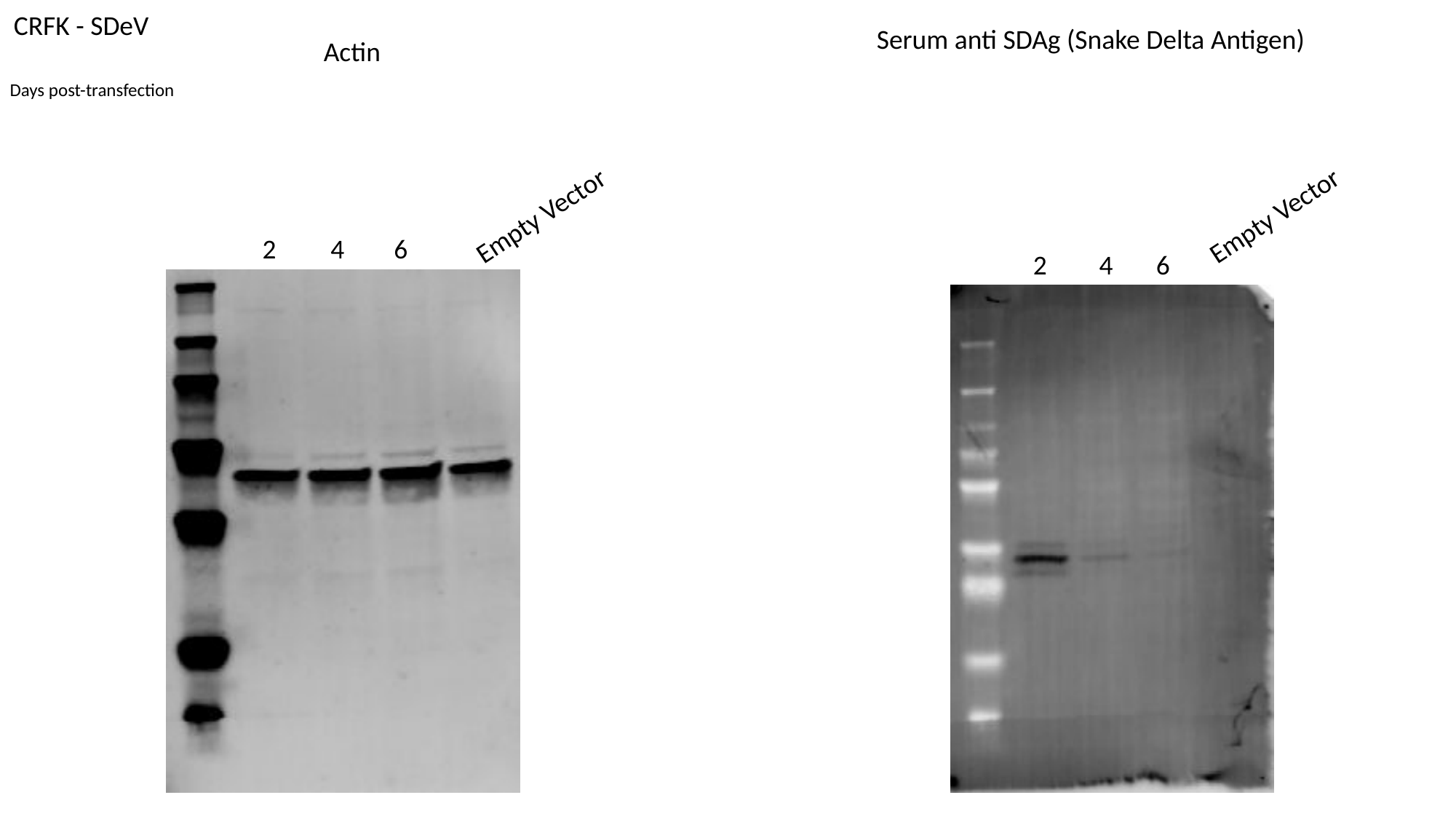

CRFK - SDeV
Serum anti SDAg (Snake Delta Antigen)
Actin
Days post-transfection
Empty Vector
Empty Vector
2
4
6
4
6
2

## Slide 19
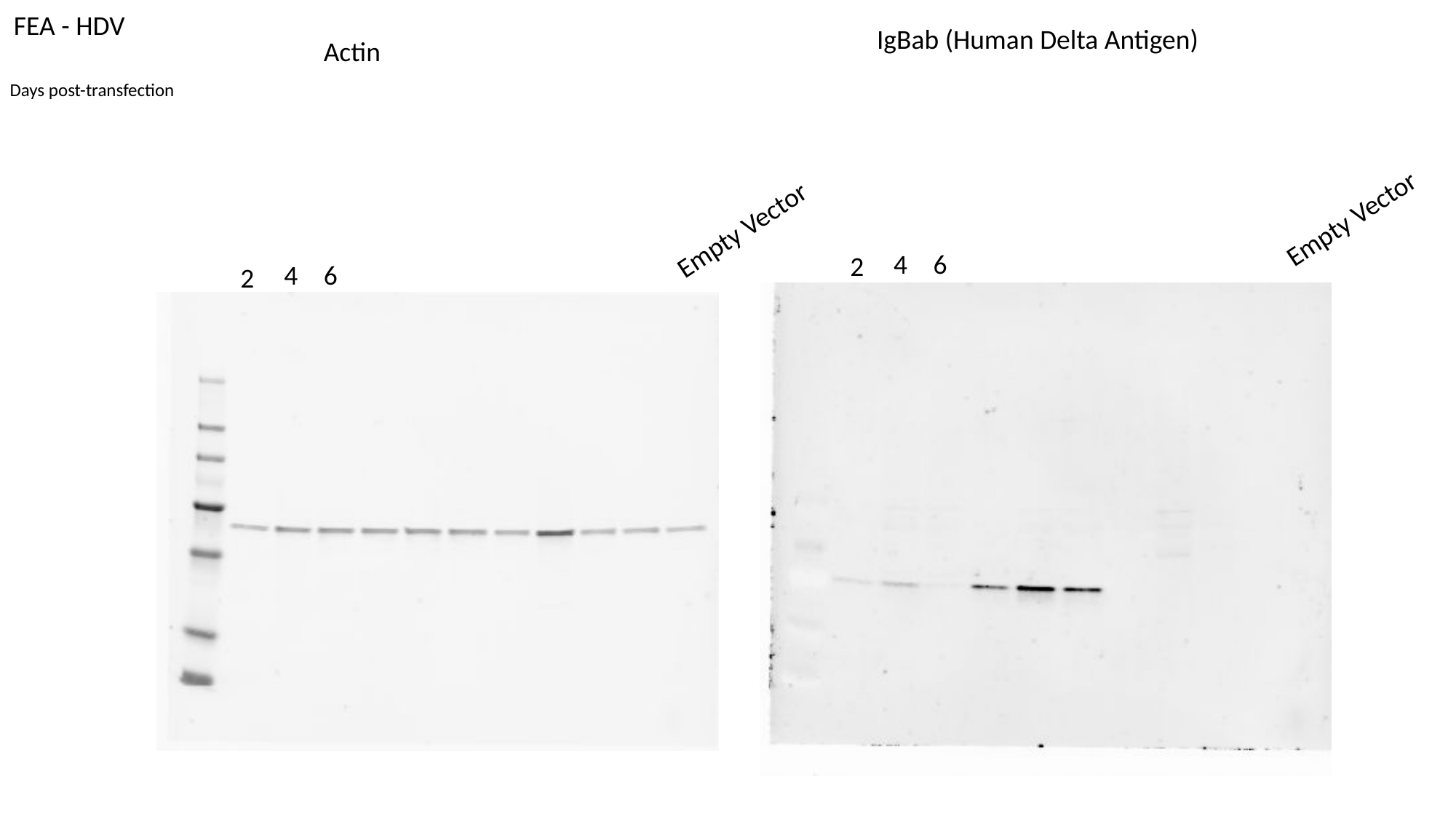

FEA - HDV
IgBab (Human Delta Antigen)
Actin
Days post-transfection
Empty Vector
Empty Vector
4
6
2
4
6
2

## Slide 20
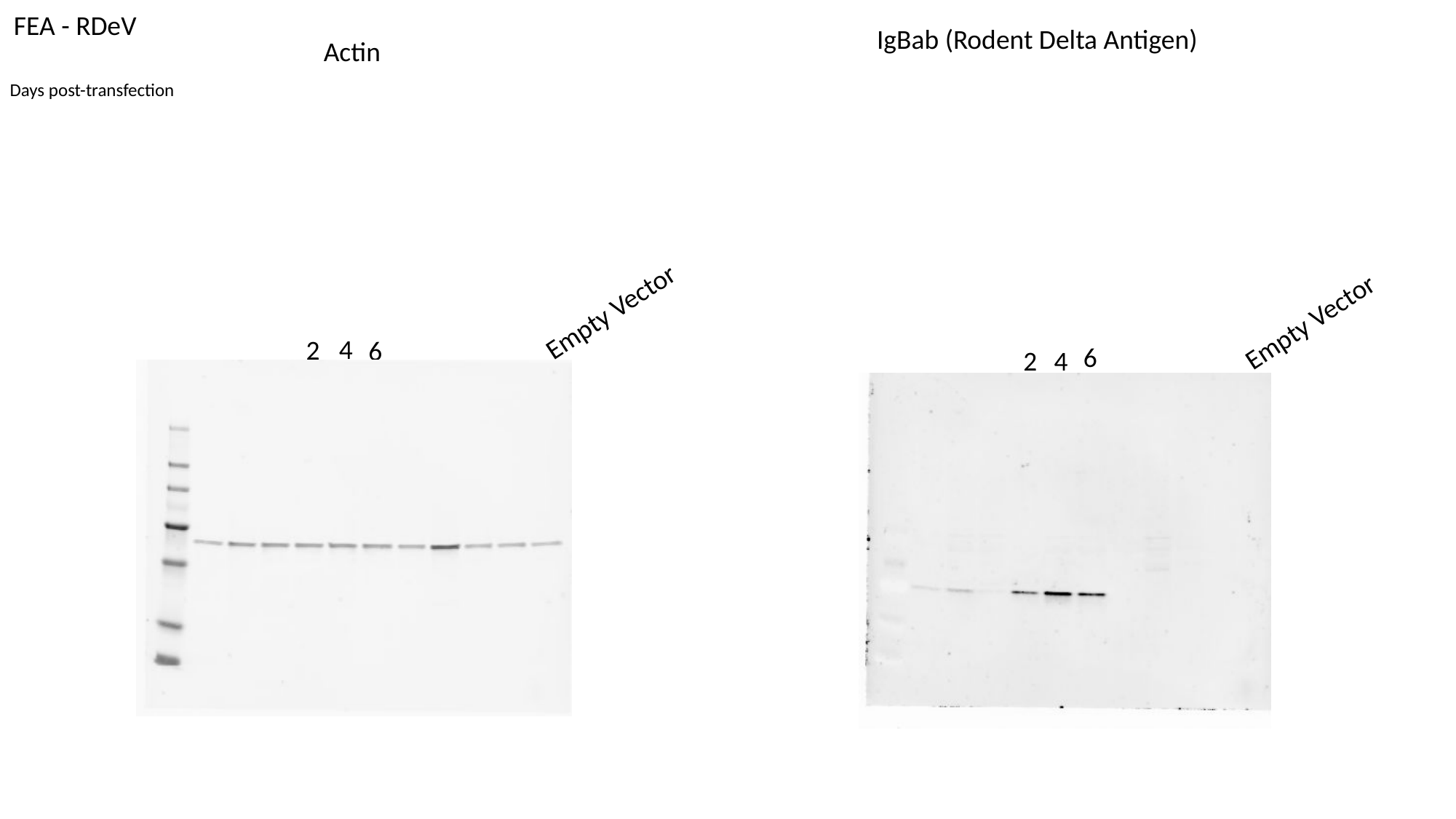

FEA - RDeV
IgBab (Rodent Delta Antigen)
Actin
Days post-transfection
Empty Vector
Empty Vector
4
2
6
6
2
4

## Slide 21
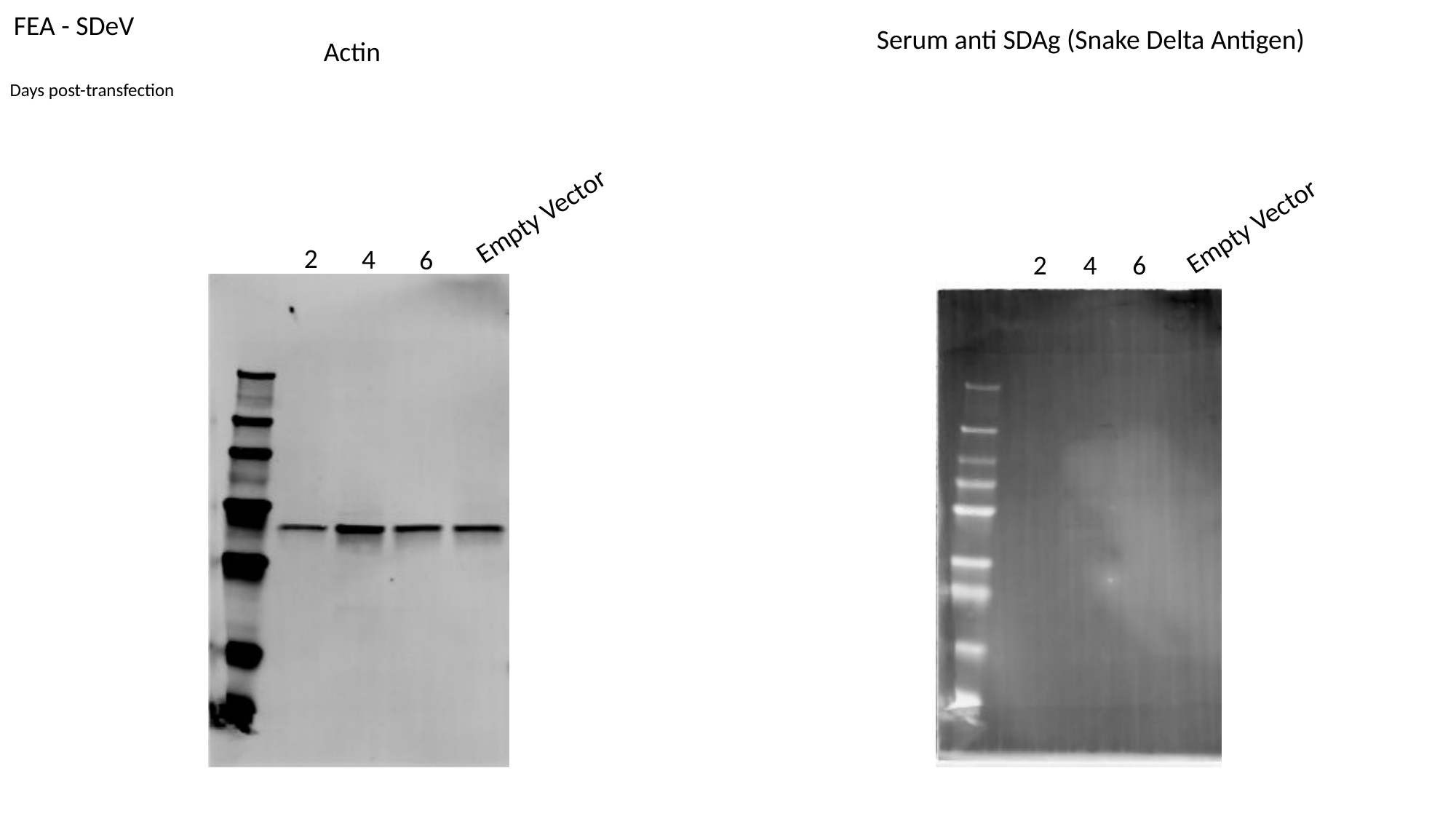

FEA - SDeV
Serum anti SDAg (Snake Delta Antigen)
Actin
Days post-transfection
Empty Vector
Empty Vector
2
4
6
4
6
2

## Slide 22
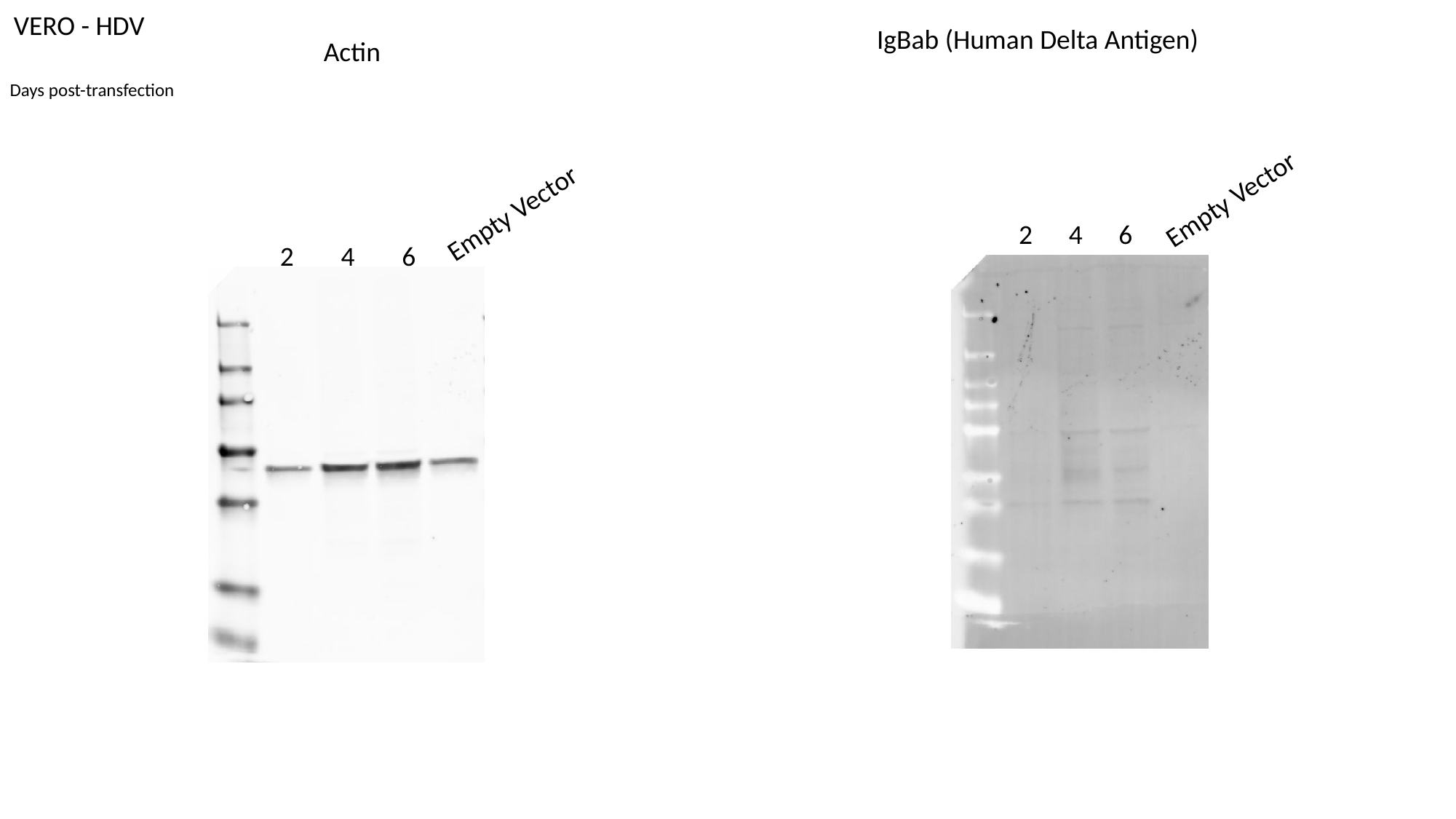

VERO - HDV
IgBab (Human Delta Antigen)
Actin
Days post-transfection
Empty Vector
Empty Vector
2
4
6
2
4
6

## Slide 23
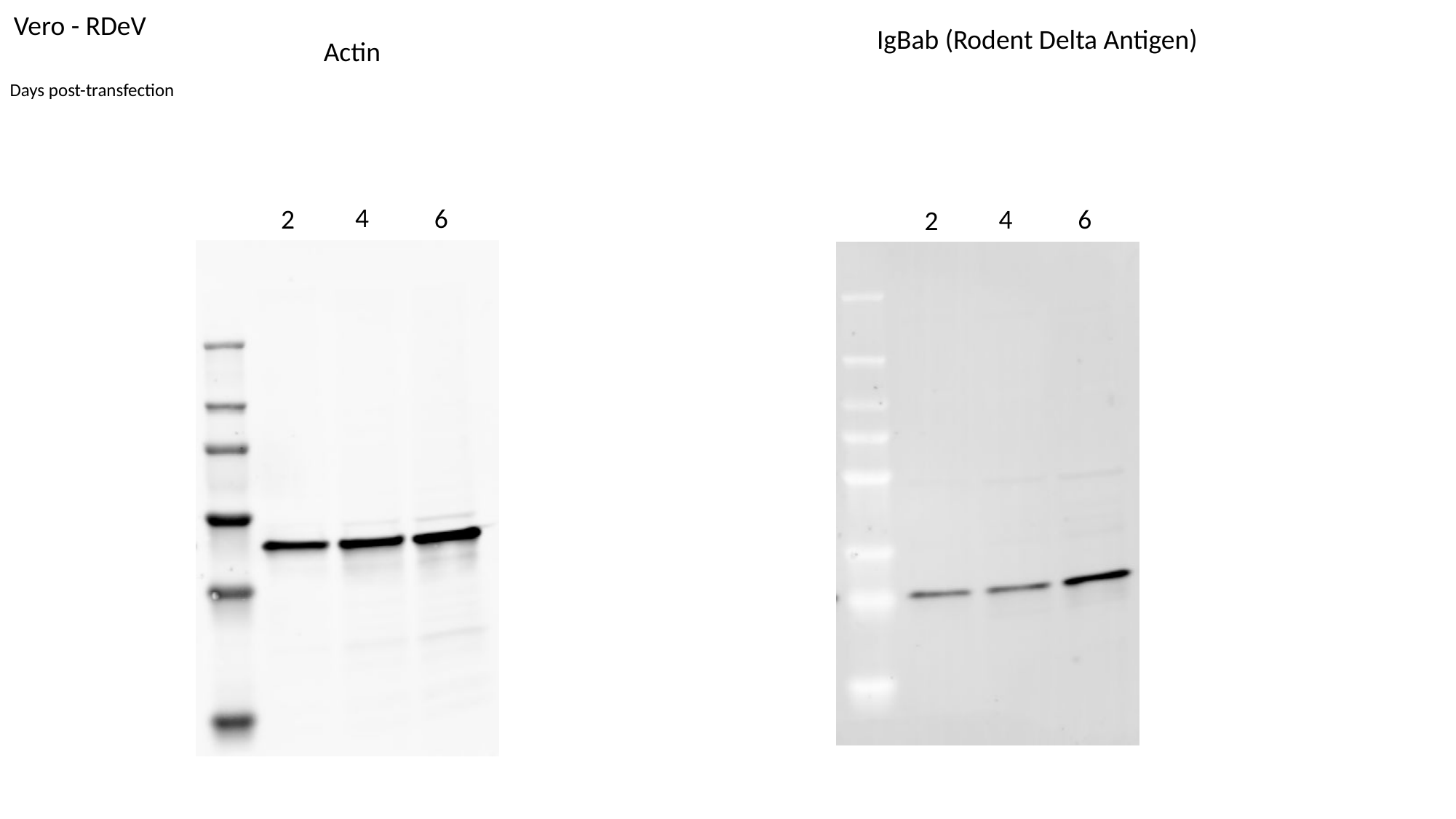

Vero - RDeV
IgBab (Rodent Delta Antigen)
Actin
Days post-transfection
4
6
4
2
6
2

## Slide 24
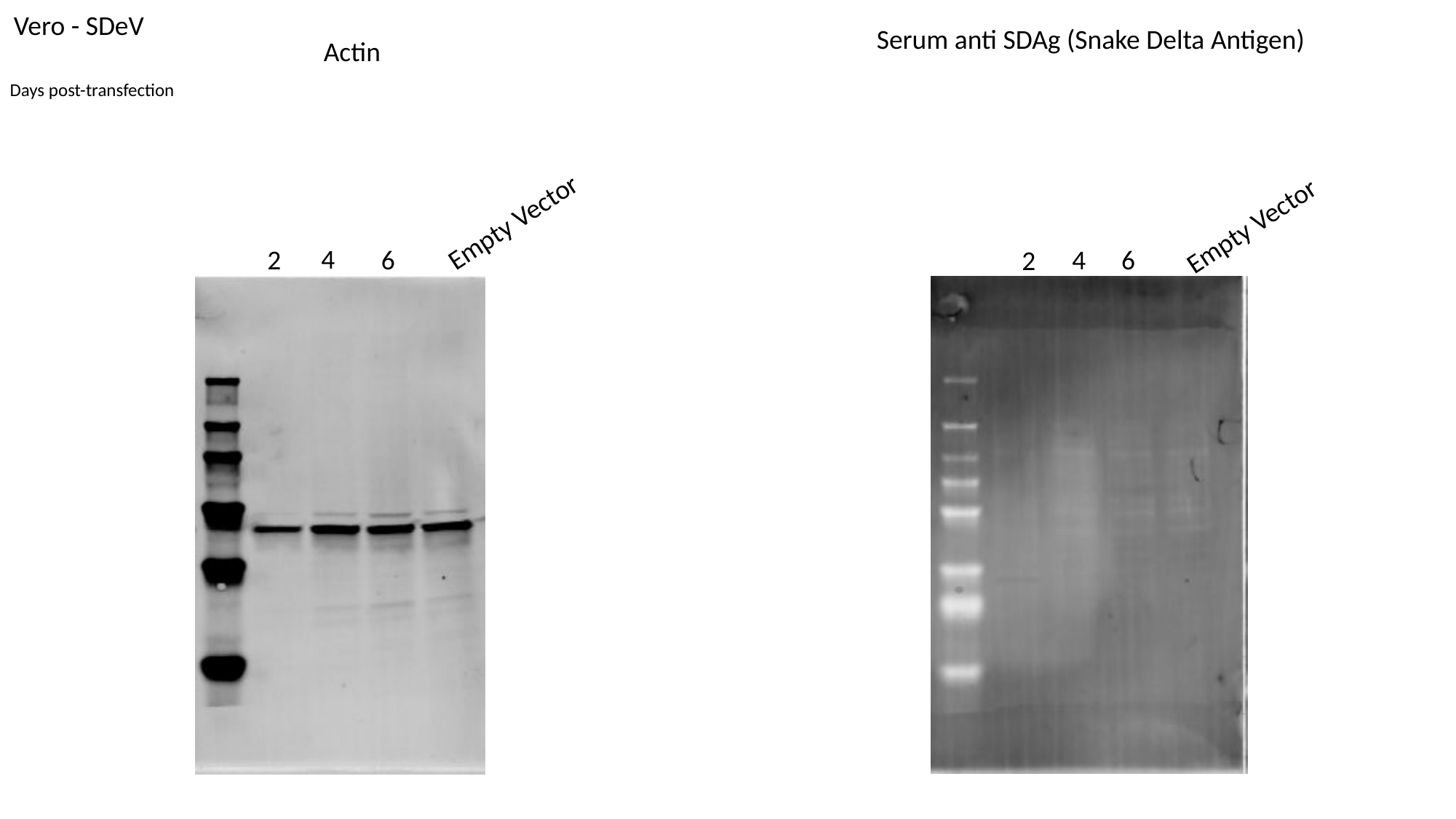

Vero - SDeV
Serum anti SDAg (Snake Delta Antigen)
Actin
Days post-transfection
Empty Vector
Empty Vector
4
2
4
6
6
2

## Slide 25
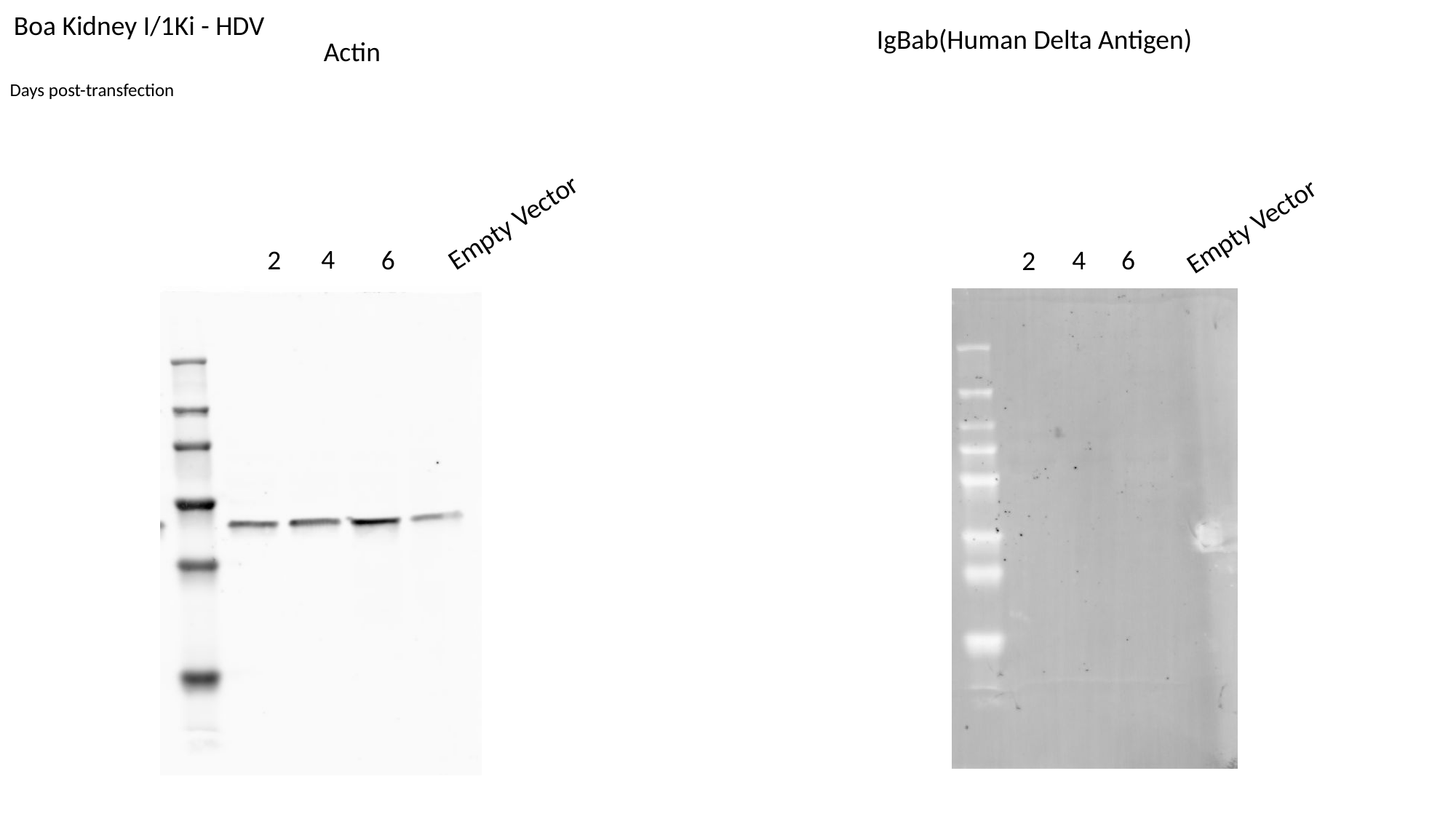

Boa Kidney I/1Ki - HDV
IgBab(Human Delta Antigen)
Actin
Days post-transfection
Empty Vector
Empty Vector
4
2
4
6
6
2

## Slide 26
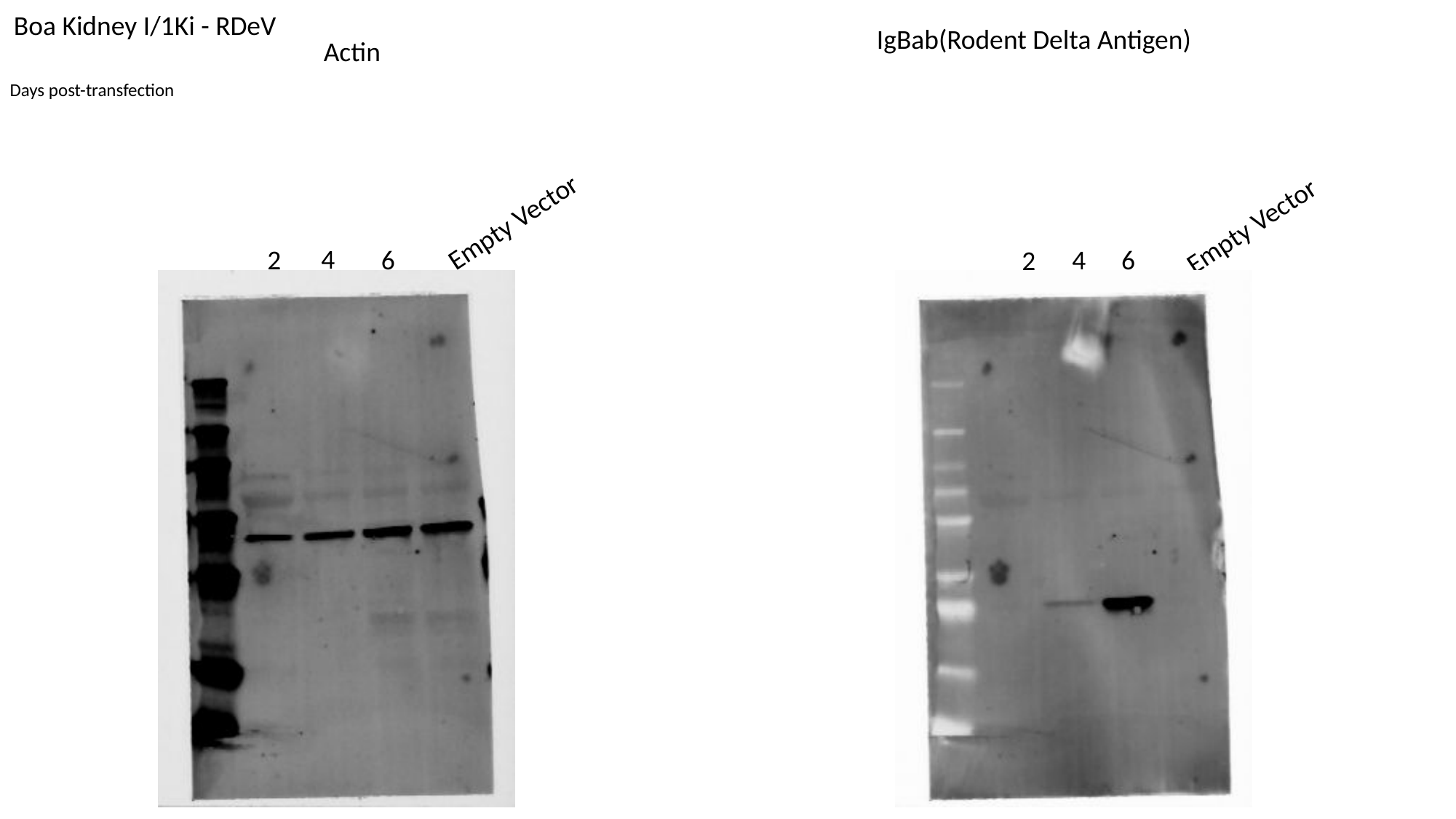

Boa Kidney I/1Ki - RDeV
IgBab(Rodent Delta Antigen)
Actin
Days post-transfection
Empty Vector
Empty Vector
4
2
4
6
6
2

## Slide 27
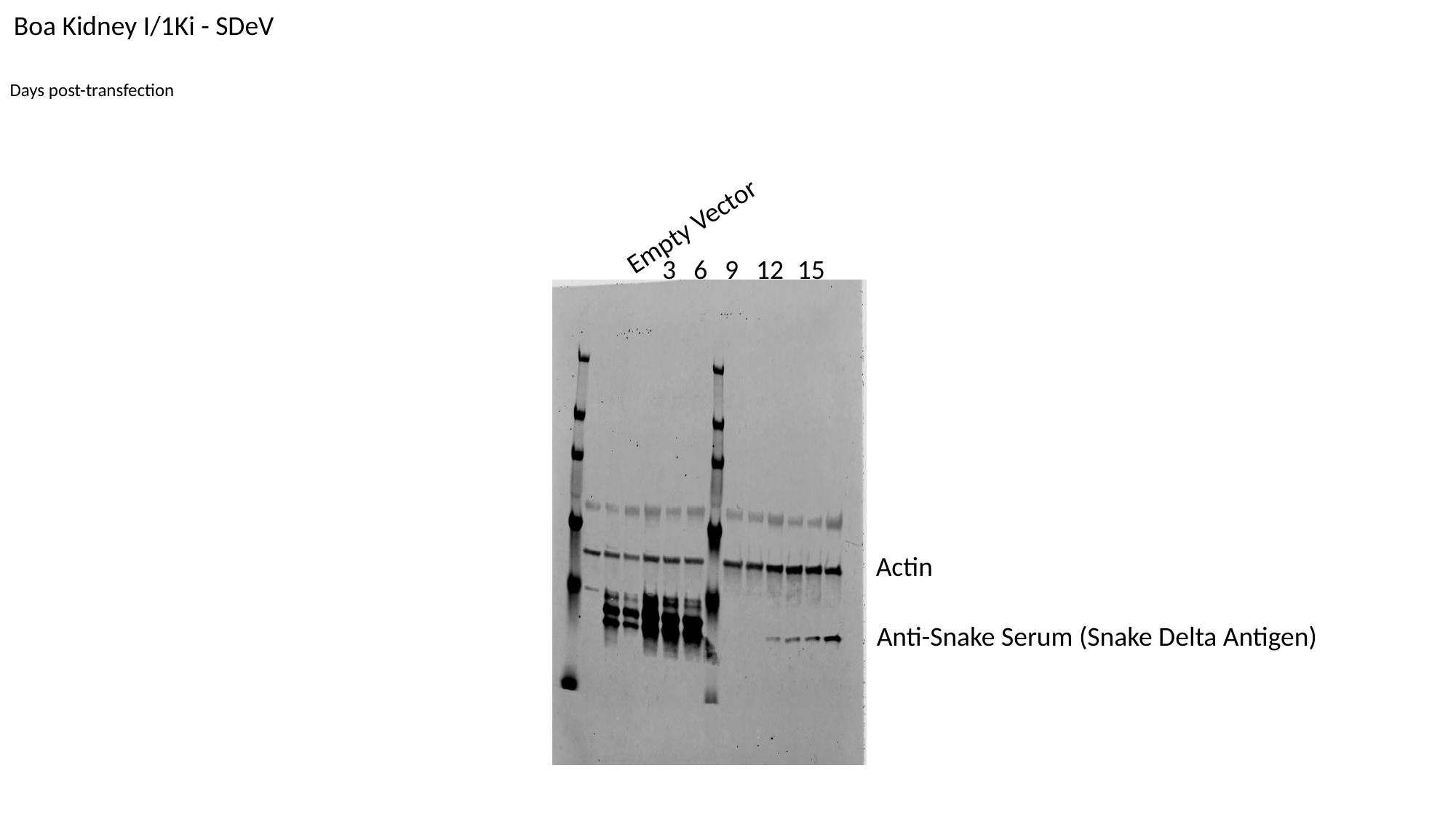

Boa Kidney I/1Ki - SDeV
Days post-transfection
Empty Vector
3
6
9
12
15
Actin
Anti-Snake Serum (Snake Delta Antigen)
